# Allometric escape from acoustic constraints is rare for frog calls

**DOI:** 10.1002/ece3.6155

**Published:** 2020-03-07

**Authors:** João Filipe Riva Tonini, Diogo B. Provete, Natan M. Maciel, Alessandro Ribeiro Morais, Sandra Goutte, Luís Felipe Toledo, Robert Alexander Pyron

**Affiliations:** ^1^ Department of Biological Sciences The George Washington University Washington DC USA; ^2^ Museum of Comparative Zoology Department of Organismic and Evolutionary Biology Harvard University Cambridge MA USA; ^3^ Setor de Ecologia Instituto de Biociências Universidade Federal de Mato Grosso do Sul Mato Grosso do Sul Campo Grande Brazil; ^4^ Gothenburg Global Biodiversity Centre Göteborg Sweden; ^5^ Departamento de Ecologia Instituto de Ciências Biológicas Universidade Federal de Goiás Goiânia Brazil; ^6^ Laboratório de Biologia Animal, Instituto Federal Goiano Rio Verde Brazil; ^7^ Laboratório de História Natural de Anfíbios Brasileiros Departamento de Biologia Animal Instituto de Biologia Universidade Estadual de Campinas Campinas Brazil; ^8^ New York University Abu Dhabi Abu Dhabi UAE

**Keywords:** adaptive evolution, advertisement call, anurans, evolution, phylogenetic comparative methods

## Abstract

Allometric constraint is a product of natural selection and physical laws, particularly with respect to body size and traits constrained by properties thereof, such as metabolism, longevity, and vocal frequency. Allometric relationships are often conserved across lineages, indicating that physical constraints dictate scaling patterns in deep time, despite substantial genetic and ecological divergence among organisms. In particular, acoustic allometry (sound frequency ~ body size) is conserved across frogs, in defiance of massive variation in both body size and frequency. Here, we ask how many instances of allometric escape have occurred across the frog tree of life using a Bayesian framework that estimates the location, number, and magnitude of shifts in the adaptive landscape of acoustic allometry. Moreover, we test whether ecology in terms of calling site could affect these relationships. We find that calling site has a major influence on acoustic allometry. Despite this, we identify only four major instances of allometric escape, potentially deriving from ecomorphological adaptations to new signal modalities. In these instances of allometric escape, the optima and strength of the scaling relationship are different than expected for most other frog species, representing new adaptive regimes of body size ~ call frequency. Allometric constraints on frog calls are highly conserved and have rarely allowed escape, despite frequent invasions of new adaptive regimes and dramatic ecomorphological divergence. Our results highlight the rare instances in which natural and sexual selection combined can overcome physical constraints on sound production.

## Graphical abstract

Allometric constraints in frogs are highly conserved and have rarely promoted allometric escape despite frequent invasions of new adaptive regimes of sound frequency and dramatic ecomorphological divergence in body size and calling site. In this study, we highlight instances in which natural and sexual selection combined could overcome physical constraints on sound production.

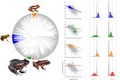

## 1 INTRODUCTION

Allometry is the study of correlates and consequences of changes in body size affecting physiological, anatomical, or behavioral traits (West & Brown, 2000). Allometric constraints are among the most deeply conserved features in living systems (West & Brown, 2000), along with body plans (Arthur, 1997), and reproductive modes (Wake, 2003). Scaling relationships with body size, such as metabolism (Uyeda, Pennel, Miller, Maia, & McClain, 2017) and brain volume (Gould, 1971) are shared by uni‐ and multicellular organisms, comprising billions of years of evolutionary history. The rigidity of these constraints derives primarily from their origin in physical laws (e.g., quarter‐power law), rather than the sole influence of natural selection due to biological factors (West & Brown, 2000).

However, sexually selected characters that are under reduced physical constraint often vary more widely. Examples include traits involved in display signals among individuals of the same species, such as Irish elk (antlers; Gould, 1974), or across larger species groups, such as swordtails (caudal fins; Meyer, Morrissey, & Schartl, 1994), peacocks and other birds (tail feathers; Aparicio, Bonal, & Cordero, 2003), cichlids (fin shapes and colors; Wagner, Harmon, & Seehausen, 2012), and angiosperms (flower morphology; Willson, 1990). As characters with physical, biological, and sexual components, acoustic signals are traits that mediate species recognition, induce reproductive isolation, and drive speciation (Ryan, 1988a). The sonic frequency of these signals is typically constrained by body size (Gerhardt, 1994). In species that vocalize, larger individuals generally have larger larynxes (or other organs), longer vocal folds that oscillate at lower frequencies, and longer vocal tracts that produce lower resonances (Nevo & Schneider, 1976; Ryan, 1988a, 1988b). This pattern produces a well‐known acoustic allometric relationship with larger individuals producing lower dominant frequencies in frogs, birds, and mammals (Bradbury & Vehrencamp, 1998; Fletcher, 2004; Ryan, 1988a).

For frogs in particular, character displacement in calls is a primary mechanism for speciation (Blair, 1964). As such, frog calls are among the most diverse sexual signals in the animal kingdom, spanning orders of magnitude in pitch, duration, timbre, and intensity. Most male frogs emit advertisement calls during mating events, making it the most commonly emitted call. This type of call is under both natural and sexual selection (Nevo & Schneider, 1976), since ecological factors can drive call evolution and call structure provides information about male quality, directly affecting mate choice. Furthermore, female choice plays a role in shaping the diversity of sound frequency as well (Moreno‐Gómez, Bacigalupe, Silva‐Escobar, & Soto‐Gamboa, 2015; Rand, 1985). Specifically, the sonic frequency of calls allows mate recognition and generally matches with female tympanic range (Ryan, 1988a). Frog's advertisement calls can present some plasticity based on temperature or presence of conspecifics, but overall these calls tend to be innate, usually stereotyped due to its role in species recognition; thus, presumably have a genetic component as they do in birds (Wheatcroft & Qvarnström, 2017).

Among sources of sonic constraints, background noise and properties of the vegetation structure affect transmission, integrity, and detection, and consequently female ability to decode and react to males’ signal (Lengagne, 2008; Morton, 1975). As a result, some species breeding in noisy environments, such as fast‐flowing streams and torrents, have adapted to produce sounds in distinct frequency than the background, such as ultrasonic calls or develop new behaviors, such as multimodal signals to attract females (Arch, Grafe, & Narins, 2008; Haddad & Giaretta, 1999), which could potentially alter scaling relationships. Furthermore, calling site such as floating or submerged in water, from the ground, or from a perch interacts with body size, potentially representing an additional selective agent to disrupt acoustic allometry (Gerhardt, 1994; Yager, 1992). For example, a shift to arboreal life tends to drive lineages toward an optimum size shared by most arboreal species that calls perching on vegetation (Emerson, 1991). A recent study also found that call structure (either tonal or pulsed) is related to calling site in glass frogs (Escalona Sulbarán, Simões, Gonzalez‐Voyer, & Castroviejo‐Fisher, 2019), suggesting both calling site and body size could constrain call evolution.

In addition, calling site also affects frequency due to adaptations for signal transmission through surrounding obstacles, such as foliage, terrain, or water (Ryan, 1988b). For example, clawed frogs of the family Pipidae live and call underwater. Surprisingly, these species have retained a terrestrial respiratory tract, while the larynx has evolved highly modified structures relative to most frogs breeding on land (Yager, 1992). Thus, major shifts in ecomorphology across frogs, such as transitions or reversions from terrestrial, arboreal, or aquatic habitat that lead to changes in physical structures related to calling may promote allometric escape as well.

The invasion of new adaptive regimes (Stroud & Losos, 2016) of acoustic allometry as a result of ecological opportunity provided by ecomorphological and sexual pressures affects call diversity. Additionally, call diversity is shaped by the physical constraints of body size (Fletcher, 2004; Pélabon et al., 2014) and larynx volume (Garcia, Herbst, Bowling, Dunn, & Fitch, 2017) that operate broadly on sounds. Nonetheless, it is unclear whether the vast diversity of call frequencies across frogs would adhere to a single allometric scaling relationship, or whether natural or sexual selection has promoted change in allometry to new selective optima for frequency‐size relationships in different lineages experiencing distinct selection regimes. Macroevolutionary shifts in scaling—allometric escape—may thus be driven by the evolution of novel morphological or physiological mechanisms related to vocalization (Gerhardt, 1994; Haddad & Giaretta, 1999), as well as shifts in calling site, colonization of new habitats with an altered acoustic space (Gerhardt, 1994; Ryan & Brenowitz, 1985), or behavioral changes related to sexual selection (Charlton & Reby, 2016).

Here, we use a phylogenetic framework to test the hypothesis of allometric conservatism in the face of varying selective agents such as calling site and acoustic competition. We ask how many instances of allometric escape have occurred across the frog tree of life using a Bayesian framework that estimates the location, number, and magnitude of shifts in the adaptive landscape of acoustic allometry (Uyeda & Harmon, 2014; Uyeda et al., 2017; Zanne et al., 2017). Our prediction is that most frogs will adhere to a common size‐frequency allometric regime—as expected, but that some clades may have escaped this relationship, due to the mechanisms described above. We use a taxonomically complete phylogeny (Jetz & Pyron, 2018) and data on mean dominant frequency (DF) of male advertisement calls, mean male body size, hereafter as body size, and calling site (Appendix S1). The final dataset includes information for 2,176 species (28% of the total species‐level frog diversity), from 293 genera (65% of genus‐level diversity) and 42 families (77% of frog family‐level diversity) distributed worldwide (see Appendix S1).

## 2 MATERIALS AND METHODS

### 2.1 Calling site and bioacoustics data

We collected data on mean dominant frequency (DF), hereafter referred as frequency, of advertisement calls and male snout‐vent length (hereafter referred to as a measure of body size) of anuran species from the literature and museum collections (Appendix S2). The DF of male advertisement calls represents the highest peak in energy (or amplitude), and it has greater variation between than within species. In fact, it can vary over one order of magnitude among species at the family level. Moreover, DF is not affected by ambient temperature, unlike temporal variables (Köhler et al., 2017), and it is a static, often reported, and relatively uncontroversial characteristic to measure in frog calls (Köhler et al., 2017). Although different Fast Fourier Transform—method that accelerates the calculations by discretizing the time domain into multiple fragments of sound (Köhler et al., 2017)—parameters could lead to dissimilarities for the same call, particularly number of harmonics and temporal patterns, DF is the parameter that changes less (Köhler et al., 2017). The majority of data on mean DF was extracted from published studies on advertisement calls (Appendix S2), but for species missing this information in the scientific literature, we measured dominant frequency directly from audio files using FFT 512.

Large‐scale analyses show that the effect of body size on fundamental frequency—lowest or first harmonic—is similar to the effect of body size on DF (Gingras, Boeckle, Herbst, & Fitch, 2013; Gingras, Mohandesan, Boko, & Fitch, 2013). Although DF is a suitable trait to investigate shifts in allometric constraint in a macroevolutionary scale, it comes with limitations. It is unclear whether DF when not overlapping with fundamental frequency would be correlated to morphological structures involved in sound production (Gingras, Boeckle, et al., 2013; Gingras, Mohandesan, et al., 2013; Trewavas, 1932). Moreover, simplifying the spectrum portion of males’ advertisement call to a single continuous trait may obscure nuances of call variation. We try to mitigate the possibility of variation around body size and DF by including in the macroevolutionary model a parameter for measurement error estimated from the studies included in Appendix S2 that accounted for intrapopulation variation.

Calling site is a trait likely correlated with both body size and frequency due to the high competition for physical and acoustic space (Gerhardt, 1994). It is also typically well characterized for most species (Crump, 1974; Salthe & Duellman, 1973). Therefore, we classified 2,176 frog species according to their preferred calling site into three categories (Appendix S1): (a) aquatic, for frogs calling while floating or submerged; (b) terrestrial, for frogs calling on the ground, leaf‐litter, sitting on shallow pools, side of streams, or rocks in streams; and (c) arboreal, for frogs calling on vertical surfaces, trees, and herbaceous vegetation (Haddad, Toledo, Prado, Loebmann, & Gasparini, 2013; Rodríguez et al., 2015). We note that only 28% of all frog species were included in the present analysis. Thus, it is possible that future analyses sampling call data from more species may uncover additional significant shifts, or more complex interactions across the phylogeny regarding calling site and other variables.

### 2.2 Phylogenetic inference

The phylogenetic dataset comprises 7,238 species of amphibians, taken from the 19 February 2014 edition of AmphibiaWeb database and representing the number of described species of amphibians at that time (Jetz & Pyron, 2018). In all further analyses, we used one representative phylogeny from the posterior distribution of 10,000 fully sampled (7,238 species) trees, and the summary data‐only (4,061 species) tree. Taxonomic issues within genera for this phylogenetic dataset have been identified by previous authors (Padial, Grant, & Frost, 2014) but these do not change the coding of the character states. The use of one fully sampled tree is justified by the computational intensity of analyses performed (see below).

The fully sampled phylogenies were generated using the PASTIS method, which uses species imputation techniques to include in the phylogeny species without molecular data available (Jetz & Pyron, 2018). Whereas, in the data‐only phylogeny the species relationship was recovered using molecular data, leading to the reduced sampling (Jetz & Pyron, 2018). We used the fully sampled, time‐calibrated phylogeny of amphibians pruned to include the 2,176 species with information on body size, sound frequency, and calling site (Appendix S1). The data‐only phylogeny included 1,610 species to which we had trait data. Major taxonomic differences between the data used in the analyses of the fully samples and data‐only phylogenies are in the families Leptodactylidae (41% fewer species in the data‐only compared to the PASTIS), Microhylidae (39%), Hyperoliidae (38%), Myobatrachidae (37%), Hylidae (32%), and Bufonidae (21%). PASTIS and data‐only phylogeny recovered similar results but since the PASTIS includes a larger number of species, we include these results in the main text and the data‐only phylogeny results in Appendix S3.

### 2.3 Phylogenetic comparative analyses

The Ornstein‐Uhlenbeck (OU) macroevolutionary model incorporates the idea of stabilizing selection, allowing lineages to shift their optimum trait value when occupying new regimes (Butler & King, 2004; O'Meara, 2012) in an adaptive landscape. Body size and advertisement call frequency are traits under natural and sexual selection; thus, the OU model is suitable to estimate shifts in acoustic allometry. Frog advertisement calls have been a model system to study species communication, given their biological significance and relative simplicity (Blair, 1964; Gerhardt & Huber, 2002; Hoskin, James, & Griggs, 2009; Ryan, 1980). In contrast, birds and mammals have higher learning capability and can often produce multiple sounds that might not reflect long‐term evolutionary relationships (Raposo & Höfling, 2003). Furthermore, larger males are known to have higher survivorship and fertilization rates; thus, frequency of advertisement calls transmits information on fitness benefits (Wells, 2007).

The OU model is useful to describe the phenomenon of body size and call frequency diversification that happens during frog adaptive radiations (Stroud & Losos, 2016). Within each identified adaptive regime of acoustic allometry, we might expect variation (σ^2^, sigma squared) around the optimum intercept value (θ, theta) and the slope (β, body size ~ frequency) describing the allometric scaling relationships associated with these factors (Ohmer, Robertson, & Zamudio, 2009). The alpha (α) parameter of the OU model is usually interpreted as the “selection strength” that pulls the trait value back toward its optimum value and also controls the amount of variation around it. Simulation studies have shown that parameter estimates for OU models can be bias, and the estimated values in this study should be interpreted with caution (Ho & Ané, 2014).

We used a Bayesian phylogenetic framework implemented in the R (R Core Team, 2019) package *bayou 2.0* (Uyeda & Harmon, 2014; Uyeda et al., 2017) to detect regime shifts in acoustic allometry scaling between DF, body size, and calling site. The model implemented in *bayou* uses a reversible‐jump Markov Chain Monte Carlo (rjMCMC) algorithm that automatically estimates the location, number, and magnitude of shifts in adaptive regimes in a phylogeny (Uyeda & Harmon, 2014) without the need to define the clades a priori. The rjMCMC produces a full posterior of credible models and parameter values, incorporating uncertainty in the estimates (Uyeda & Harmon, 2014).

We log‐transformed values of DF and body size to achieve normality and fit three linear models (following reference Uyeda, Pennel, Miller, Maia, & McClain, 2017): (a) θ_DF_ ~ β_body size_ and (b) θ_DF_ ~ β_body size_ + β_sit_, and (c) θ_DF_ ~ β_body size_ + β_sit_ + β_body size*sit_ that estimated the intercept (θ for DF), slope (β) of the linear relationship between sound frequency (θ_DF_), body size (β_body size_), calling site (β_sit_), and their interaction (β_body size*sit_). We included a parameter in the model to account for Measurement Error (ME) derived from the studies including intraspecific variation (Appendix S2). We also estimated parameters of the OU model, such as per‐unit‐time magnitude of the uncorrelated diffusion (σ^2^), the strength of selection (α), and the number of adaptive regimes (K). We set up the K prior maxima to 200 across all models. Then, we used Bayes Factor for model comparison. Bayes Factor (BF) higher than 10 was considered strong support toward a given competing model (Kass & Raftery, 1995).

We used a flat prior for the slope, following a standard normal distribution with 0.2 standard deviation (Uyeda et al., 2017). We used a distribution with the log mean DF and 1.5 times the standard deviation as prior for the intercept (Uyeda et al., 2017). We ran four independent MCMC chains with 10–20 million generations with different random starting seeds, sampling every 1,000th generation, yielding a distribution of minimum ten thousand samples of the posterior probability, and used as burn in the first 25% of the samples. We checked for convergence between the four chains using Gelman's R (Gelman & Rubin, 1992) by comparing the posterior probabilities of branches (Figure S2). Chains converging were combined to increase the sampled parameter space and Effective Sample Sizes (ESS) (see in Appendix S3 Table S1–S6).

Shifts with posterior probabilities equal or higher than 0.7 were considered well‐supported and interpreted as adaptive shifts in acoustic allometry scaling. This is a conservative threshold applied in previous studies using *bayou* (Cuff et al., 2015; Uyeda & Harmon, 2014; Uyeda et al., 2017). We consider that this is an important value given the potential issues of power and parameter estimates related to the OU model pointed out recently (Cooper, Thomas, Venditti, Meade, & Freckleton, 2016; Cressler, Butler, & King, 2015; Ho & Ané, 2014), as well as birth–death trees in particular (Rabosky, 2015; Title & Rabosky, 2016), and inherent to the trait data (see above). Priors can have a large influence on the results in Bayesian implementations of the OU models (Cooper et al., 2016; Ho & Ané, 2014). Therefore, we ran the Bayesian estimation procedure with no data to check the mean number of shifts a priori. As a conservative approach in trying to highlight major changes in acoustic allometric escape that were shared by more than two common ancestors, shifts including three or less species were not included in the main text.

The analyses run for 70,080 computer‐hours on the high‐performance computing cluster “Colonial One” at The George Washington University, until parameter convergence was reached. We provide annotated code in Appendix S4.

To further use a Null Hypothesis Significance Test (NHST) approach for testing the “significance” of the shifts recovered in the Bayesian analysis, we used a phylogenetic Analysis of Covariance (pANCOVA; Smaers & Rohlf, 2016). This additional test is a simple Phylogenetic Generalized Least Squares (PGLS) that assumes Brownian Motion used to test for deviations in slope and intercept of dominant frequency and body size for each lineage recovered as distinct shift in this allometric relationship. In the Full Model, we included four parameters, one intercept and one slope for the given lineage identified as a shift and another intercept and another slope for the remaining species represented in the phylogeny. Then, we contrast the Full Model with a Reduced Model, in which we include only two parameters—one intercept and slope for all species in the phylogeny. Model comparison is accomplished by means of a F‐ratio test. Analysis was conducted in *evomap* R package (Smaers, 2014).

## 3 RESULTS

**Figure 1:**
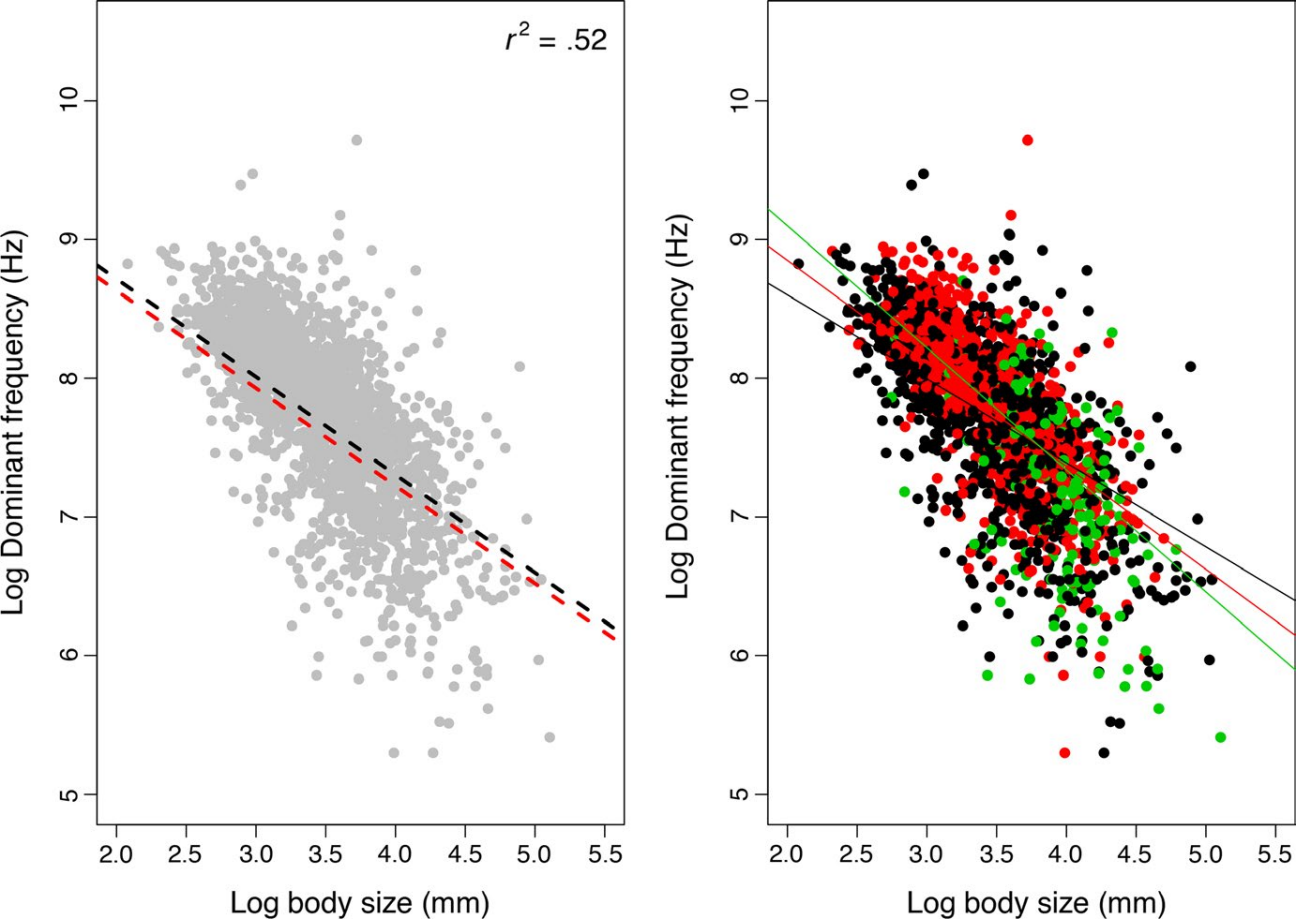
Allometric relationship between log dominant frequency (Hz) and log body size (mm) across 2,176 frog species included in this study. The left panel shows the phylogenetic generalized least squares in red and linear regression in black. Note that body size alone explains 51% of the variation in dominant frequency (inset: value of R^2^ from the best fit model). The right panel shows the different allometric scaling for each calling site estimated by the best fit model in *bayou*. In black, species calling from the ground; in red, species that perch while emitting advertisement calls; and in green, species that call while sitting, swimming, or submersed in water

Our results show that the model including the interaction between body size and calling site (θ_DF_ ~ β_body size_ + β_site_ + β_body size*site_; Marginal Likelihood = −702.08), provided a better fit to the data according to Bayes Factors over the competing models—θ_DF_ ~ β_body size_ (Marginal Likelihood = −778.09) and θ_DF_ ~ β_body size_ + β_site_ (Marginal Likelihood = −796.68) (Figure S3). Although this suggests that calling site alone may not be a strong selective force changing call frequency, the effect of calling site is associated to body size (Figures S1, S5)—given that the model θ_DF_ ~ β_body size_ + β_site_ + β_body size*sit_ is the best fit to the data—which in turn affects call frequency (Figure 1). Thus, the reduced effect of calling site alone on sound frequency might represent the potential reason why so many clades do not shift to new adaptive optima even though they have a large variance in call frequency. The most complex model was preferred (θ_DF_ ~ β_body size_ + β_sit_ + β_body size*sit_) but it recovers the similar shifts in acoustic allometry as the simplest model including just frequency and body size (θ_DF_ ~ β_body size_; Appendix S4). This result suggests that the factors driving shifts have evolved in concert, rather than in isolation.

**Figure 2:**
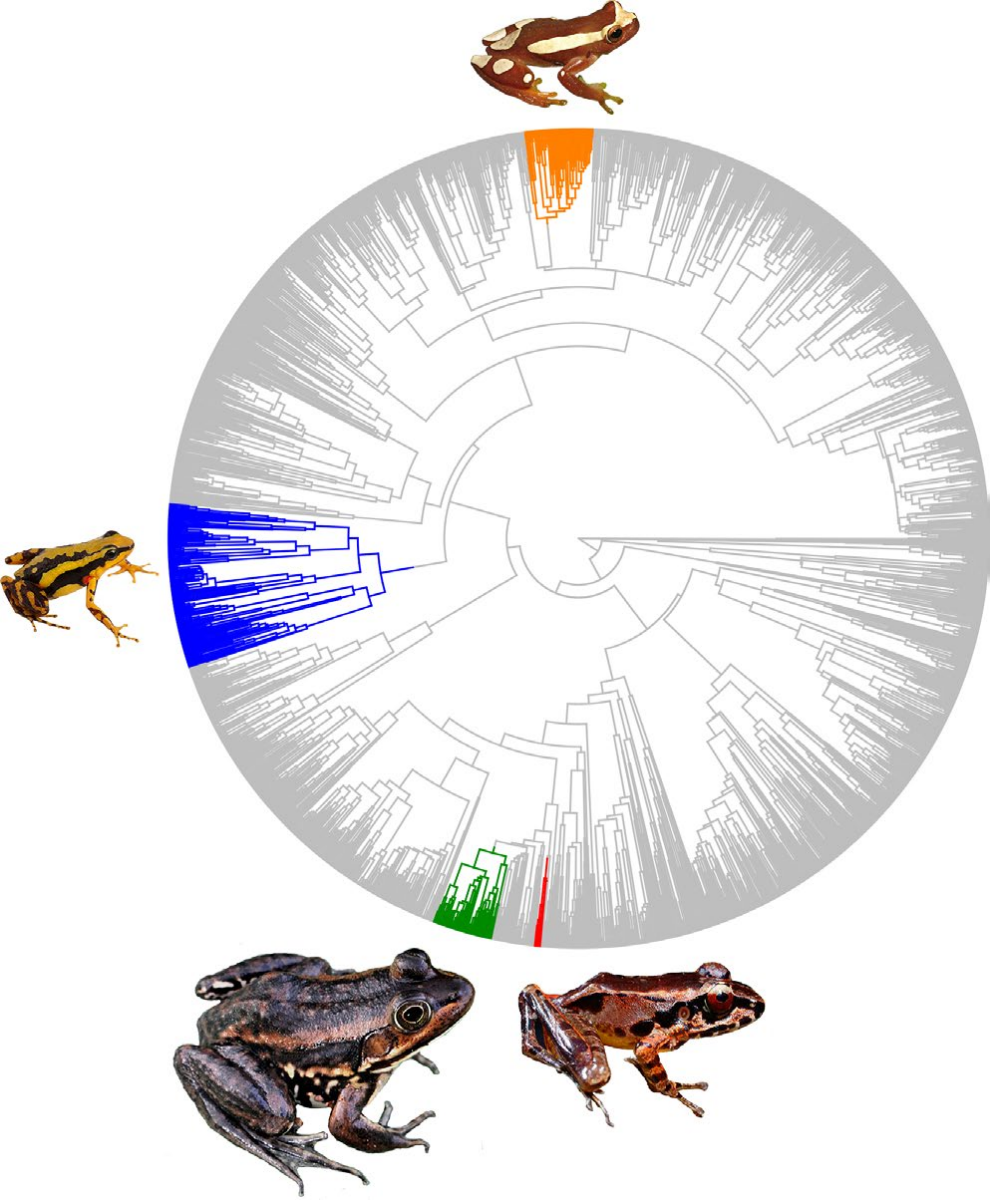
Acoustic allometry scaling regimes mapped on the species phylogeny. In gray is the ancestral relationship shared by most frog species. Colors represent distinct allometric escapes identified by the macroevolutionary model with posterior probabilities > 0.7 and supported by the pANCOVA. Red: Southeast Asian ranids (*Huia cavitympanum*); Blue: Neotropical poison frogs (*Epipedobates tricolor*); Green: ranid frogs (*Rana blairi*), and orange: Fitzinger Neotropical Tree frogs (*Dendropsophus elegans*). Frogs are scaled to relative size

Surprisingly, the adaptive landscape of the body size‐frequency allometric scaling in frogs is complex. Overall, the vast majority (88%) of frog species included here share an ancestral regime for acoustic allometry scaling (Figure 2), a pattern that has been already partially documented (Fletcher, 2004). However, we recovered at least four major instances of allometric escape of sound frequency that were supported by both *bayou* and pANCOVA. The best fit *bayou* model identified 26 acoustic allometric regimes across frogs, but only four of those were also supported by pANCOVA as distinct from the ancestral regime (Figure 2, Table 1; see Appendix S4 for identification of shifts under multiple posterior probability thresholds). These shifts range in age from 33 Ma (Southeast Asian ranids, in red; Figure 2) to 103 Ma (poison frogs, in blue; Figure 2) with a mean of 84.94 Ma and median of 42.9 (standard deviation = 72.61; Table 2), and in diversity from a few endemic species (Southeast Asian ranids, in red; Figure 2, Table 2) to hundreds of species (poison frogs; Figure 2) (Table 2). Of these four major instances, only the poison frogs (blue clade) are particularly old or diverse (Table 2). In addition, we confirm that the allometric scaling of body size and frequency in extant frog species is otherwise conserved across 219 million years of evolutionary history, despite both variables covering several orders of magnitude (Figure 1) in nearly every habitat and ecoregion worldwide. This result suggests that a single adaptive allometric scaling constrains the body size ~ frequency relationship across most lineages, despite the putative ecological and evolutionary processes that disrupt the constraint of body size on sound frequency (Gerhardt, 1994; Haddad & Giaretta, 1999; Ryan & Brenowitz, 1985). Moreover, our results illustrate a previously hidden interplay between natural and sexual selection that leads to shift in allometric scaling of sound producing.

**Table 1:** Results of phylogenetic ANCOVA for testing the significance of the relationship between dominant frequency and body size identified in the *bayou* model

Clades	Models	DF	Sum Squares	Mean Sum of Squares	F	Pr(>F)
Southeast Asian ranids	Full model	4	4,003.601	1.843	3.769	0.023
	Reduced model	2	4,017.494	1.848		
Ranid frogs	Full model	4	3,967.213	1.827	13.764	0.000
	Reduced model	2	4,017.494	1.848		
Fitzinger Neotropical Tree frogs	Full model	4	3,995.990	1.840	5.844	0.003
	Reduced model	2	4,017.494	1.848		
Poison frogs	Full model	4	3,993.421	1.839	6.547	0.002
	Reduced model	2	4,017.494	1.848		

Note

The column Model represents the result of F‐ratio Test between the model without differences in slope and intercept (Reduced Model) and the model allowing one slope and a distinct intercept to the respective lineage (Full Model). Showing results of pANCOVA for *p* < .05 out of 26 total shifts in *bayou*. Colors correspond to those shown in Figure 2.

Abbreviation: DF, degrees of freedom.

**Table 2:** Optima in log dominant frequency (θ_DF_) identified by *bayou*, slope of the regression between DF and body size (β_body size_) across allometric regimes, Mean Age (Ma) shifts, N species shifts, and N species sampled at the family level

	θ_DF_	β_body size_	Mean Age (Ma) shifts	*N* species shifts	*N* species sampled
Root (gray)	9.28	−0.45	205	1,938	2,176
Southeast Asian ranids	8.25	0.01	33.3	6	100
Poison frogs	9.98	−0.24	102.7	141	141
Fitzinger Neotropical Tree frogs	9.56	−0.16	42.9	59	440
Ranid frogs	8.69	−0.20	41	52	100

Note

Mean Age (Ma) shifts—Mean divergence age in millions of years of species included in the allometric regimes, *N* species shifts—number of species used in this study for each family with representatives identified as having allometric escape, *N* species sampled—number of species anurans sampled in this study broke down by family Ranidae (red and green clades) and Hylidae (orange clade), and the superfamily Dendrobatoidea (blue clade).

**Figure 3:**
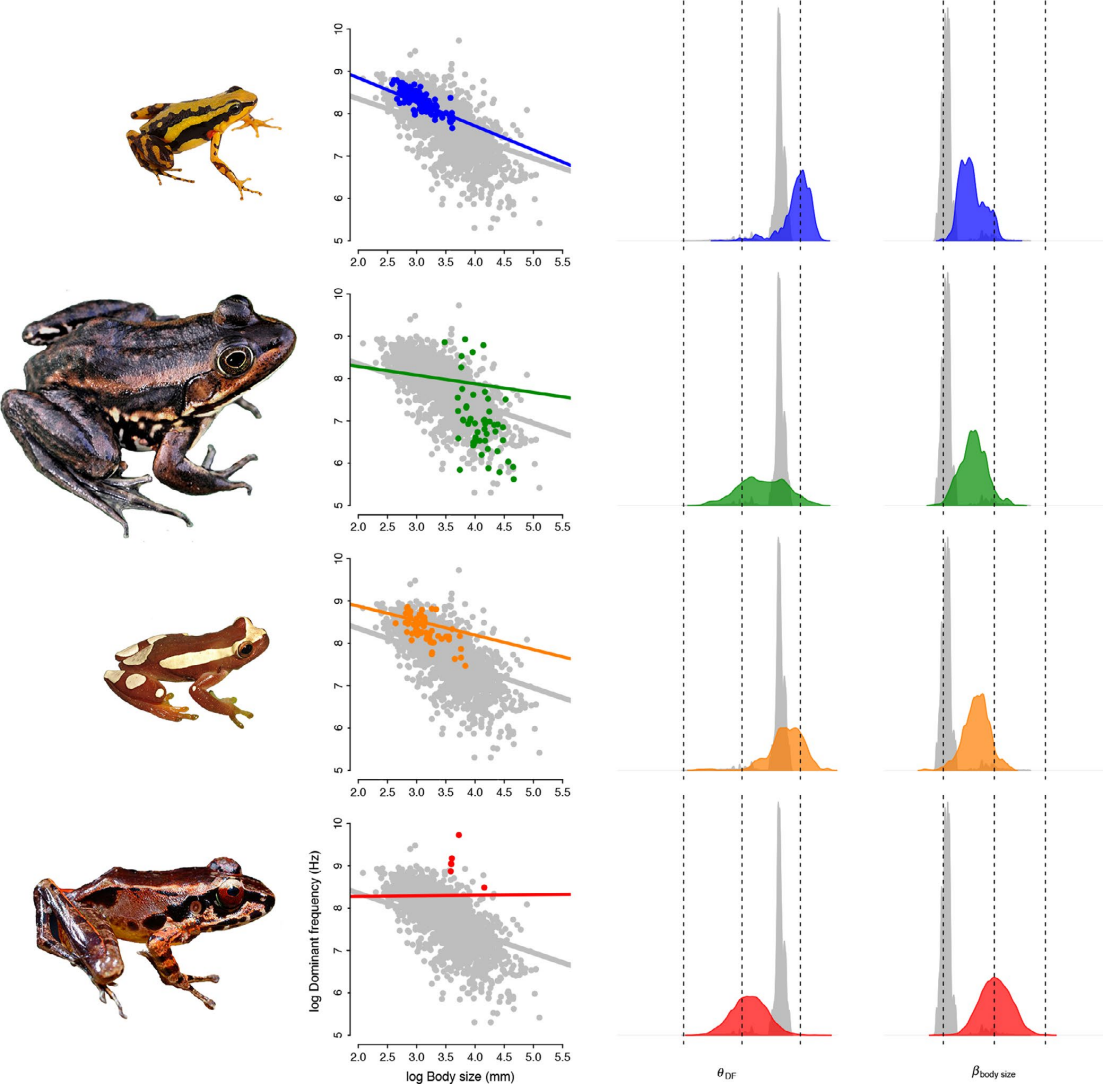
Allometric regimes for the four escaped frog lineages. In the left column, the y‐axis represents log Dominant frequency (Hz) and the x‐axis represents log body size (mm). Regression lines represent median intercept and slope estimated in *bayou* (Morton, 1975) for the best fit model (θ_DF_ ~ β_body size_ + β_sit_ + β_body size*sit_). Red: Southeast Asian ranids (*Huia cavitympanum*); Blue: Neotropical poison frogs (*Epipedobates tricolor*); Green: ranid frogs (*Rana blairi*), and orange: Fitzinger Neotropical Tree frogs (*Dendropsophus elegans*). In the center and right columns, density plots show uncertainty in model parameter estimates of intercept (θ_DF_) and slope (β_body size_), respectively. Frogs are scaled to relative size

Species of Southeast Asian ranids (red clade) exhibit near‐zero slopes for the allometric relationship, in contrast to the strong negative slope shared by most lineages (Figure 3, Table 2). Species belonging to these adaptive regimes have higher‐pitched calls at larger sizes (Figure 3). The near‐zero slope indicates a decoupling of size and frequency under the adaptive strategy that the clade has adopted. Thus, variance in frequency in these cases must be attributable to an additional, unobserved ecological or sexual factor such as directly from specialized adaptations to higher frequency calls in noisy environments (Schwartz & Bee, 2013; Vielliard & Cardoso, 1996).

Poison frogs (blue), *Dendropsophus* (orange), and ranids (green) have flatter slopes than species belonging to the background acoustic allometry regime (Figure 3, Table 2), indicating a decreased, but still significant impact of body size on call frequency. Contrastingly, poison frogs and *Dendropsophus* have an increased intercept, whereas ranids have a similar intercept to the species in the background regime. Thus, the adaptive strategies of Southeast Asian ranids (red clade in Figures 2, 3) decouple call frequency from body size, while those of poison frogs (blue clade in Figures 2, 3), *Dendropsophus* (orange clade in Figures 2, 3), and ranids (green clade in Figures 2, 3) remain size‐dependent at a reduced magnitude.

## 4 DISCUSSION

The underlying drivers of allometric escape appear to be complex. For instance, other species in our dataset also call along rivers and streams, habitats with high background noise, but were not recovered as belonging to distinct regimes. Glass frogs (Centrolenidae) call on vegetation above or on the side of streams, while other torrent frogs such as Hylodidae call from sand banks or rocks around rapids, but they were not recovered as having distinct acoustic allometric scaling. Similarly, we have included 19 clawed frog species (Pipidae) in the analyses and, despite their striking morphological innovations of the vocal sound apparatus as adaptation to underwater sound communication (Arch et al., 2008; Tobias, Evans, & Kelley, 2011), the family as a whole does not appear to represent a major instance of acoustic allometric escape in our macroevolutionary analyses (Uyeda & Harmon, 2014; Uyeda et al., 2017). Thus, any difference in these species from the background allometric constraints appears to be within the bounds of variation and stochastic evolutionary divergence in acoustic allometry.

Given the drastic amount of ecomorphological disparity and biogeographic complexity in these and other anuran lineages (Moen et al., 2013), it is unclear why allometric escape is limited to the few clades identified here. One potential mechanism for mediating the effect of body size on sound frequency is the indirect influence on sound production from auditory structures, since the size of hearing mechanisms also scales with body size (Hetherington, 1992). Thus, differential selection acting on the timing or rate of development of both sound‐producing organs and the inner and middle ears may be the mechanism disrupting ancestral allometric relationships, rather than ecomorphological disparity per se. Little is known about the ontogenetic development of the larynx and middle ear structures in most extant frog species (Trewavas, 1932). However, there is considerable variation in the timing (event heterochrony; (Webster & Zelditch, 2005) of the middle ear development among frog families, specifically, the stage of columella, middle ear cavity, and inner ear (Hetherington, 1987). For clades such as *Dendropsophus* (orange in Figures 2, 3) and poison frogs (blue in Figures 2, 3) that are not affected directly by environmental noise (e.g., torrents), this may be the main physical mechanism leading to allometric escape.

Some microevolutionary mechanism may allow populations to cross valleys in a complex, rugged adaptive landscape. One of those is Wright's shifting balance theory (Wright, 1982), which dictates that populations with small effective size, more prone to drift, can retain phenotypic traits not highly adapted to one regime. But when the effective population size increases again, its phenotype is more prone to the effects of selection and then could climb back to their original peak or to an unoccupied peak. Stream‐dwelling anuran species are known to have quite small population sizes (Arruda, Costa, & Recco‐Pimentel, 2017; Green, 2003; Narvaes & Rodrigues, 2005; Phillipsen, Funk, Hoffman, Monsen, & Blouin, 2011). Southeast Asian ranids (red clade in Figures 2, 3) recovered as a shift by the Bayesian analysis are species that occur in torrents and fast‐flowing streams (Arch et al., 2008). Thus, shifting balance theory could, at least partly, explain how these linages invaded new frequency optima.

Evolution toward distinct and novel adaptive size‐frequency allometric regimes appears to be rare, particularly for traits constrained by physical laws, such as vocalization. Frog calls illustrate this clearly. Despite their massive variety, complexity, and importance to sexual signaling and diversification (Ryan, 1988a), the fundamental scaling relationship for acoustic allometry rarely varies across lineages. Sound frequency is constrained at a fundamental level by body size, a pattern that is conserved across the entire frog phylogeny, over 219 million years of evolutionary time. In the light of these results, we hypothesize that the only escapes from this constraint seem to arise from three primary factors. The first is fundamental changes in acoustic space, which select adaptations such ultrasonic communication or semaphore signals (Arch et al., 2008; Haddad & Giaretta, 1999). The second is microhabitat transitions related to calling site, since anatomical adaptations necessitate physical changes in sound‐producing (Arch et al., 2008) or hearing structures (Grant & Bolívar‐G, 2014). The third is the occupation of new biogeographic realms (Köhler et al., 2017), where acoustic diversification is driven by sonic competitors or ecomorphological changes as in the second instance. However, further studies are needed to test these hypotheses in a regional context in areas where frog species belonging to the root regime co‐occur with species in the shifts. For instance, many frog lineages experiencing similar ecomorphological shifts retain unaltered size‐frequency scaling relationships, suggesting that as‐yet unexplained factors are the ultimate determinants of allometric escape. Moreover, given the potential genetic association to these innate signals, which are crucial for frog communication and speciation, genomes may help elucidate a yet‐unexplored genetic signal of acoustic allometric shifts and our results provide hypothesis from a phylogenetic framework as to what lineages are more likely to have the signal.

## AUTHOR CONTRIBUTIONS

JFRT, DBP, and RAP designed the study. JFRT, DBP, ARM, NM, SG, and LFT collected the data. JFRT, DBP, and RAP wrote the first draft of the manuscript. JFRT led the writing afterwards and conducted data analyses, assisted by RAP and DBP. All authors revised the text. Data and materials availability: All data needed to evaluate the conclusions in the paper are present in the paper or the supplementary materials.

## CONFLICT OF INTEREST

None declared.

## Supporting information


**Appendix S1**

**Appendix S2**

**Appendix S3**

**Appendix S4**

## Data Availability

Data and scripts deposited in Dryad (https://doi.org/10.5061/dryad.98sf7m0dz) and provided as appendixes.
