# Supplementary material for: Allometric escape from acoustic constraints is rare for frog calls
Source: Ecol Evol. 2020 Mar 7;10(8):3686–95. doi: 10.1002/ece3.6155 (PMC7160179; doi:10.1002/ece3.6155)
Supplement: Supplementary file 2 — Appendix S2 [file ECE3-10-3686-s002.docx]

**Data S2.** Citation list use to gather trait data.

Abrunhosa, P.A., Wogel, H., & Pombal, J.P. Jr. (2001) Vocalizações de quatro espécies de anuros do estado do Rio de Janeiro, sudeste do Brasil (Amphibia: Hylidae, Leptodactylidae). *Boletim do Museu Nacional*, 472, 1–12.

Abrunhosa, P. A., & Wogel, H. (2004). Breeding behavior of the leaf-frog Phyllomedusa burmeisteri (Anura: Hylidae). Amphibia-Reptilia 25:125-135.

Abrunhosa P. A., Pimenta B. V. S., Cruz C. A. G., & Haddad C. F. B. (2005). Advertisement calls of species of the *Hyla albosignata* group (Amphibia, Anura, Hylidae). *Arquivos do Museu Nacional* 63(2):275–282.

Acioli, E. C. S., & Toledo, L. F. (2008). Amphibia, Anura, Hylidae, Hypsiboas beckeri : filling gap and description of its advertisement call. *Check List*, *4*(2), 182–184. https://doi.org/10.15560/4.2.182

Akmentins, M. S., Bonduri, Y. V., Contreras, P., Francisconi, L. E., Massabie, P. J., & Santillán, J. (2014). Redescripción del canto de anuncio de *Gastrotheca gracilis* Laurent, 1969 (Anura: hemiphractidae) y primer registro para el Parque Nacional Campo de Los Alisos, Tucumán, Argentina. *Cuadernos de herpetología*, *28*(2), 147–152. http://www.scielo.org.ar/scielo.php?script=sci_arttext&pid=S1852-57682014000200008

Allison, A., & Kraus, F. (2000). A new species of frog of the genus Xenorhina (Anura: Microhylidae) from the north coast ranges of Papua New Guinea. *Herpetologica*, 285-294.

Allison, A., and F. Kraus. (2003). A new species of *Austrochaperina* (Anura: Microhylidae) From northern Papua New Guinea. *Journal of Herpetology* 37: 637–644.

Almeida, A. D. P., & Angulo, A. (2006). A new species of *Leptodactylus* (Anura: Leptodactylidae) from the state of Espírito Santo, Brazil, with remarks on the systematics of associated populations. *Zootaxa*, *1334*, 1-25.

Alonso, R., & Rodríguez, A. (2003). Advertisement calls of Cuban toads of the genus *Bufo* (Anura, bufonidae). *Phyllomedusa: Journal of Herpetology*, *2*(2), 75–82. <https://doi.org/10.11606/issn.2316-9079.v2i2p75-82>

Alonso, R., Rodriguez, A., & Márquez, R. (2007). *Guia sonora de los anfibios de Cuba (Sound Guide of the Amphibians of Cuba)*. Barcelona: Alosa.

Amézquita, A., Castellanos, L., & Hödl, W. (2005). Auditory matching of male *Epipedobates femoralis* (Anura: dendrobatidae) under field conditions. *Animal Behaviour*, *70*(6), 1377–1386. <https://doi.org/10.1016/j.anbehav.2005.03.012>

Amezquita, A., Flechas, S. V., Lima, A. P., Gasser, H., & Hodl, W. (2011). Acoustic interference and recognition space within a complex assemblage of dendrobatid frogs. *Proceedings of the National Academy of Sciences*, *108*(41), 17058–17063. <https://doi.org/10.1073/pnas.1104773108>

Andrade, G. V., & Cardoso, A. J. (1986). Reconhecimento do grupo rizibilis; descrição de uma nova espécie de Hyla (Amphibia, Anura). *Revista Brasileira de Zoologia*, *3*(7), 433-440.

Andrade, S.P., Rocha, C.F., Victor-Jr., E.P. & Vaz-Silva, W. (2015) Advertisement call of *Rhinella inopina* Vaz-Silva, Valdujo & Pombal, 2012 (Anura: Bufonidae) from the type-locality, northeastern Goiás State, Central Brazil. Zootaxa, 3931 (3), 448–450. <http://dx.doi.org/10.11646/zootaxa.3931.3.10>

Andrade, F. S., & Carvalho, T. R. (2013). A new species of *Pseudopaludicola* Miranda-Ribeiro (Leiuperinae: Leptodactylidae: Anura) from the Cerrado of southeastern Brazil. *Zootaxa*, *3608*(5), 389-397.

Andreone, F., Vences, M., Guarino, F. M., Glaw, F., & Randrianirina, J. E. (2002). Natural history and larval morphology of Boophis occidentalis (Anura: Mantellidae: Boophinae) provide new insights into the phylogeny and adaptive radiation of endemic Malagasy frogs. *Journal of Zoology*, *257*(4), 425-438.

Andreone, F., Mattioli, F., & Mercurio, V. (2005). The call of Scaphiophryne gottlebei, a microhylid frog from the Isalo Massif, southcentral Madagascar. *Current Herpetology*, *24*(1), 33-35.

Andreone, F., Rosa, G. M., Noël, J., Crottini, A., Vences, M., & Raxworthy, C. J. (2010). Living within fallen palm leaves: the discovery of an unknown Blommersia (Mantellidae: Anura) reveals a new reproductive strategy in the amphibians of Madagascar. *Naturwissenschaften*, *97*(6), 525-543.

Angulo, A., Cocroft, R. B., & Reichle, S. (2003). Species identity in the genus Adenomera (Anura: Leptodactylidae) in southeastern Peru. *Herpetologica*, *59*(4), 490-504.

Angulo, A., & Reichle, S. (2008). Acoustic signals, species diagnosis, and species concepts: the case of a new cryptic species of *Leptodactylus* (Amphibia, Anura, Leptodactylidae) from the Chapare region, Bolivia. *Zoological Journal of the Linnean Society*, *152*(1), 59-77.

Angulo, A., & Icochea, J. (2010). Cryptic species complexes, widespread species and conservation: lessons from Amazonian frogs of the *Leptodactylus marmoratus* group (Anura: Leptodactylidae). *Systematics and Biodiversity*, *8*(3), 357-370.

Ao, J. M., Bordoloi, S., & Ohler, A. (2003). Amphibian fauna of Nagaland with nineteen new records from the state including five new records for India. *Zoos’ Print Journal*, *18*(6), 1117–1125. https://doi.org/10.11609/JoTT.ZPJ.18.6.1117-25

Aowphol, A., Rujirawan, A., Taksintum, W., Arsirapot, S., & Mcleod, D. S. (2013). Re-evaluating the taxonomic status of *Chiromantis* in Thailand using multiple lines of evidence (Amphibia: Anura: Rhacophoridae). *Zootaxa*, *3702*(2), 101–123. https://doi.org/10.11646/zootaxa.3702.2.1

Arak, A. (1983). Vocal interactions, call matching and territoriality in a Sri Lankan treefrog, *Philautus leucorhinus* (Rhacophoridae). *Animal Behaviour*, *31*(1), 292–302. https://doi.org/10.1016/S0003-3472(83)80199-7

Arak, A. (1988). Female mate selection in the natterjack toad: active choice or passive attraction? *Behavioral Ecology and Sociobiology*, *22*(5), 317–327. https://doi.org/10.1007/BF00295100

Araújo, C. B. D., Guerra, T. J., Amatuzzi, M. C. O., & Campos, L. A. (2012). Advertisement and territorial calls of Brachycephalus pitanga (Anura: brachycephalidae). *Zootaxa*, *3302*(1), 66–67.

Ávila, R. W., A. Pansonato, and C. Strüssmann. (2010). A new species of the *Rhinella margaritifera* group (Anura: Bufonidae) from Brazilian Pantanal. *Zootaxa* 2339: 57–89.

Ávila, R. W., Pansonato, A., & Strüssmann, C. (2012). A new species of Proceratophrys (Anura: Cycloramphidae) from midwestern Brazil. *Journal of Herpetology*, *46*(4), 466-472.

Ayarzagüena, J. and J. C. Señaris. (1994). Dos nuevas especies de *Hyla* (Anura; Hylidae) para las cumbres tepuyanas del Estado Amazonas, Venezuela. *Memoria de la Sociedad de Ciencias Naturales La Salle* 139:127–146.

Ayre, D. J., Coster, P., Bailey, W. J., & Roberts, J. D. (1984). Calling tactics in Crinia georgiana (Anura: Myobatrachidae): alternation and variation in call duration. *Australian journal of zoology*, *32*(4), 463-470.

Bain, R. H., Stuart, B. L., & Orlov, N. L. (2006). Three new Indochinese species of cascade frogs (Amphibia: Ranidae) allied to *Rana archotaphus*. *Copeia*, *2006*(1), 43–50.

Barker, J., Grigg, G., & Tyler, M. (1995). *A field guide to australian frogs*. (G. C. Grigg & M. J. Tyler, Eds.) (2nd edition). Chipping Norton, N.S.W: Surrey Beatty & Sons Pty Ltd.

Baldissera , F. A., Caramaschi, U., & Haddad, C. F. B. (2004). Review of the Bufo crucifer species group, with descriptions of two new related species (Amphibia, anura, bufonidae). *Arquivos Do Museu Nacional*, *62*(3), 255.

Baldo, D., & Basso, N. G. (2004). A new species of melanophryniscus gallardo, 1961 (Anura: bufonidae), with comments on the species of the genus reported for misiones, northeastern argentina. *Journal of Herpetology*, *38*(3), 393–403. <https://doi.org/10.1670/144-03A>

Baldo, D., Borteiro, C., Kolenc, F., Rosset, S., Prigioni, C., & Debat, C. M. (2012). The taxonomic status of Melanophryniscus orejasmirandai Prigioni & Langone, 1987 “1986” (Anura: bufonidae). *Zootaxa*, *3235*(1), 45–61.

Barrio, A. (1964). Especies crípticas del género Pleurodema que conviven en una misma área, identificadas por el canto nupcial (Anura, Leptodactylidae). *Physis*, *24*(68), 471-489.

Barrio, A. (1965). El género *Physalaemus* en la Argentina.(Anura, Leptodactylidae). *Physis*, *25*(70), 421-488.

Barrio-Amorós, C. L. (2006). A new species of *Phyllomedusa* (Anura: Hylidae: Phyllomedusinae) from northern Venezuela. *Zootaxa* 1309: 55–68.

Barrio-Amorós, C. L., A. Díaz De P., J. J. Mueses- Cisneros, E. Infante, and A. Chacón. (2006). Hyla vigilans Solano, 1971, a second species for the genus Scarthyla, redescription and distribution in Venezuela and Colombia. *Zootaxa* 1349: 1-18

Barrio-Amorós, C. L., & Santos, J. C. (2009). Description of a new Allobates (Anura, dendrobatidae) from the eastern Andean piedmont, Venezuela. *Phyllomedusa: Journal of Herpetology*, *8*(2), 89–104. <https://doi.org/10.11606/issn.2316-9079.v8i2p89-104>

Barrio-Amorós, C. L., Señaris, J. C., Macculloch, R. D., Lathrop, A., Guayasamin, J. M., & Duellman, W. E. (2011). Distribution, vocalization and taxonomic status of *Hypsiboas roraima* and *H. angelicus* (Amphibia: anura: hylidae). *Papéis Avulsos de Zoologia*, *51*(2), 21–28. https://doi.org/10.1590/S0031-10492011000200001

Barrio-Amorós, C. L., Heinicke, M. P., & Hedges, S. B. (2013). A new tuberculated Pristimantis (Anura, terrarana, strabomantidae) from the Venezuelan Andes, redescription of Pristimantis pleurostriatus , and variation within Pristimantis vanadisae. Zootaxa, 3647(1), 43–62. https://doi.org/10.11646/zootaxa.3647.1.2

Basso, N. G. (1990). Estrategias adaptivas en una comunidad subtropical de anuros. *Cuadernos de Herpetologia Serie Monografías*, 1, 1-70.

Bastos, R. P. And C. F. B. Haddad (1995). Vocalizações e interações acústicas de *Hyla elegans* (Anura, Hylidae) durante a atividade reprodutiva. *Naturalia* 20:165-176

Bastos, R. P. and J. P. Pombal Jr. (1995). New species of *Crossodactylus* (Anura: Leptodactylidae) from the Atlantic rain forest of southeastern Brazil. *Copeia* 1995:436–439

Bastos, R. P., Signorelli, L., Morais, A. R., Costa, T. B., Lima, L. P., & Pombal Jr, J. P. (2011). Advertisement calls of three anuran species (Amphibia) from the Cerrado, central Brazil. *South American Journal of Herpetology*, *6*(2), 67-72.

Bastos, R. P., M. A F. Bueno, S. L. Dutra, and L. P. Lima. (2003). Padrões de vocalização de anúncio em cinco espécies de Hylidae (Amphibia: Anura) do Brasil Central. *Comunicação do Museu de Ciências e Tecnologia da PUCRS, Série Zoologia* 16:139–51.

Batallas R., D., & Brito M., J. (2014). Descripción del llamado de advertencia de *Noblella lochites* (Anura: craugastoridae). *Avances En Ciencias e Ingeniería*, *6*(1). https://doi.org/10.18272/aci.v6i1.152

Batista, V. G., Ramalho, W. P., Amaral, D. F. D., Maciel, N. M., & Bastos, R. P. (2016). The advertisement and aggressive calls of Rhinella abei (Baldissera, caramaschi, and haddad, 2004) (Anura: bufonidae) from Campo Largo, Paraná, Brazil. *Zootaxa*, *4107*(4), 595–597. https://doi.org/10.11646/zootaxa.4107.4.7

Bee, M. A., Suyesh, R., & Biju, S. D. (2013). The vocal repertoire of Pseudophilautus kani , a shrub frog (Anura: rhacophoridae) from the Western Ghats of India. *Bioacoustics*, *22*(1), 67–85. https://doi.org/10.1080/09524622.2012.712750

Bennett, D., Hampson, K., Sanders, K., & Anderson, M. (2000). *Frogs of Coorg, Karnataka, India*. Aberdeen University, Viper press Great Britain. pp 139.

Bernal, S. H., Sontealegre, D. P., & Páez, C. A. (2004). Estudio de la vocalización de trece especies de anuros del municipio de Ibagué, Colombia. *Revistas de la Academia Colombiana de Ciencias Exactas, Físicas y Naturales*, *28*(108), 385–390.

Berneck, B. V. M., COSTA, C. O. R. D., & GARCIA, P. C. D. A. (2008). A new species of *Leptodactylus* (Anura: Leptodactylidae) from the Atlantic forest of São Paulo state, Brazil. *Zootaxa*, (1795), 46-56.

Berneck, B. V. M., Targino, M., & Garcia, P. C. D. A. (2013). Rediscovery and re-description of Ischnocnema nigriventris (Lutz, 1925) (Anura: terrarana: brachycephalidae). *Zootaxa*, *3694*(2), 131–142. <https://doi.org/10.11646/zootaxa.3694.2.2>

Biju, S. D., Van Bocxlaer, I., Giri, V. B., Roelants, K., Nagaraju, J., & Bossuyt, F. (2007). A new nightfrog, Nyctibatrachus minimus sp. nov. (Anura: Nyctibatrachidae): the smallest frog from India. *Current Science*, 854-858.

Biju, S. D., & Bossuyt, F. (2009). Systematics and phylogeny of Philautus Gistel, 1848 (Anura, rhacophoridae) in the Western Ghats of India, with descriptions of 12 new species. *Zoological Journal of the Linnean Society*, *155*(2), 374–444. <https://doi.org/10.1111/j.1096-3642.2008.00466.x>

Biju, S. D., Van Bocxlaer, I., Mahony, S., Dinesh, K. P., Radhakrishnan, C., Zachariah, A., Giri V & Bossuyt, F. (2011). A taxonomic review of the Night Frog genus Nyctibatrachus Boulenger, 1882 in the Western Ghats, India (Anura: Nyctibatrachidae) with description of twelve new species. *Zootaxa*, (3029), 1-96.

Bilate, M., & Lack, E. (2011). The advertisement call of Scinax similis (Cochran, 1952) (Amphibia, Anura). *South American Journal of Herpetology*, *6*(1), 54-58.

Blackburn, D. C., Kosuch, J., Schmitz, A., Burger, M., Wagner, P., Gonwouo, L. N., … Rödel, M.-O. (2008). A new species of cardioglossa (Anura: arthroleptidae) from the upper guinean forests of west africa. *Copeia*, *2008*(3), 603–612. <https://doi.org/10.1643/CH-06-233>

Boeckle, M., Preininger, D., & Hödl, W. (2009). Communication in noisy environments i: acoustic signals of staurois latopalmatus boulenger 1887. *Herpetologica*, *65*(2), 154–165. https://doi.org/10.1655/07-071R1.1

Boettger, O. (1885). Liste von reptilien und batrachiern aus Paraguay. *Zeitschrift für Naturwissenschaften*, *58*(3), 213-248. Available at: https://www.biodiversitylibrary.org/part/69906#/summary

Boistel, R., de Massary, J. C., & Angulo, A. (2006). Description of a new species of the genus *Adenomera* (Amphibia, Anura, Leptodactylidae) from French Guiana. *Acta Herpetologica*, *1*(1), 1-14.

Bokermann, W. C. A. (1963). Nova espécie de *Hyla* da Bahia, Brasil. *Atas da Sociedade de Biologia do Rio de Janeiro*, *7*, 6–8.

Bokermann, W. C. A. (1964a) *Dos nuevas especies de Hyla de Minas Gerais y notas sobre Hyla alvarengai Bok. (Amphibia, Salientia, Hylidae).* *Neotropica*, *10*, 67–76.

[Bokermann, W. C. A.](http://research.amnh.org/vz/herpetology/5_6amphibia/?action=names&a_id=3190) ([1964](http://research.amnh.org/vz/herpetology/5_6amphibia/?action=names&year=1964)b). Notes on treefrogs of the *Hyla marmorata* group with description of a new species (Amphibia, Hylidae). [*Senckenbergiana Biologica*](http://research.amnh.org/vz/herpetology/5_6amphibia/?action=names&p_id=297), 45, 243–254.

Bokermann, W. C. A. (1965). Três novos batraquios da região central de Mato Grosso, Brasil. Revista Brasileira de Biologia 25: 257–264.

Bokermann, W. C. A. (1967). Três novas espécies de *Physalaemus* do sudeste Brasileiro (Amphibia, Leptodactylidae). *Revista Brasileira de Biologia* 27:135–143.

Bokermann, W. C. A., and I. Sazima. (1973). Anfíbios da Serra do Cipó, Minas Gerais, Brasil. 1—Espécies novas de *Hyla* (Anura, Hylidae). *Revista Brasileira de Biologia* 33: 329–336.

Bosch, J., De la Riva, I., and Márquez, R. (1996). The calling behavior of *Lysapsus limellus* and *Pseudis paradoxa* (Amphibia:Anura:Pseudidae). *Folia Zoologica*, 45(1), 49-55.

Bosch, J., I. De la Riva, and R. Márquez. (2000). Advertisement calls of seven species of hyperoliid frogs from Equatorial Guinea. *Amphibia-Reptilia* 21:246–255.

Bossuyt, F., & Dubois, A. (2001). A review of the frog genus *Philautus* Gistel, 1848 (Amphibia, Anura, Ranidae, Rhacophorinae). *Zeylanica. Colombo*, *6*, 1–112.

Boulenger, G. A. (1900). Batrachians. Pages 55–56, pl. 5, in E. R. Lankester (communicator), Report on a collection made by Messrs. F. V. McConnell and J. J. Quelch at Mount Roraima in British Guiana. *Transactions of the Linnean Society of, 2nd series, Zoology*, *8*, 51–76.

Boulenger, G. A. (1900). A list of the batrachians and reptiles of the Gaboon (French Congo), with descriptions of new genera and species. *Proceedings of the Zoological Society of London* 1900: 433–456.

Boulenger, G. A. (1905). Xx. — *descriptions of new tailless batrachians in the collection of the british museum*. *Annals and Magazine of Natural History*, *16*(92), 180–184. <https://doi.org/10.1080/03745480509443666>

Boulenger, G. A. (1912). Descriptions of new African batrachians preserved in the British Museum. *Annals and Magazine of Natural History*, *8*(10), 140–142.

Brandão, R. A. (2002). A new species of *Phyllomedusa* Wagler, 1830 (Anura: Hylidae) from central Brazil. *Journal of Herpetology* 36: 571–578.

Brandão, R. A., Álvares, G. F., Crema, A., & Zerbini, G. J. (2009). Natural History of Phyllomedusa centralis (Anura: Hylidae: Phyllomedusinae): Tadpole and Calls. *South American Journal of Herpetology*, *4*(1), 61-68.

Brandão, R. A., and G. F. R. Álvarez. (2009). Remarks on "A new *Phyllomedusa* Wagler (Anura, Hylidae) with reticulated pattern on flanks from southeastern Brazil". *Zootaxa* 2044: 61–64.

Brandão, R.A., Magalhães, R.F., Garda, A.A., Campos, L.A., Sebben, A. & Maciel, N.M. (2012) A new species of *Bokermannohyla* (Anura: Hylidae) from highlands of Central Brasil. *Zootaxa*, 3527, 28–42.

Brasileiro, C. A., Haddad, C. F. B., Sawaya, R. J., & Sazima, I. (2007a). A new and threatened island-dwelling species of Cycloramphus (Anura: cycloramphidae) from southeastern brazil. *Herpetologica*, *63*(4), 501–510. [https://doi.org/10.1655/0018-0831(2007)63[501:ANATIS]2.0.CO;2](about:blank)

Brasileiro, C. A., Haddad, C. F., Sawaya, R. J., & Martins, M. (2007b). A new and threatened species of Scinax (Anura: Hylidae) from Queimada Grande Island, southeastern Brazil. *Zootaxa*, *1391*(1), 47-55.

Briggs, V. S. (2010). Call trait variation in morelett’s tree frog, agalychnis moreletii, of belize. *Herpetologica*, *66*(3), 241–249. https://doi.org/10.1655/HERPETOLOGICA-D-09-00011.1

Brown, J. L., Twomey, E., Pepper, M., & Rodriguez, M. S. (2008). Revision of the *Ranitomeya fantastica* species complex with description of two new species from Central Peru (Anura: dendrobatidae). *Zootaxa*, *1823*(1), 1–24.

Brown, J. L., & Twomey, E. (2009). Complicated histories: three new species of poison frogs of the genus *Ameerega* (Anura: dendrobatidae) from north-central Peru. *Zootaxa*, *2049*(1), 1–38.

Brown, R. M., & Iskandar, D. T. (2000). Nest site selection, larval hatching, and advertisement calls, of *Rana arathooni* from southwestern sulawesi (Celebes) island, indonesia. *Journal of Herpetology*, *34*(3), 404–413. <https://doi.org/10.2307/1565364>

Brown, R. M., Siler, C. D., Diesmos, A. C., & Alcala, A. C. (2009). Philippine frogs of the genus Leptobrachium (Anura; Megophryidae): phylogeny-based species delimitation, taxonomic review, and descriptions of three new species. *Herpetological Monographs*, *23*(1), 1-44.

Budgett, J. S. (1899). Notes on the batrachians of Paraguayan Chaco, with observations upon their breeding habits and development, especially with regard to *Phyllomedusa hypochondrialis* Cope. Also a description of a new genus. *Quarterly Journal of Microscopical Science. London*, *42*, 305–333.

Byrne, P. G. (2008). Strategic male calling behavior in an Australian terrestrial toadlet (Pseudophryne bibronii). *Copeia*, 57-63.

Cadle, J. E., & McDiarmid, R. W. (1990). Two new species of Centrolenella (Anura: centrolenidae) from northwestern Peru. *Proceedings of the Biological Society of Washington*, *103*(3), 23.

Cajade, R., Schaefer, E. F., Duré, M. I., Kehr, A. I., & Marangoni, F. (2010). Reproductive biology of *Argenteohyla siemersi pederseni* Williams and Bosso, 1994 (Anura: hylidae) in northeastern Argentina. *Journal of Natural History*, *44*(31–32), 1953–1978. <https://doi.org/10.1080/00222931003642590>

Caldart, V. M., Iop, S., & Cechin, S. Z. (2011). Vocalizations of *Crossodactylus schmidti* Gallardo, 1961 (Anura, Hylodidae): advertisement call and aggressive call. *North-Western Journal of Zoology*, *7*(1).

Caldart, V. M., Santos, T. G. dos, & Maneyro, R. (2013). The advertisement and release calls of *Melanophryniscus pachyrhynus* (Miranda-Ribeiro, 1920) from the central region of Rio Grande do Sul, southern Brazil. *Acta Herpetologica*, *8*(2), 115–122. https://doi.org/10.13128/Acta_Herpetol-12680

Caldwell, J.P. , & Hoogmoed, M. S. (1998). Allophrynidae, Allophryne, A. ruthveni. In *Catalogue of American Amphibians and Reptiles.*  (Vol. 666, p. 1–3). Society for the Study of Amphibians and Reptiles. Recuperado de <https://repositories.lib.utexas.edu/handle/2152/44676>

Caldwell, J. P., Lima, A. P., & Keller, C. (2002). Redescription of colostethus marchesianus (Melin, 1941) from its type locality. *Copeia*, *2002*(1), 157–165. [https://doi.org/10.1643/0045-8511(2002)002[0157:ROCMMF]2.0.CO;2](about:blank)

Caldwell, J. P., & Lima, A. P. (2003). A new amazonian species of *Colostethus* (Anura: dendrobatidae) with a nidicolous tadpole. *Herpetologica*, *59*(2), 219–234. https://doi.org/10.1655/0018-0831(2003)059[0219:ANASOC]2.0.CO;2

Caldwell, J. P., & Shepard, D. B. (2007). Calling site fidelity and call structure of a neotropical toad, *Rhinella ocellata* (Anura: bufonidae). *Journal of Herpetology*, *41*(4), 611–621. <https://doi.org/10.1670/07-025.1>

Caminer, M., & Ron, S. (2014). Systematics of treefrogs of the Hypsiboas calcaratus and Hypsiboas fasciatus species complex (Anura, hylidae) with the description of four new species. *ZooKeys*, *370*, 1–68. <https://doi.org/10.3897/zookeys.370.6291>

Campbell, J. A., & Clarke, B. T. (1998). A review of frogs of the genus *Otophryne* (Microhylidae) with the description of a new species. *Herpetologica*, *54*(3), 301–317.

Campos, T. F., De Lima, M. G., Do Nascimento, F. A. C., & Dos Santos, E. M. (2014). Larval morphology and advertisement call of Phyllodytes acuminatus Bokermann, 1966 (Anura: Hylidae) from Northeastern Brazil. *Zootaxa*, *3779*(1), 93-100.

Camurugi, F., Röhr, D. L., & Juncá, F. A. (2015). Differences in advertisement calls and vocal behavior in hypsiboas atlanticus (Anura: hylidae) among microhabitats. *Herpetologica*, *71*(4), 243–251. <https://doi.org/10.1655/HERPETOLOGICA-D-14-00070>

Canedo, C., and J. P. Pombal, Jr. (2007). Two new species of torrent frog of the genus *Hylodes* (Anura, Hylodidae) with nuptial thumb tubercles. *Herpetologica* 63: 224–235.

Cannatella, D. C. (1980). Two new species of Centrolenella from bolivia anura centrolenidae. *Proceedings of the Biological Society of Washington.*, *93*, 714–724.

Cannatella, D. C. (1980b). A review of the *Phyllomedusa buckleyi* group (Anura: Hylidae). *Occasional Papers of the Museum of Natural History of the University of Kansas* 87:1–40.

Cannatella, D. C., and W. E. Duellman. (1984). Leptodactylid frogs of the *Physalaemus pustulosus* group. Copeia 1984: 902–921.

Capula, M., & Carti, M. (1993). Morphometric variation and divergence in the west mediterranean discoglossus(Amphibia: discoglossidae). *Journal of Zoology*, *231*(1), 141–156. <https://doi.org/10.1111/j.1469-7998.1993.tb05358.x>

Caramaschi, U. (1996) Nova espécie de *Odontophrynus* Reinhardt & Lütken, 1862 do Brasil Central (Amphibia, Anura, Leptodactylidae). *Boletim do Museu Nacional, Nova Série, Zoologia*, 367, 1–8.

Caramaschi, U. (2006). Redefinição do grupo de *Phyllomedusa hypochondrialis*, com redescrição de *P. megacephala* (Miranda-Ribeiro, 1926), revalidação de *P. azurea* Cope, 1862 e descrição de uma nova espécie (Amphibia, Anura, Hylidae). *Arquivos do Museu Nacional. Rio de Janeiro* 64: 159–179.

Caramaschi, U., and J. Jim. (1983). Uma nova espécie de *Hyla* do grupo *marmorata* do nordeste Brasileiro (Amphibia, Anura, Hylidae). *Revista Brasileira de Biologia* 43: 195–198.

Caramaschi, U., & Feio, R. N. (1990). A new species of Hyla (Anura, hylidae) from southern minas gerais, Brazil. *Copeia*, *1990*(2), 542–546. <https://doi.org/10.2307/1446357>

Caramaschi, U. and C. A. G. Cruz. (1997). Redescription of *Chiasmocleis albopunctata* (Boettger) and description of a new species of *Chiasmocleis* (Anura: Microhylidae). *Herpetologica* 53:259–268.

Caramaschi, U., and C. A. G. Cruz. (2000). Duas espécies novas de *Hyla* Laurenti, 1768 do Estado de Goiás, Brasil (Amphibia, Anura, Hylidae). Boletim do Museu Nacional. Nova Serie, Zoologia. Rio de Janeiro 422: 1–12.

Caramaschi, U., Niemeyer, H. (2003): New species of the Hyla albopunctata group from central Brazil (Amphibia, Anura, Hylidae). Boletim do Museu Nacional, Nova Série 504: 1-­8.

Caramaschi, U., & Pimenta, B. V. S. (2003). Duas novas espécies de Chiasmocleis Méhelÿ, 1904 da Mata Atlântica do Sul da Bahia, Brasil (Amphibia, Anura, Microhylidae). *Arquivos do Museu Nacional*, *61*(3), 195-202.

Caramaschi, U., and C. A. G. Cruz. (2004). Duas novas espécies de *Hyla* do grupo de *H. polytaenia* Cope, 1870 do sudeste do Brasil (Amphibia, Anura, Hylidae). Arquivos do Museu Nacional. Rio de Janeiro 62: 247–254.

Caramaschi, U., B. V. S. Pimenta, and R. N. Feio. (2004). Nova espécie do grupo de *Hyla geographica* Spix, 1824 da floresta Atlântica, Brasil (Amphibia, Anura, Hylidae). Boletim do Museu Nacional. Nova Serie, Zoologia. Rio de Janeiro 518: 1–14.

Caramaschi, U., Feio, R. N., & Sao-Pedro, V. A. (2008). A new species of *Leptodactylus* Fitzinger (Anura, Leptodactylidae) from Serra do Brigadeiro, State of Minas Gerais, Southeastern Brazil. *Zootaxa*, *1861*, 44-54.

Caramaschi, U., C. A. G. Cruz, and L. B. Nascimento. (2009). A new species of *Hypsiboas* of the *H. polytaenius* clade from southeastern Brazil (Anura: Hylidae). South American Journal of Herpetology 4: 210–216.

Caramaschi, U., & Napoli, M. F. (2012). Taxonomic revision of the *Odontophrynus cultripes* species group, with description of a new related species (Anura, Cycloramphidae). *Zootaxa*, *3155*, 1-20.

Caramaschi, U., Orrico, V. G. D., Faivovich, J., Dias, I. R., & Solé, M. (2013). A new species of *Allophryne* (Anura: allophrynidae) from the atlantic rain forest biome of eastern brazil. *Herpetologica*, *69*(4), 480–491. <https://doi.org/10.1655/HERPETOLOGICA-D-13-00029>

Cardoso, A. J. (1983). Descrição e biologia de uma nova espécie de *Hyla* Laurenti, 1768 (Amphibia, Anura, Hylidae). Iheringia. Série Zoologia 62:37–45.

Cardoso, A. J., & Sazima, I. (1980). Nova espécie de Hyla do sudeste brasileiro (Amphibia, Anura, Hylidae). *Revista Brasileira de Biologia*, *40*(1), 75-79.

Cardoso, A. J., and G. V. de Andrade. (1982). Nova espécie de *Hyla* do Parque Nacional Serra da Canastra (Anura, Hylidae). Revista Brasileira de Biologia 42: 589–593.

Cardoso, A. J. and Heyer, W. R. (1995). Advertisement, aggressive, and possible seismic signals of the frog *Leptodactylus syphax* (Amphibia, Leptodactylidae) . *Alytes*, 13(2): 67-76.

Cardozo, A.U., & Señaris, J. C. (2012). Vocalización y biología reproductiva de las ranas de cristal Hyalinobatrachium pallidum y Centrolene daidaleum (Anura, centrolenidae) en la sierra de Perijá, Venezuela. *Memoria de La Fundación La Salle de Ciencias Naturales*, (173–174), 87–105.

Carvalho, T. D. (2012). A new species of *Pseudopaludicola* Miranda-Ribeiro (Leiuperinae: Leptodactylidae: Anura) from the Cerrado of southeastern Brazil with a distinctive advertisement call pattern. *Zootaxa*, *3328*, 47-54.

Carvalho, T. R., A. A. Giaretta, and K. G. Facure. (2010). A new species of *Hypsiboas* Wagler (Anura: Hylidae) closely related to *H. multifasciatus* Günther from southeastern Brazil. *Zootaxa*, 2521:37–52.

Carvalho, T.R., & Ron, S. (2011). Advertisement call of Leptodactylus labrosus Jiménez de la Espada, 1875 (Anura, Leptodactylidae): an unusual advertisement call within the L. fuscus group. *Herpetology Notes*, *4*, 325-326.

Carvalho, T.R. & Martins, L.B. (2012) Advertisement call of *Haddadus binotatus* (Spix, 1824) (Anura: Terrarana: Craugastoridae) from three localities in the State of Rio de Janeiro, with comments on its bioacoustic variability. *Herpetology Notes*, 5, 419–422.

Carvalho, T. R. de, Giaretta, A. A., & Magrini, L. (2012). A new species of the Bokermannohyla circumdata group (Anura: hylidae) from southeastern Brazil, with bioacoustic data on seven species of the genus. *Zootaxa*, (3321), 37–55.

Carvalho, T. R., & Giaretta, A. A. (2013a). Bioacoustics reveals two new syntopic species of Adenomera Steindachner (Anura: Leptodactylidae: Leptodactylinae) in the Cerrado of central Brazil. *Zootaxa*, *3731*(3), 533-551.

Carvalho, T. R., & Giaretta, A. A. (2013b). Taxonomic circumscription of Adenomera martinezi (Bokermann, 1956)(Anura: Leptodactylidae: Leptodactylinae) with the recognition of a new cryptic taxon through a bioacoustic approach. *Zootaxa*, *3701*(2), 207-237

Carvalho, T., Tolentino, V., & Giaretta, A. (2013a). Advertisement call of Rhinella pygmaea (Myers and Carvalho, 1952) (Anura: Bufonidae) from the northern State of Rio de Janeiro. *Herpetology Notes* , *6*, 229–231.

Carvalho, T. D., Leite, F. S. F., & Pezzuti, T. L. (2013b). A new species of Leptodactylus Fitzinger (Anura, Leptodactylidae, Leptodactylinae) from montane rock fields of the Chapada Diamantina, northeastern Brazil. *Zootaxa*, *3701*(3), 349-364..

Carvalho, T. R. de, Martins, L. B., & Giaretta, A. A. (2016). A new account for the endangered Cerrado Rocket Frog Allobates goianus (Bokermann, 1975) (Anura: aromobatidae), with comments on taxonomy and conservation. *Acta Herpetologica*, *11*(1), 21–30. <https://doi.org/10.13128/Acta_Herpetol-17491>

Carvalho-e-Silva, S.P., Pinto, A.L.C. & Carvalho-e-Silva, A.M.P.T. (2002) Aspectos da reprodução, da vocalização e da larva *Phrynohyas mesophaea* Hensel (Amphibia, Anura, Hylidae). *Revista Aquarium*, 35, 19–24.

Cassini, C. S., C. A. G. Cruz, and U. Caramaschi. (2010). Taxonomic review of *Physalaemus olfersii* (Lichtenstein & Martens, 1856) with revalidation of *Physalaemus lateristriga* (Steindachner, 1864) and description of two new related species (Anura: Leiuperidae). *Zootaxa* 2491: 1–33.

Cassini, C. S., Orrico, V. G., Dias, I. R., Sole, M., & Haddad, C. F. (2013). Phenotypic variation of *Leptodactylus cupreus* Caramaschi, São-Pedro and Feio, 2008 (Anura, Leptodactylidae). *Zootaxa*, *3616*(1), 073-084.

Castanho, L. M., & Haddad, C. F. B. (2000). New species of *eleutherodactylus* (Amphibia: leptodactylidae) from guaraqueçaba, atlantic forest of brazil. *Copeia*, *2000*(3), 777–781. [https://doi.org/10.1643/0045-8511(2000)000[0777:NSOEAL]2.0.CO;2](about:blank)

Castellano, S., Cuatto, B., Rinella, R., Rosso, A., & Giacoma, C. (2002). The advertisement call of the european treefrogs (Hyla arborea): a multilevel study of variation. *Ethology*, *108*(1), 75–89. https://doi.org/10.1046/j.1439-0310.2002.00761.x

Castroviejo-Fisher, S., Ayarzagüena, J., & Vilà, C. (2007). A new species of hyalinobatrachium (Centrolenidae: anura) from serranía de perijá, venezuela. *Zootaxa*, *1441*(1), 51–62. https://doi.org/10.11646/zootaxa.1441.1.4

Castroviejo-Fisher, S., Señaris, J. C., Ayarzagüena, J., & Vilà, C. (2008). Resurrection of hyalinobatrachium orocostale and notes on the hyalinobatrachium orientale species complex(Anura: centrolenidae). *Herpetologica*, *64*(4), 472–484. https://doi.org/10.1655/07-049R2.1

Castroviejo-Fisher, S., Padial, J. M., Chaparro, J. C., Aguayo, R., & de la Riva, I. (2009). A new species of Hyalinobatrachium (Anura: centrolenidae) from the Amazonian slopes of the central Andes with comments on the diversity of the genus in the area. *Zootaxa*, (2143), 24–44.

Castroviejo-Fisher, S., Vilà, C., Ayarzagüena, J., Blanc, M. and Ernst, R., 2011. Species diversity of glassfrogs (Amphibia: Centrolenidae) from the Guiana Shield, with the description of two new species. *Hyalinobatrachium.* Zootaxa, vol. 3132, p. 1-55.

Catenazzi, A. and E. Lehr. 2009. The generic allocation of "*Hyla*" *antoniiochoai* De la Riva & Chaparro, 2005 (Anura), with description of its advertisement call and ecology. [*Zootaxa* 2304: 61-68](https://sites.google.com/site/acatenazzi/CatenazziLehr2009.pdf?attredirects=0)

Catenazzi, A. and R. von May. 2011. A new species of marsupial frog (Hemiphractidae: *Gastrotheca*) from an isolated montane forest in Peru. [*Journal of Herpetology* 45: 161-166](http://www.bioone.org/doi/abs/10.1670/10-070.1).

Catenazzi, A., Rodríguez, L. O., & Donnelly, M. A. (2009). The advertisement calls of four species of glassfrogs (Centrolenidae) from southeastern Peru. *Studies on Neotropical Fauna and Environment*, *44*(2), 83–91. <https://doi.org/10.1080/01650520903036653>

Catullo, R. A., Doughty, P., & Keogh, J. S. (2014). A new frog species (Myobatrachidae: Uperoleia) from the Northern Deserts region of Australia, with a redescription of U. trachyderma. *Zootaxa*, *3753*(3), 251-262.

Channing, A. (2001). *Amphibians of central and southern africa*. Ithaca, NY: Cornell University Press.

Channing, A., Hendricks, D., & Dawood, A. (1994). Description of a new moss frog from the south-western Cape (Anura: Ranidae: Arthroleptella). *South African Journal of Zoology*, *29*(4), 240–243. https://doi.org/10.1080/02541858.1994.11448357

Channing, A., & Bogart, J. P. (1996). Description of a tetraploid Tomopterna (Anura: ranidae) from South Africa. *South African Journal of Zoology*, *31*(2), 80–85. https://doi.org/10.1080/02541858.1996.11448397

Channing, A., Moyer, D. C., & Dawood, A. (2004). A new sand frog from central Tanzania (Anura: Ranidae: *Tomopterna*). *African Journal of Herpetology*, *53*(1), 21–28. https://doi.org/10.1080/21564574.2004.9635495

Channing, A., Menegon, M., Salvidio, S., & Akker, S. (2005). A new forest toad from the Ukaguru Mountains, Tanzania (Bufonidae : *Nectophrynoides*). *African Journal of Herpetology*, *54*(2), 149–157.

Channing, A., Brun, C., Burger, M., Febvre, S., & Moyer, D. (2005b). A new cryptic dainty frog from east africa(Anura: Ranidae: Cacosternum ). *African Journal of Herpetology*, *54*(2), 139–148. https://doi.org/10.1080/21564574.2005.9635527

Channing, A., Finlow-Bates, K. S., Haarklau, S. E., & Hawkes, P. G. (2006). The biology and recent history of the critically endangered kihansi spray toad nectophrynoides asperginis in tanzania. *Journal of East African Natural History*, *95*(2), 117–138. https://doi.org/10.2982/0012-8317(2006)95[117:TBARHO]2.0.CO;2

Channing, A., & Howell, K. (2006). *Amphibians of east africa* (1 edition). Ithaca: Comstock Publishing Associates.

Channing, A., & Schmitz, A. (2008). Hiding in plain sight: Another cryptic dainty frog from the highlands of Kenya (Anura: Pyxicephalidae: *Cacosternum*). *African Journal of Herpetology*, *57*(2), 75–84. https://doi.org/10.1080/21564574.2008.9635570

Channing, A., & Baptista, N. (2013). *Amietia angolensis* and *A*. *fuscigula* (Anura: Pyxicephalidae) in southern Africa: A cold case reheated. *Zootaxa*, *3640*, 501–520.

Channing, A., Schmitz, A., Burger, M., & Kielgast, J. (2013). A molecular phylogeny of African Dainty Frogs, with the description of four new species (Anura: pyxicephalidae: cacosternum). *Zootaxa*, *3701*(5), 518–550. https://doi.org/10.11646/zootaxa.3701.5.2

Chaves G., García-Rodríguez A., Mora A., Leal A. (2009). A new species of dink frog (Anura: Eleutherodactylidae: *Diasporus*) from Cordillera de Talamanca, Costa Rica. *Zootaxa* 2088:1–14.

Cherry, M. I., & Francillon-Vieillot, H. (1992). Body size, age and reproduction in the leopard toad, *Bufo pardalis*. *Journal of Zoology*, *228*(1), 41–50. https://doi.org/10.1111/j.1469-7998.1992.tb04431.x

Cherry, M. I., & Grant, W. S. (1994). Phylogenetic relationships and call structure in four African bufonid species. *African Zoology*, *29*(1), 1–10.

Chuaynkern, Y., Ohler, A., Inthara, C., Duengkae, P., Makchai, S., & Salangsingha, N. (2010). A revision of species in the subgenus *Nidirana* Dubois, 1992, with special attention to the identity of specimens allocated to *Rana adenopleura* Boulenger, 1909, and *Rana chapaensis* (Bourret, 1937) (Amphibia: Anura: Ranidae) from Thailand and Laos. *Raffles Bulletin of Zoology. Singapore*, *58*, 291–310.

Clarke, J. M. (2006). *Habitat, microhabitat and calling behaviour of Taudactylus pleione Czechura (Anura: Myobatrachidae), a critically endangered frog from central Queensland, Australia*. Master's Thesis. Faculty of Sciences, Engineering & Health, Central Queensland University.

Clulow, S., Anstis, M., Keogh, J. S., & Catullo, R. A. (2016). A new species of Australian frog (Myobatrachidae: Uperoleia) from the New South Wales mid-north coast sandplains. *Zootaxa*, *4184*(2), 285-3

Cochran, D. M. (1941). *The herpetology of Hispaniola.* Bulletin of the United States National Museum, (177), i-398. https://doi.org/10.5479/si.03629236.177.i

Cochran, D. M., & Goin, C. J. (1959). A new frog of the genus *Limnomedusa* from Colombia. *Copeia*, *1959*(3), 208-210.

Cochran, D. M., & Goin, C. J. (1970). Frogs of columbia. *Bulletin - United States National Museum.*, *288*, 1–655. https://doi.org/10.5962/bhl.part.6346

Cocroft, R. B., McDiarmid, R. W., Jaslow, A. P., & Ruiz-Carranza, P. M. (1990). Vocalizations of eight species of atelopus (Anura: bufonidae) with comments on communication in the genus. *Copeia*, *1990*(3), 631–643. https://doi.org/10.2307/1446428

Cocroft, R. B., & Ryan, M. J. (1995). Patterns of advertisement call evolution in toads and chorus frogs. *Animal Behaviour*, *49*(2), 283–303. <https://doi.org/10.1006/anbe.1995.0043>

Colli, G., Reichle, S., Silvano, D., Faivovich, J. (2004). Dermatonotus muelleri. The IUCN Red List of Threatened Species. Version 2014.2. Available at: http://www.iucnredlist.org.

Coloma, L. A. (1995). Ecuadorian frogs of the genus *Colostethus* (Anura: dendrobatidae) /. *Miscellaneous publication (University of Kansas. Natural History Museum)*, no. *87*. https://doi.org/10.5962/bhl.title.16171

Coloma, L. A., Lötters, S., & Salas, A. W. (2000). Taxonomy of the *Atelopus ignescens* complex (Anura: Bufonidae): designation of a neotype of *Atelopus ignescens* and recognition of *Atelopus exiguus*. *Herpetologica*, *56*(3), 303–324.

Coloma, L.A.; Carvajal-endara, S.; Dueñas, J.F.; Paredes-recalde, A.; Morales-mite, M.; Almeida-reinoso, D.; Tapia, E.E.; Hutter, C.R.; Toral, E. & Guayasamin, J.M. (2012). Molecular Phylogenetics of stream treefrogs of the *Hyloscirtus larinopygion* group (Anura: Hylidae), and description of two new species from Ecuador. *Zootaxa,* 3364:1-78.

Conradie, W., Branch, W. R., Measey, G. J., & Tolley, K. A. (2012). A new species of *Hyperolius* Rapp, 1842 (Anura: Hyperoliidae) from the Serra da Chela mountains, south-western Angola. *Zootaxa*, *3269*(1), 1-17.

Conradie, W., Branch, W. R., & Tolley, K. A. (2013). Fifty shades of grey: giving colour to the poorly known Angolan Ashy reed frog (Hyperoliidae: *Hyperolius cinereus*), with the description of a new species. *Zootaxa*, *3635*(3), 201-223.

Conte, C. E., Nomura, F., Machado, R. A., Kwet, A., Lingnau, R., & Rossa-Feres, D. de C. (2010). Novos registros na distribuição geográfica de anuros na floresta com araucária e considerações sobre suas vocalizações. *Biota Neotropica*, *10*(2), 201–224. <https://doi.org/10.1590/S1676-06032010000200024>

Costa-Campos, C. E., Lima, A. P., & Amézquita, A. (2016). The advertisement call of *Ameerega pulchripecta* (Silverstone, 1976) (Anura, dendrobatidae). *Zootaxa*, *4136*(2), 387–389. <https://doi.org/10.11646/zootaxa.4136.2.9>

Cruz, C.A.G.; Caramaschi, U. & Izecksohn, E. (1997). The genus *Chiasmocleis* Méhelÿ, 1904 (Anura, Microhylidae) in the Atlantic Rain Forest of Brazil, with description of three new species. Alytes, **15**(2):49-71.

Cruz, C. A. G., and B. V. S. Pimenta. (2004). New species of *Physalaemus* Fitzinger, 1826 from southern Bahia, Brazil (Anura, Leptodactylidae). *Journal of Herpetology* 38: 480–486.

Cruz, C. A. G., Prado, G. M., & Izecksohn, E. (2005). Nova espécie de *Proceratophrys* Miranda-Ribeiro, 1920 do sudeste do Brasil (Amphibia, Anura, Leptodactylidae). *Arquivos do Museu Nacional*, *63*(2), 289-295.

Cruz CAG, Nascimento LB, Feio RN. (2007) A new species of the genus *Physalaemus* Fitzinger, 1826 (Anuram Leiuperidae) from southeastern Brazil. *Amphibia-Reptilia* 28:457-465.

Cruz, C.A.G., Feio, R.N., & Nascimento, L.B. (2008a) A new species of *Phasmahyla* Cruz, 1990 (Anura: Hylidae) from the Atlantic Rain Forest of the States of Minas Gerais and Bahia, Brazil. *Amphibia-Reptilia,* 29, 311–318.

Cruz, C.A.G., Napoli, M.F., & Fonseca, P.M. (2008b) A new species of *Phasmahyla* Cruz, 1990 (Anura: Hylidae) from the state of Bahia, Brazil. *South American Journal of Herpetology,* 3, 187–195.

Cruz, C. A. G., & Napoli, M. F. (2010). A new species of smooth horned frog, genus *Proceratophrys* Miranda-Ribeiro (Amphibia: Anura: Cycloramphidae), from the Atlantic Rainforest of eastern Bahia, Brazil. *Zootaxa*, *2660*, 57-67.

Cruz, C. A. G., Nunes, I., & Juncá, F. A. (2012). Redescription of Proceratophrys cristiceps (Müller, 1883)(Amphibia, Anura, Odontophrynidae), with description of two new species without eyelid appendages from Northeastern Brazil. *south american Journal of herpetology*, *7*(2), 110-122.

Cuevas-Palma, C. C. (2013). Análisis taxonómico de Alsodes nodosus (Duméril & Bibron, 1841) (Amphibia, neobatrachia): antecedentes morfológicos y moleculares. Recuperado de <http://repositorio.conicyt.cl/handle/10533/180449>

Clulow, S., Mahony, M., Elliott, L., Humfeld, S., & Gerhardt, H. C. (2017). Near-synchronous calling in the hip-pocket frog Assa darlingtoni. *Bioacoustics*, *26*(3), 249-258.

Daniel, J. C. (2002). *The Book of Indian Reptiles and Amphibians.* Bombay National History Society, Delhi.

Das, I. (2000). Nomenclatural history and rediscovery of *Rhacophorus lateralis* Boulenger, 1883(Amphibia: Rhacophoridae). *Current Herpetology*, *19*(1), 35–40. https://doi.org/10.5358/hsj.19.35

Das, I., & Ravichandran, M. S. (1998). A new species of polypedates (Anura: rhacophoridae) from the western ghats, india, allied to the sri lankan p. Cruciger blyth, 1852. *Hamadryad*, *22*, 88–94.

Davies, M., Martin, A.A., & Watson, G.F. (1983) Redefinition of the Litoria latopalmata species group (Anura: Hylidae). Transactions of the Royal Society of South Australia, 107, 87–108.

Davies, M. M., M. J. Mahony, and J. D. Roberts. (1985). A new species of *Uperoleia* (Anura: Leptodactylidae) from the Pilbara Region, Western Australia. *Transactions of the Royal Society of South Australia* 109: 103–108.

Davies, M., and M.J. Littlejohn (1986) The frog genus *Uperoleia* Gray (Anura: Leptodactylidae) in south eastern Australia. *Transactions of the Royal Society of South Australia* 11O:lll- 143.

Davies, M., & Watson, G. F. (1994). Morphology and reproductive biology of-Limnodynastes salmini, L. convexiusculus and Megistolotis lignarius (Anura: Leptodactylidae: Limnodynastinae). *Transactions of the Royal Society of South Australia*, *118*(3), 149-169.

Dawood, A., & Channing, A. (2002). Description of a new cryptic species of African sand frog, Tomopterna damarensis (Anura: Ranidae), from Namibia. *African Journal of Herpetology*, *51*(2), 129–134. https://doi.org/10.1080/21564574.2002.9635468

D'cruze, N., Köhler, J., Vences, M., & Glaw, F. (2010). A new fat fossorial frog (Microhylidae: Cophylinae: Rhombophryne) from the rainforest of the Forêt d'Ambre Special Reserve, Northern Madagascar. *Herpetologica*, *66*(2), 182-191.

De la Riva, I. (1993a). A new species of Scinax (Anura, Hylidae) from Argentina and Bolivia. *Journal of Herpetology*, 41-46.

De la Riva, I. (1993b): Ecología de una comunidad Neotropical de an. bios durante la estación lluviosa. Unpublished PhD Disseration, Universidad Complutense, Madrid.

De la Riva, I. (1999). A new *Phyllomedusa* from southwestern Amazonia (Amphibia: Anura: Hylidae). *Revista Española de Herpetología*, *13*, 123–131.

De la Riva, I. (2007). Bolivian frogs of the genus *Phrynopus*, with the description of twelve new species (Anura: Brachycephalidae). *Herpetological Monographs*, *21*, 241–277.

De la Riva, I., Márquez, R., & Bosch, J. (1994). Advertisement calls of Bolivian species of *Scinax* (Amphibia, anura, Hylidae). *Bijdragen tot de Dierkunde*, *64*(2), 75-85.

De la Riva, I., Márquez, R., & Bosch, J. (1995). Advertisement calls of eight Bolivian hylids (Amphibia, Anura). *Journal of herpetology*, *29*(1), 113-118.

De la Riva, I., Márquez, R., & Bosch, J. (1996a). The advertisement calls of three South American poison frogs (Amphibia: anura: dendrobatidae), with comments on their taxonomy and distribution. *Journal of Natural History*, *30*(9), 1413–1420. <https://doi.org/10.1080/00222939600771311>

De la Riva, I., Marquez, R., & Bosch, J. (1996b). Advertisement calls of four microhylid frogs from bolivia (Amphibia, anura). *The American Midland Naturalist*, *136*(2), 418–422. <https://doi.org/10.2307/2426746>

De la Riva, I., & Lynch, J. D. (1997). New species of eleutherodactylus from bolivia(Amphibia: leptodactylidae). Copeia, 1997(1), 151–157. https://doi.org/10.2307/1447850

De la Riva, I., Márquez, R., & Bosch, J. (1997). Description of the advertisement calls of some south american hylidae) amphibia, anura): taxonomic and methodological consequences. *Bonner zoologische Beiträge : Herausgeber: Zoologisches Forschungsinstitut und Museum Alexander Koenig, Bonn.*, *47*, 175–185.

De la Riva, I., & Köhler, J. (1998). A new minute leptodactylid frog, genus phyllonastes, from humid montane forests of bolivia. *Journal of Herpetology*, *32*(3), 325–329. https://doi.org/10.2307/1565445

De la Riva, I., Bosch, J., & Márquez, R. (2004). The advertisement calls of two New Guinean species of Litoria (Amphibia, anura, hylidae). *Amphibia-Reptilia*, *25*(2), 173–178. https://doi.org/10.1163/1568538041231175

Decicco, L. H., & Holman, W. G. (2017). *Ansonia spinulifer* (Slender Spiny Toad). Vocal Description. *Herpetological Review*, *48*(1), 157–158.

Dehling, J. M. (2010). Advertisement calls of two species of *Microhyla* (Anura: Microhylidae) from Borneo. *Salamandra*, *46*(2), 114–116.

Dehling, J. M. (2010b). A new bush frog (Anura: Rhacophoridae: *Philautus*) from Gunung Mulu National Park, East Malaysia (Borneo). *Salamandra*, *46*, 63–72.

Delgado, D. B., & Haddad, C. F. B. (2015). Calling activity and vocal repertoire of *Hypsiboas prasinus* (Anura, hylidae), a treefrog from the atlantic forest of brazil. *Herpetologica*, *71*(2), 88–95. <https://doi.org/10.1655/HERPETOLOGICA-D-13-00071>

de Sá, R. O., Grant, T., Camargo, A., Heyer, W. R., Ponssa, M. L., & Stanley, E. (2014). Systematics of the neotropical genus *Leptodactylus* Fitzinger, 1826 (Anura: Leptodactylidae): phylogeny, the relevance of non-molecular evidence, and species accounts. *South American Journal of Herpetology*, *9*(s1), S1-S100.

Dias, I. R., Roedder, D., Weinsheimer, F., Kwet, A., & Sole, M. (2011). Description of the advertisement call of *Phasmahyla spectabilis* Cruz, Feio & Nascimento, 2008 (Anura: Phyllomedusinae) with comments on its distribution and reproduction. *Zootaxa*, *2767*, 59-64.

Díaz, L. M., & Cádiz, A. (2007). Guía descriptiva para la identificación de las llamadas de anuncio de las ranas cubanas del género *Eleutherodactylus* (Anura: Leptodactylidae). *Herpetotropicos*, *3*, 100–122.

Díaz, L.M., & Cádiz, A. (2008). Guia taxonómica de los anfibios de Cuba. *ABC Taxa*, 4, 1–294.

Díaz-Gutiérrez, N., Vargas-Salinas, F., Rivera-Correa, M., Rojas-Morales, J. A., Escobar-Lasso, S., Velasco, J. A., & Amézquita, A. (2013). Description of the previously unknown advertisement call and tadpole of the Colombian endemic glassfrog *Centrolene savagei* (Anura: Centrolenidae). *Zootaxa*, *3686*(2), 289–296. <https://doi.org/10.11646/zootaxa.3686.2.9>

Diesmos, A. C., Brown, R. M., & Alcala, A. C. (2002). New species of narrow-mouthed frog (Amphibia: Anura: Microhylidae; genus *Kaloula*) from the mountains of southern Luzon and Polillo Islands, Philippines. *Copeia*, *2002*(4), 1037-1051.

Dodd, C. K. (2013). *Frogs of the united states and canada, 2-vol. Set*. JHU Press.

Doughty, P., & Anstis, M. (2007). A new species of rock-dwelling hylid frog (Anura: Hylidae) from the eastern Kimberley region of Western Australia. *Records-western Australian Museum*, *23*(3), 241–257.

Drummond, L. O., Baeta, D., & Pires, M. R. S. (2007). A new species of *Scinax* (Anura, Hylidae) of the *S*. *ruber* clade from Minas Gerais, Brazil. *Zootaxa*, *1612*(1), 45–53.

Du Preez, L. H., & Carruthers, V. C. (2009). *A Complete Guide to the Frogs of Southern Africa*. Cape Town: Struik Nature.

Duellman, W. E. (1970). *The hylid frogs of Middle America*. Lawrence: Monograph of the Museum of Natural History, University of Kansas. Recuperado de <https://doi.org/10.5962/bhl.title.2835>

Duellman, W. E. (1971). The identities of some ecuadorian hylid frogs. *Herpetologica*, *27*(2), 212–227.

Duellman, W. E. (1972a). A review of the neotropical frogs of the *Hyla bogotensis* group. *Occasional Papers of the Museum of Natural History, University of Kansas*, *11*, 1–31.

Duellman, W. E. (1972b). A new species of *Hyla* from amazonian ecuador. *Copeia*, *1972*(2), 265–271. <https://doi.org/10.2307/1442487>

Duellman, W. E. (1972c). South American frogs of the *Hyla rostrata* group (Amphibia, Anura, Hylidae). *Zoologische Mededelingen*, *47*(14), 177–192.

Duellman, W. E. (1973). Descriptions of new hylid frogs from colombia and ecuador. *Herpetologica*, *29*(3), 219–227.

Duellman, W. E. (1978a). *The biology of an equatorial herpetofauna in Amazonian Ecuador*. Lawrence : University of Kansas,

Duellman, W. E. (1978b). Two new species of eleutherodactylus (Anura: leptodactylidae) from the peruvian andes. Transactions of the Kansas Academy of Science (1903-), 81(1), 65–71. https://doi.org/10.2307/3627358

Duellman, W. E. (1980). The identity of *Centrolenella grandisonae* Cochran and Goin (Anura: Centrolenidae). *Transactions of the Kansas Academy of Science (1903-)*, *83*(1), 26–32. <https://doi.org/10.2307/3627268>

Duellman, W. E. (2005). Cusco amazónico: the lives of amphibians and reptiles in an amazonian rainforest. Ithaca, NY: Cornell University Press.

Duellman, W. E. (2015). *Marsupial frogs: Gastrotheca and allied genera*. Johns Hopkins University Press. Recuperado de <https://muse.jhu.edu/book/40894>

Duellman, W. E., & Trueb, L. (1966). Neotropical hylid frogs, genus *Smilisca*. *University of Kansas publications, Museum of Natural History.*, *17*, 281–375.

Duellman, W. E. and J. M. J. Fouquette. (1968). Middle American frogs of the *Hyla microcephala* group.University of Kansas Publications of the Museum of Natural History 17:517–557

Duellman, W. E., & Crump, M. L. (1974). Speciation in frogs of the *Hyla parviceps* group in the upper amazon basin. *Occasional papers of the Museum of Natural History, the University of Kansas.*, *23*, 1–40.

Duellman, W.E. & Veloso, A. (1977) Phylogeny of Pleurodema (Anura, Leptodactylidae). A biogeographic model. Occasional Papers of the Museum of Natural History, 64, 1–46

Duellman, W. E., & Altig, R. (1978). New species of tree frogs (Family hylidae) from the andes of colombia and ecuador. *Herpetologica*, *34*(2), 177–185.

Duellman, W. E., & Berger, T. J. (1982). A new species of andean treefrog (Hylidae). *Herpetologica*, *38*(4), 456–460.

Duellman, W. E., & Pyles, R. A. (1983). Acoustic resource partitioning in anuran communities. *Copeia*, *1983*(3), 639–649. https://doi.org/10.2307/1444328

Duellman, W. E., & Gray, P. (1983). Developmental biology and systematics of the egg-brooding hylid frogs, genera flectonotus and fritziana. *Herpetologica*, *39*(4), 333–359.

Duellman, W. E., & De Sá, R. O. (1988). A new genus and species of South American hylid frog with a highly modified tadpole. *Tropical Zoology*, *1*(1), 117-136.

Duellman, W. E., J. E. Cadle, and D. C. Cannatella. (1988). A new species of terrestrial *Phyllomedusa* (Anura: Hylidae) from southern Peru. *Herpetologica* 44: 91–95.

Duellman, W. E., & Trueb, L. (1989). Two new treefrogs of the *Hyla parviceps* group from the amazon basin in southern peru. *Herpetologica*, *45*(1), 1–10.

Duellman, W. E., and D. M. Hillis. (1990). Systematics of the *Hyla larinopygion* group. Occasional Papers of the Museum of Natural History, University of Kansas 134: 1–23.

Duellman, W. E., and L. A. Coloma. (1993). *Hyla staufferorum*, a new species of treefrog in the *Hyla larinopygion* group from the cloud forest of Ecuador. Occasional Papers of the Museum of Natural History, University of Kansas 161: 1–11.

Duellman, W.E.; De La Riva, I. & Wild, E.R. (1997). Frogs of the *Hyla armata* and *Hyla pulchella* groups in the Andes of South America, with definitions and analyses of phylogenetic relationships of Andean groups of *Hyla. Scientific Papers of the Natural History Museum, The University of Kansas,* (3):1-41.

Duellman, W.E. (2001) The Hylid Frogs of Middle America. Society for the Study of Amphibians and Reptiles., Ithaca, expanded edition 2v set.

Duellman, W. E., Catenazzi, A., & Blackburn, D. C. (2011). A new species of marsupial frog (Anura: hemiphractidae: gastrotheca) from the Andes of southern Peru. *Zootaxa*, *3095*(1), 1–14.

Duguet R., & Malki F. (2003). *Les Amphibiens de France, Belgique et Luxembourg*. Coll. Parthénope, éditions Biotope, Mèze (France). 480 p.

Dunn, E. R. (1949). Notes on South American Frogs of the family Microhylidae. *American Museum Novitates*, *1419*, 1–21.

Duré, M. I., Schaefer, E. F., & Kehr, A. I. (2015). Acoustic repertoire of *Melanophryniscus cupreuscapularis* (Céspedez and Álvarez 2000) (Anura: Bufonidae): advertisement, encounter, and release calls. *Journal of Herpetology*, *49*(1), 53–59. https://doi.org/10.1670/13-115

Elliott, L., Gerhardt, C., & Davidson, C. (2009). *The frogs and toads of north america: a comprehensive guide to their identification, behavior, and calls*. Boston: Houghton Mifflin Harcourt.

Eterovick, P. C., & Sazima, I. (1998). New species of Proceratophrys (Anura: Leptodactylidae) from southeastern Brazil. *Copeia*, 159-164.

Faivovich, J. (2005). A new species of *Scinax* (Anura: Hylidae) from Misiones, Argentina. *Herpetologica* 61, 69–77.

Faivovich, J., McDiarmid, R. W., & Myers, C. W. (2013). Two new species of Myersiohyla (Anura: Hylidae) from Cerro de la Neblina, Venezuela, with comments on other species of the genus. *American Museum Novitates*, (3792), 1-63.

Fei L. (1999). *Atlas of Amphibians of China*. Zhengzhou: Henan Publishing House of Science and Technology, 1−432

Fei, L., C.-y. Ye, & Jiang, J.-p. (2012). *Colored Atlas of Chinese Amphibians and Their Distributions*. Sichuan, China: Sichuan Publishing House of Science & Technology.

Ferrari, L., & Vaira, M. (2001). Advertisement call and breeding activity of Physalaemus cuqui (Lobo, 1993). *Herpetological Bulletin*, *77*, 20-22.

Forlani, M. C., Mendes, C. V. D. M., Dias, I. R., Ruas, D. S., Tonini, J. F. R., & de Sá, R. O. (2013). The advertisement calls and distribution of two sympatric species of *Chiasmocleis* (Méhely 1904) (Anura, Microhylidae, Gastrophryninae) from the Atlantic Forest. *South american journal of herpetology*, *8*(1), 46-51.

Formas. (1989). A new species of *Eupsophus* (Amphibia, Anura, Leptodactylidae) from southern Chile. *Proceedings of the Biological Society of Washington.*, *102*, 568–576.

Formas, J. R., & Brieva, L. (1994). Advertisement calls and relationships of chilean frogs eupsophus contulmoensis and e-insularis(Amphibia, anura, leptodactylidae). *Proceedings of the Biological Society of Washington.*, *107*, 391–397.

Formas, J. R., & Vera, M. I. (1882). The status of two chilean frogs of the genus Eupsophus (Anura: leptodactylidae). *Proceedings of the Biological Society of Washington*, *95*, 668–693.

Forti, L. R., Strüssmann, C., & Mott, T. (2010). Acoustic communication and vocalization microhabitat in *Ameerega braccata* (Steindachner, 1864) (Anura, dendrobatidae) from Midwestern Brazil. *Brazilian Journal of Biology*, *70*(1), 211–216. https://doi.org/10.1590/S1519-69842010000100029

Forti, L. R., & Castanho, L. M. (2012). Behavioural repertoire and a new geographical record of the torrent frog Hylodes cardosoi (Anura: Hylodidae). *Herpetological Bulletin*, *121*, 17-22.

Funk, W. C., & Cannatella, D. C. (2009). A new, large species of Chiasmocleis Méhelÿ 1904 (Anura: Microhylidae) from the Iquitos region, Amazonian Peru. *Zootaxa*, *2247*(1), 37-50.

Fouquet, A., Gaucher, P., Blanc, M., & Velez-Rodriguez, C. M. (2007). Description of two new species of Rhinella (Anura: bufonidae) from the lowlands of the Guiana shield. *Zootaxa*, *1663*(1), 17–32. <https://doi.org/10.11646/zootaxa.1663.1.2>

Furtado, R., Santos, S. P., Dias, T. M., Bastos, R. P., & Nomura, F. (2016). Vocal repertoire during reproductive and aggressive contexts of three brazilian tree frogs: Bokermannohyla sapiranga, Hypsiboas albopunctatus and H. goianus (Anura: hylidae). *South American Journal of Herpetology*, *11*(2), 136–147. <https://doi.org/10.2994/SAJH-D-16-00002.1>

Gaiga, R., C. Loiola, S. Mângia, and R. M. Pirani. (2013). Advertisement call and tadpoles of *Bokermannohyla vulcaniae* (Vasconcelos and Giaretta, 2003) (Amphibia: Anura: Hylidae). South American Journal of Herpetology 8: 127–131.

Galvis, P. A., Caorsi, V. Z., Sánchez-Pacheco, S. J., & Rada, M. (2016). The advertisement calls of three hylid frogs from Hispaniola. *Bioacoustics*, *25*(1), 89–97. https://doi.org/10.1080/09524622.2015.1116410

Galvis, P. A., Caorsi, V. Z., Sánchez-Pacheco, S. J., & Rada, M. (2018). The advertisement calls of three *Eleutherodactylus* species from Hispaniola (Anura: eleutherodactylidae). *Bioacoustics*, *27*(1), 1–12. <https://doi.org/10.1080/09524622.2016.1260053>

Garcia, P. C. de A., J. Faivovich, and C. F. B. Haddad. (2007). Redescription of *Hypsiboas semiguttatus*, with the description of a new species of the *Hypsiboas pulchellus* group. Copeia 2007: 933–951.

Garcia, P. C. A., & Haddad, C. F. B. (2008). Vocalizations and comments on the relationships of Hypsiboas ericae (Amphibia, hylidae). *Iheringia. Série Zoologia*, *98*(1), 161–166. <https://doi.org/10.1590/S0073-47212008000100021>

Garcia, P.C.A., Berneck, B.V.M., Costa, C.O.R. (2009): A new species of *Paratelmatobius* (Amphibia, Anura, Leptodactylidae) from Atlantic Rain Forest of Southeastern Brazil. South American Journal of Herpetology **4**: 217–224.

Garcia-Rutledge, E. J., & Narins, P. M. (2001). Shared acoustic resources in an old world frog community. *Herpetologica*, *57*(1), 104–116.

Garda, A. A., Pedro, V. de A. S., & Lion, M. B. (2010). The advertisement and release calls of *Rhinella jimi* (Anura, bufonidae). *South American Journal of Herpetology*, *5*(2), 151–156. <https://doi.org/10.2994/057.005.0209>

Garda, A. A., Santana, D. J., & São-Pedro, V. A. (2010). Taxonomic characterization of Paradoxical frogs (Anura, Hylidae, Pseudae): geographic distribution, external morphology, and morphometry. *Zootaxa*, *2666*, 1-28.

Garey, M. V., Lima, A. M. X., Hartmann, M. T., & Haddad, C. F. B. (2012a). A new species of miniaturized toadlet, genus *Brachycephalus* (Anura: brachycephalidae), from southern brazil. *Herpetologica*, *68*(2), 266–271. <https://doi.org/10.1655/HERPETOLOGICA-D-11-00074.1>

Garey, M. V., Costa, T. R., de Lima, A. M., Toledo, L. F., & Hartmann, M. T. (2012b). Advertisement call of *Scinax littoralis* and *S. angrensis* (Amphibia: Anura: Hylidae), with notes on the reproductive activity of S. littoralis. *Acta Herpetologica*, *7*(2), 297-308.

Garey, M. V.; Provete, D.B.; Ouchi-Melo, L. S.; Haddad, C.F.B.; Rossa-Feres, D.C. (2018). The larva and advertisement call of *Bokermannohyla ahenea* (Anura: Hylidae). *South American Journal of Herpetology in press.*

Garg, S., Suyesh, R., Sukesan, S., & Biju, S. D. (2017). Seven new species of Night Frogs (Anura, Nyctibatrachidae) from the Western Ghats Biodiversity Hotspot of India, with remarkably high diversity of diminutive forms. *PeerJ*, 5, e3007.

Giaretta, A.A., Castanho, L.M. (1990): Nova espécie de *Paratelmatobius* (Amphibia, Anura, Leptodactylidae) da Serra do Mar, Brasil. *Papéis Avulsos de Zoologia* **37**: 133–139.

Giaretta, A. A., & Cardoso, A. J. (1995). Reproductive behavior of *Cycloramphus dubius* miranda-ribeiro(Amphibia, anura, leptodactylidae). *Revista Brasileira de Zoologia*, *12*(2), 233–237. <https://doi.org/10.1590/S0101-81751995000200002>

Giaretta, A.A., J. Oliveira-Filho and M.N.C. Kokubum. (2007). A new *Phyllomedusa* Wagler (Anura, Hylidae) with reticulated pattern on flanks from Southeastern Brazil. *Zootaxa* 1614: 31-41.

Giaretta, A. A., Toffoli, D., & Oliveira, L. E. (2007). A new species of Ischnocnema (Anura: Eleutherodactylinae) from open areas of the Cerrado Biome in southeastern Brazil. *Zootaxa*, *1666*(1), 43–51. <https://doi.org/10.11646/zootaxa.1666.1.3>

Giaretta, A. A., Martins, L. B., & Santos, M. P. (2009). Further notes on the taxonomy of four species of *Physalaemus* (Anura, Leiuperidae) from the Atlantic Forest of Southeastern Brazil. *Zootaxa*, *2266*, 51-60.

Giaretta, A., & Martins, L. (2009). Notes on the call and behavior of *Arcovomer passarellii* (Anura: Microhylidae). *Herpetology Notes*, *2*, 91-93.

Giaretta, A. A., & Magrini, Leandro. (2013). Calls of Paratelmatobius gaigeae (Cochran 1938) (Anura, Leptodactylidae). *Herpetology Notes*, *6*, 171–176.

Giasson, L. O. M., & Haddad, C. F. B. (2006). Social interactions in hypsiboas albomarginatus (Anura: hylidae) and the significance of acoustic and visual signals. *Journal of Herpetology*, *40*(2), 171–180. https://doi.org/10.1670/205-05A.1

Gingras, B., Boeckle, M., Herbst, C. T., & Fitch, W. T. (2013). Call acoustics reflect body size across four clades of anurans. *Journal of Zoology*, *289*(2), 143–150. https://doi.org/10.1111/j.1469-7998.2012.00973.x

Glaw, F., & Vences, M. (1991). Bioacoustic differentiation in Painted frogs (Discoglossus). *Amphibia-Reptilia*, *12*(4), 385–394. <https://doi.org/10.1163/156853891X00031>

Glaw, F., and M. Vences. (1992). *A Field guide to the Amphibians and Reptiles of Madagascar*. Köln: Moos Druck.

Glaw, F., and M. Vences. (1994). *A Field guide to the Amphibians and Reptiles of Madagascar*. Second Edition, Including Mammals and Freshwater Fish. Köln: Moos Druck.

Glaw F, Vences M. (2007) *A Field Guide to the Amphibians and Reptiles of Madagascar.* Third edition*.* Köln:, Vences & Glaw.

Glaw, F., Vences, M., & Böhme, W. (1998). Systematic revision of the genus *Aglyptodactylus* Boulenger, 1919 (Amphibia: Ranidae), and analysis of its phylogenetic relationships to other Madagascan ranid genera (Tomopterna, Boophis, Mantidactylus, and Mantella). *Journal of Zoological Systematics and Evolutionary Research*, *36*(1‐2), 17-37.

Glaw, F., Vences, M., & Gossmann, V. (2000). A new species of *Mantidactylus* (Subgenus guibemantis) from Madagascar, with a comparative survey of internal femoral gland structure in the genus (Amphibia: ranidae: mantellinae). *Journal of Natural History*, *34*(7), 1135–1154. https://doi.org/10.1080/00222930050020140

Glaw, F., Vences, M., Andreone, F., & Vallan, D. (2001). Revision of the *Boophis majori* group (Amphibia: Mantellidae) from Madagascar, with descriptions of five new species. *Zoological Journal of the Linnean Society*, *133*(4), 495-529.

Glaw, F., Koehler, J., De la Riva, I., Vieites, D. R., & Vences, M. (2010). Integrative taxonomy of Malagasy treefrogs: combination of molecular genetics, bioacoustics and comparative morphology reveals twelve additional species of Boophis. *Zootaxa*, *2383*(1), 1-82.

Glos, J., Glaw, F., & Vences, M. (2005). A new species of Scaphiophryne from western Madagascar. *Copeia*, *2005*(2), 252-261.

Godinho, L. B., Moura, M. R., Lacerda, J. V. A., & Feio, R. N. (2013). A new species of Proceratophrys (Anura: Odontophrynidae) from the middle São Francisco River, southeastern Brazil. *Salamandra*, *49*(2), 63-73.

Gomes, F. B. R., & Martins, I. A. (2006). Amphibia, Anura, Hylidae, *Dendropsophus anceps* (Lutz, 1929): filling gap, geographic distribution map and vocalization. *Check List*, *2*(3), 22.

Goin, C. J., & Woodley, J. D. (1969). A new tree‐frog from Guyana. *Zoological Journal of the Linnean Society*, *48*(1), 135-140.

Gordo, M., Toledo, L. F., Suárez, P., Kawashita-Ribeiro, R. A., Ávila, R. W., Morais, D. H., & Nunes, I. (2013). A new species of Milk Frog of the genus *Trachycephalus* Tschudi (Anura, Hylidae) from the Amazonian rainforest. *Herpetologica*, *69*(4), 466-479.

Goutte, S., Dubois, A., Howard, S. D., Marquez, R., Rowley, J. J. L., Dehling, J. M., Legendre, F. (2016). Environmental constraints and call evolution in torrent-dwelling frogs. *Evolution*, *70*(4), 811–826. https://doi.org/10.1111/evo.12903

Grafe, T. U., Preininger, D., Sztatecsny, M., Kasah, R., Dehling, J. M., Proksch, S., & Hödl, W. (2012). Multimodal communication in a noisy environment: a case study of the bornean rock frog staurois parvus. *PLOS ONE*, *7*(5), e37965. https://doi.org/10.1371/journal.pone.0037965

Grandison, A. G. C., & Grandison, A. G. C. (1961). Chilean species of the genus Eupsophus (Anura: leptodactylidae). *Bulletin of the British Museum (Natural History).*, *8*, 111–149.

Grant, T., Bolivar-G., W., & Castro, F. (1998). The advertisement call of centrolene geckoideum. *Journal of Herpetology*, *32*(3), 452–455. <https://doi.org/10.2307/1565465>

Grant, T., & Rodríguez, L. O. (2001). Two new species of frogs of the genus *Colostethus* (Dendrobatidae) from Peru and a redescription of *C. trilineatus* (Boulenger, 1883). *American Museum Novitates*, *3355*, 1–24. https://doi.org/10.1206/0003-0082(2001)355<0001:TNSOFO>2.0.CO;2

Grosjean, S., & Dubois, A. (2001). Description of advertisement calls of five *Bufo* species (Bufonidae) from South and South-east Asia. *Hamadryad*, *26*, 235-246.

Grosjean, S., and Dubois, A. (2011) Description of the Advertisement Calls of Nine Species of *Fejervarya* Bolkay, 1915 and *Minervarya* Dubois, Ohler & Biju, 2001 from China, India and Nepal. Alytes, 27, 117–41

Guarnizo, C. E., Escallón, C., Cannatella, D., & Amézquita, A. (2012). Congruence between acoustic traits and genealogical history reveals a new species of *Dendropsophus* (Anura: hylidae) in the high andes of colombia. *Herpetologica*, 68(4), 523–540. https://doi.org/10.1655/HERPETOLOGICA-D-10-00038

Guayasamin, J. M., & Arteaga, A. F. (2013). A new species of the *Pristimantis orestes* group (Amphibia: strabomantidae) from the high Andes of Ecuador, Reserva Mazar. *Zootaxa*, 3616(4). <https://doi.org/10.11646/zootaxa.3616.4.3>

[Guerra, C., D. Baldo, S. D. Rosset, C. Borteiro, and F. Kolenc](http://research.amnh.org/vz/herpetology/5_6amphibia/?action=names&a_id=471) . ([2011](http://research.amnh.org/vz/herpetology/5_6amphibia/?action=names&year=2011)). Advertisement and release calls in Neotropical toads of the *Rhinella granulosa* group and evidence of natural hybridization between *R. bergi* and *R. major* (Anura: Bufonidae). [*Zootaxa*](http://research.amnh.org/vz/herpetology/5_6amphibia/?action=names&p_id=3) 3092: 26-42.

Guerra, V., de Morais, A. R., Gambale, P. G., Oda, F. H., & Pereira Bastos, R. (2017). Variation of the advertisement call of Physalaemus centralis Bokermann, 1962 (Anura: Leptodactylidae) in the Cerrado of central Brazil. *Studies on Neotropical Fauna and Environment*, *52*(2), 103-111.

Guimarães, L. D., L. P. Lima, R. F. Juliano, And R. P. Bastos. (2001). Vocalizações de espécies de anuros (Amphibia) no Brasil central. *Boletim do Museu Nacional, Nova Série, Zoologia*, 474:1-14

Guimarães, C. S., Lacerda, J. V. A., & Feio, R. N. (2013). Advertisement call of Zachaenus carvalhoi Izecksohn, 1982 (Anura: cycloramphidae) from southeastern Brazil. *Zootaxa*, *3718*(4), 398–400. <https://doi.org/10.11646/zootaxa.3718.4.9>

Richards, S. J. (2001). A new species of torrent-dwelling frog (Anura: Hylidae: *Litoria*) from the mountains of Indonesian New Guinea (West Papua). Memoirs of the Queensland Museum 46: 733–740.

Günther, R. (2003a). First record of the microhylid frog genus Cophixalus from western Papua, Indonesia, with descriptions of two new species. *Herpetozoa*, *16*(1/2), 3-21.

Günther, R. (2003b). Three new species of the genus Oreophnyiie from western Papua, Indonesia (Amphibia, Anura, Microhylidae). *Spixiana* 26/2: 175-191

Günther, R. (2004), Description of a new treefrog species from western New Guinea showing extreme colour polymorphism (Anura, Hylidae, *Litoria*). *Zoosystematics and Evolution*, 80: 251-259. doi:[10.1002/mmnz.20040800205](https://doi.org/10.1002/mmnz.20040800205)

Günther, R. (2006). Derived reproductive modes in New Guinean anuran amphibians and description of a new species with paternal care in the genus *Callulops* (Microhylidae). *Journal of Zoology*, *268*(2), 153–170. <https://doi.org/10.1111/j.1469-7998.2005.00007.x>

Günther, R. (2008). Two new hylid frogs (Anura: Hylidae: Litoria) from western New Guinea. *Vertebrate Zoology*, *58*, 83.

Günther, R. (2009). A new and minute species of Austrochaperina (Amphibia: Anura: Microhylidae) from western New Guinea. *Vertebrate Zoology*, *59*(1), 81-89.

Günther, R. (2010a). Another new Cophixalus species (Amphibia: Anura: Microhylidae) from western New Guinea. *Bonn Zoological Bulletin*, *57*(2), 231-240.

Günther, R. (2010b). Description of a new microhylid frog species of the genus Xenorhina (Amphibia: Anura: Microhylidae) from the Fakfak Mountains, far western New Guinea. Vertebrate Zoology 60: 217–224.

Günter, R. and S. J. Richards. (2000). A new species of the *Litoria gracilenta* group from Irian Jaya (Anura: Hylidae). *Herpetozoa* 13:27–43.

Günter, R., Richards & D. Iskandar. (2001). Two new species of the genus *Oreophryne* from Irian Jaya, Indonesia (Amphibia, Anura, Microhylidae). *Spixiana* 24(3):257-274

Günther, R., S. J. Richards, B. Tjaturadi, and D. T. Iskandar. (2009). A new species of the microhylid frog genus *Oreophryne* from the Mamberamo Basin of northern Papua Province, Indonesian New Guinea. *Vertebrate Zoology* 59: 147–155.

Günther, R., and S. J. Richards. (2011). Five new microhylid frog species from Enga Province, Papua New Guinea, and remarks on *Albericus alpestris* (Anura, Microhylidae). *Vertebrate Zoology* 61: 343–372.

Günther, R., S. J. Richards, D. P. Bickford, and G. R. Johnston. (2012). A new egg-guarding species of *Oreophryne* (Amphibia, Anura, Microhylidae) from southern Papua New Guinea. Zoosystematics and Evolution. *Mitteilungen aus dem Museum für Naturkunde in Berlin* 88: 223–230.

Gururaja, K.V., Ramachandra, T.V. (2006). '*Pedostibes tuberculosis*, advertisement call and distribution. *Herpetological Review*, 37(1), 75-76.

Haddad, C.F.B., and A.J. Cardoso. (1987). Taxonomia de três espécies de *Pseudopaludicola* (Anura, Leptodactylidae). *Papéis Avulsos de Zoologia* 36:287–300.

Haddad, C. F., & Pombal Jr, J. P. (1987). *Hyla hiemalis*, nova espécie do grupo rizibilis do Estado de São Paulo (Amphibia, Anura, Hylidae). *Revista Brasileira de Biologia*, *47*(1/2), 127-132.

Haddad, C. F. B., & Sazima, I. (1989). A new species of Cycloramphus from southeastern Brazil (Amphibia: leptodactylidae). *Herpetologica*, *45*(4), 425–429.

Haddad, C. F. B., & Martins, M. (1994). Four species of brazilian poison frogs related to *Epipedobates pictus* (Dendrobatidae): taxonomy and natural history observations. *Herpetologica*, *50*(3), 282–295.

Haddad, C. F., Pombal Jr, J. P., & Batistic, R. F. (1994). Natural hybridization between diploid and tetraploid species of leaf-frogs, genus Phyllomedusa (Amphibia). *Journal of Herpetology*, 28(4), 425-430.

Haddad, C. F., & Pombal Jr, J. P. (1995). A new species of *Hylodes* from southeastern Brazil (Amphibia: Leptodactylidae). *Herpetologica*, 279-286.

Haddad, C. F., & Pombal Jr, J. P. (1998). Redescription of *Physalaemus spiniger* (Anura: Leptodactylidae) and description of two new reproductive modes. *Journal of Herpetology*, 557-565.

Haddad, C. F., & Giaretta, A. A. (1999). Visual and acoustic communication in the Brazilian torrent frog, *Hylodes asper* (Anura: Leptodactylidae). *Herpetologica*, 324-333.

Haddad, C.F.B.; P.C.A. Garcia & J.P. Pombal Jr. (2003). Redescrição de *Hylodes perplicatus* (Miranda-Ribeiro, 1926) (Amphibia, Anura, Leptodactylidae). *Arquivos do Museu Nacional* 61(4): 245-254.

Haddad, C. F., & Sazima, I. (2004). A new species of Physalaemus (Amphibia; Leptodactylidae) from the Atlantic forest in southeastern Brazil. *Zootaxa*, *479*(1), 1-12.

Haddad, C. F. B., Faivovich, J., & Garcia, P. C. A. (2005). The specialized reproductive mode of the treefrog *Aplastodiscus perviridis* (Anura: hylidae). *Amphibia-Reptilia*, *26*(1), 87–92. <https://doi.org/10.1163/1568538053693224>

Haddad, C. F. B., Giovanelli, J. G. R., Giasson, L. O. M., & Toledo, L. F. (2005). Guia sonoro dos anfíbios anuros da Mata Atlântica. *Commercial digital media. Manaus: NovoDisc Mídia Digital da Amazônia Ltda, Brazil*.

Haddad, C. F. B., Toledo, L. F., Prado, C. P. A., Loebmann, D., Gasparini, J. L., & Sazima, I. (2013). *Guia dos anfíbios da Mata Atlântica – diversidade e biologia*. São Paulo: Anolis Books.

Hamer, A., & Organ, A. (2008). Aspects of the ecology and conservation of the Growling Grass Frog Litoria raniformis in an urban-fringe environment, southern Victoria. *Australian Zoologist*, *34*(3), 393-407.

Hampson, K., & Bennett, D. (1998) Love Songs From Coorg. Frog calls from the Western Ghats of India.

Hampson, K., & Bennett, D. (2002). Advertisement calls of amphibians at Lackunda estate, Coorg, Karnataka. In:. *Frogs of Coorg, Karnataka, India*. (Bennett, D eds), Glossop Viper press, pp 121-135.

Harper, E. B., Measey, G. J., Patrick, D. A., Menegon, M., & Vonesh, J. R. (2010). *Field guide to amphibians of the eastern arc mountains and coastal forests of tanzania and kenya*. Nairobi, Kenya: Camerapix Publishers International.

Hartmann, M. T., P. A. Hartmann, and C. F. B. Haddad. (2002). Advertisement calls of *Chiasmocleis carvalhoi*, *Chiasmocleis mehelyi*, and *Myersiella microps* (Microhylidae). *Journal of Herpetology* 36:509–511.

Hartmann, M. T., Hartmann, P. A., & Haddad, C. F. B. (2004). Visual signaling and reproductive biology in a nocturnal treefrog, genus *Hyla*(Anura: hylidae). *Amphibia-Reptilia*, *25*(4), 395–406. https://doi.org/10.1163/1568538042788933

Harvey, M. B., & Keck, M. B. (1995). A new species of ischnocnema (Anura: leptodactylidae) from high elevations in the andes of central bolivia. *Herpetologica*, *51(1)*, 56–66.

Harvey, M. B., Almendariz, A., Brito M, J., & Batallas, D. (2013). A new species of noblella (Anura: craugastoridae) from the amazonian slopes of the ecuadorian andes with comments on noblella lochites(Lynch). *Zootaxa*, *3635*, 1–14.

Hedges, S. B. (1990). A new species of *Phrynopus* (Anura: Leptodactylidae) from perú. *Copeia*, *1990(1)*, 108. https://doi.org/10.2307/1445826

Hedges, S. B., Thomas, R., & Franz, R. (1987). A new species of *Eleutherodactylus* (Anura:Leptodactylidae) from the Massif de la Hotte, Haiti. *Copeia*, *1987(4)*, 943–949.

Hedges, S. B., & Thomas R. (1992). Two new species of *Eleutherodactylus* from remnant cloud forest in Haiti (Anura, Leptodactylidae). *Herpetologica*, *48*, 351–358.

Henderson, R. W., & Powell, R. (2009). *Natural history of west indian reptiles and amphibians*. Gainesville: University Press of Florida.

Hernández, M., Alonso, R., & Rodríguez, A. (2010). Advertisement call of *Peltophryne florentinoi*(Anura: bufonidae), an endemic toad from Zapata Swamp, Cuba. *Amphibia-Reptilia*, *31*(2), 265–272. <https://doi.org/10.1163/156853810791069083>

Hepp, F. S. F. S., & Carvalho-e-Silva, S. P. (2011). Description and comparison of advertisement calls of Euparkerella brasiliensis (Parker, 1926) and E. cochranae Izecksohn, 1988 (Amphibia: Anura: Strabomantidae). *Herpetology Notes*, *4*, 45–51.

Hepp, F. S. F. S., Luna-Dias, C. D., Gonzaga, L. P., & Carvalho-E-Silva, S. P. D. (2012). Redescription of the advertisement call of *Dendropsophus seniculus* (Cope, 1868) and the consequences for the acoustic traits of the *Dendropsophus marmoratus* species group (Amphibia: anura: dendropsophini). *South American Journal of Herpetology*, *7(2)*, 165–171. https://doi.org/10.2994/057.007.0206

Heyer, W. R. (1971). Mating calls of some frogs from Thailand. *Fieldiana Zoology*, *58*, 61–82

Heyer, W. R. (1973). Systematics of the *marmoratus* group of the frog genus *Leptodactylus* (Amphibia, Leptodactylidae). *Contributions in Science*, 251: 1-50.

Heyer, W. R. (1977). Taxonomic notes on frogs from the Madeira and Purus Rivers, Brasil. *Papéis Avulsos de Zoologia*, *31(8)*, 141–162.

Heyer, W. R. (1978). Systematics of the *fuscus* group of the frog genus *Leptodactylus* (Amphibia, Leptodactylidae). *National History Museum of Los Angeles County, Scientific Bulletin*, 8(29): 1-85.

Heyer, W. R. (1980). The calls and taxonomic positions of *Hyla giesleri* and *Ololygon opalina* (Amphibia: Anura: Hylidae). *Proceedings of the Biological Society of Washington*, *93(3)*, 655–661.

Heyer, W. R. (1983a). Notes on the frog genus *Cycloramphus* (Amphibia: Leptodactylidae), with descriptions of two new species. *Proceedings of the Biological Society of Washington*, *96(3)*, 548–559.

Heyer, W. R. (1983b). Variation and systematics of frogs of the genus *Cycloramphus* (Amphibia, Leptodactylidae). *Arquivos de Zoologia*, *30(4)*, 235–339.

Heyer, W. R. (1984). Variation, systematics, and zoogeography of Eleutherodactylus guentheri and closely related species (Amphibia: Anura: Leptodactylidae). *Smithsonian Contributions to Zoology*, *402*, 1–42. Available at <http://repository.si.edu//handle/10088/5532>

Heyer, W. R. (2005). Variation and taxonomic clarification of the large species of the *Leptodactylus pentadactylus* species group (Amphibia: Leptodactylidae) from Middle America, northern South America, and Amazonia. *Arquivos de Zoologia,* 37(3): 269-348.

Heyer, W. R. and Pyburn, W. F. (1983). *Leptodactylus riveroi*, a new frog species from Amazonia, South America (Anura: Leptodactylidae). *Proceedings of the Biological Society of Washington*, 96(3): 560-566.

Heyer, W. R., Rand, A. S., Cruz, C. A. G. da, Peixoto, O. L., & Nelson, C. E. (1990). Frogs of Boracéia. *Arquivos de Zoologia*, *31*(4), 231–410. <https://doi.org/10.11606/issn.2176-7793.v31i4p231-410>

Heyer, W. R., Heyer, W. R., & Hardy, L. M. (1991). A new species of frog of the *Eleutherodactylus lacrimosus* assembly from Amazonia, South America (Amphibia: anura: leptodactylidae). *Proceedings of the Biological Society of Washington*, *104*, 436–447.

Heyer, W. R. and Gascon, C. (1995). Collection notes and call characteristics for *Ischnocnema quixensis* and *Phyzelaphryne miriamae* (Amphibia: Anura; Leptodactylidae). *Journal of Herpetology*, *29(2)*, 304–307.

Heyer, W. R., Garcia Lopez, J. M. and Cardoso, A. J. (1996). Advertisement call variation in the *Leptodactylus mystaceus* species complex (Amphibia: Leptodactylidae) with a description of a new sibling species. *Amphibia-Reptilia*, 17(1): 7-31.

Heyer, W. R. and Juncá, F. A. (2003). *Leptodactylus caatingae*, a new species of frog from eastern Brazil (Amphibia: Anura: Leptodactylidae). *Proceedings of the Biological Society of Washington*, 116(2): 317-329.

Heyer, W. R. and Heyer, M. M. (2004). *Leptodactylus furnarius* Sazima and Bokermann cerrado oven frog. *Catalogue of American Amphibians and Reptiles*, 785: 1-5.

Hillis, D. M., & de Sá, R. O. (1988). Phylogeny and taxonomy of the Rana palmipes group (Salientia: Ranidae). *Herpetological Monographs*, *2*, 1–26.

Hödl, W. (1992). Reproductive behavior in the neotropical foam-nesting frog *Pleurodema diplolistris* (Leptodactylidae). *Amphibia-Reptilia* 13: 263-274.

Hoogmoed, M.S., Borges, D.M., & Cascon, P. (1994). Three new species of the genus *Adelophryne* (Amphibia: Anura: Leptodactylidae) from northeastern Brazil, with remarks on the other species of the genus. *Zoologische Mededelingen*, *68*(24), 271–300.

Hoskin, C. J. (2004). Australian microhylid frogs (Cophixalus and Austrochaperina): phylogeny, taxonomy, calls, distributions and breeding biology. *Australian Journal of Zoology*, *52*(3), 237-269.

Hoskin, C. J. (2010). Breeding behaviour of the Barred Frog Mixophyes coggeri. *Memoirs of the Queensland Museum-Nature*, *55*, 1-7.

Hoskin, C. J. (2012). Two new frog species (Microhylidae: Cophixalus) from the Australian Wet Tropics region, and redescription of Cophixalus ornatus. *Zootaxa*, *3271*, 1-16.

Hoskin, C. J. (2013). A new frog species (Microhylidae: Cophixalus) from boulder-pile habitat of Cape Melville, north-east Australia. *Zootaxa*, *3722*, 61-72.

Hoskin, C. J. (2014). The advertisement call of the cape melville treefrog *Litoria andiirrmalin* (Anura: hylidae). *Zootaxa*, *3786*(4), 499–500. <https://doi.org/10.11646/zootaxa.3786.4.8>

Hoskin, C. J., & Goosem, M. W. (2010). Road impacts on abundance, call traits, and body size of rainforest frogs in northeast Australia. *Ecology and society*, *15*(3).

Hoskin, C. J., & Aland, K. (2011). Two new frog species (Microhylidae: Cophixalus) from boulder habitats on Cape York Peninsula, north-east Australia. *Zootaxa*, *3027*(1), 39-51.

Huang, W.-S., Lee, J.-K., & Ho, C.-H. (2001). Reproductive patterns of two sympatric rhacophorid frogs, *Buergeria japonica* and *B*. *robusta*, with comments on anuran breeding seasons in Taiwan. *Zoological Science*, *18*(1), 63–70. https://doi.org/10.2108/zsj.18.63

Hutter, C. R., and J. M. Guayasamin. (2012). A new cryptic species of glassfrog (Centrolenidae: *Nymphargus*) from Reserva Las Gralarias Ecuador. *Zootaxa*, *3257*, 1–21.

Hutter, C. R., Esobar-Lasso, S., Rojas-Morales, J. A., Gutiérrez-Cárdenas, P. D. A., Imba, H., & Guayasamin, J. M. (2013). The territoriality, vocalizations and aggressive interactions of the red-spotted glassfrog, *Nymphargus grandisonae*, Cochran and Goin, 1970 (Anura: centrolenidae). *Journal of Natural History*, *47*(47–48), 3011–3032. https://doi.org/10.1080/00222933.2013.792961

Ibáñez, R. D., Rand, A. S., & Jaramillo, C. A. A. (1999). Los anfibios del monumento natural Barro Colorado. Parque Nacional Soberania y areas adyacentes. D’Vini Editorial Ltda, Santa Fé de Bogotá, 192 p.

Inger, R. F. (1954). Systematics and zoogeography of Philippine Amphibia. *Fieldiana. Zoology*, *33*, 183–531.

Inger, R. F. (1964). Two new species of frogs from Borneo. *Fieldiana Zoology*, *44*, 151–159.

Inger, R. F. (1966). The systematics and zoogeography of the amphibia of Borneo. *Fieldiana Zoology*, *52*, 1–402. <https://doi.org/10.5962/bhl.title.3147>

Inger, R. F., Shaffer, H. B., Koshy, M., & Bakde, R. (1984). A report on a collection of amphibians and reptiles from the Ponmudi, Kerala, South India. *Journal of the Bombay Natural History Society*, *81*(2), 406–427.

Inger, R. F., & Stuebing, R. B. (1996). Two new species of frogs from southeastern Sarawak. Raffles Bulletin of Zoology. *Singapore*, *44*, 543–549.

Inger, R. F., Orlov, N., & Darevsky, I. (1999). Frogs of Vietnam: a report on new collections. *Fieldiana Zoology*, *92*, 1–46.

Izecksohn, E., & Carvalho-e-Silva, S. P. de. (2008). The species of *Gastrotheca* Fitzinger at Organs Mountains, Rio de Janeiro state, Brazil (Amphibia: Anura: Amphignathodontidae). *Revista Brasileira de Zoologia*, *25*(1), 100–110. <https://doi.org/10.1590/S0101-81752008000100014>

Izecksohn, E., Carvalho-e-silva, S. P., & Peixoto, O. L. (2009). Sobre *Gastrotheca fissipes* (Boulenger, 1888), com descrição de uma nova espécie (Amphibia, Anura, Amphignathodontidae). *Arquivos do Museu Nacional*, *67*(1-2), 81-91.

Jared, C., Antoniazzi, M. M., Katchburian, E., Toledo, R. C., & Freymüller, E. (1999). Some aspects of the natural history of the casque-headed tree frog Corythomantis greeningi Boulenger (Hylidae). *Annales Des Sciences Naturelles - Zoologie et Biologie Animale*, *20*(3), 105–115. <https://doi.org/10.1016/S0003-4339(00)86975-0>

Jiang, J.-P., Xie, F., Fei, L., Ye, C. & Zheng, M-Q. (2002) Mating calls of six forms of pelobatid in Wawu Mountain national forest park, Sichuan, China (Anura: Pelobatidae). *Zoological Research,* 23, 89–94.

Johnston, G. R., & Richards, S. J. (1994). A new species of Litoria (Anura: Hylidae) from New Guinea and a redefinition of Litoria leucova (Tyler, 1968). *Memoirs of the Queensland Museum*, *37*, 273-279.

Juncá, F. A. (1996). Parental care and egg mortality in colostethus stepheni. *Journal of Herpetology*, *30*(2), 292–294. https://doi.org/10.2307/1565530

Juncá, F. A., Röhr, D. L., Lourenço-de-Moraes, R., Santos, F. J. M., Protázio, A. S., Mercês, E. A., & Solé, M. (2012). Advertisement call of species of the genus *Frostius* Cannatella 1986 (Anura: bufonidae). *Acta Herpetologica*, *7*(2), 189–201. <https://doi.org/10.13128/Acta_Herpetol-9898>

Juncá, F.A., Napoli, M.F., Cedraz, J. & Nunes, I. (2012) Acoustic characteristics of the advertisement and territorial calls ofPhyllodytes tuberculosus Bokermann, 1966 (Amphibia: Anura: Hylidae). *Zootaxa*, 3506, 87–88.

Kadadevaru, G. G., Kanamadi, R. D., & Schneider, H. (1998). Mating call of the burrowing frog, *Ramanella montana* (Jerdon 1859). *Journal of Advanced Zoology*, *19*, 91-93.

Kadadevaru, G. G., Kanamadi, R. D., & Schneider, H. (2000). Advertisement call of two Indian ranids, *Indirana beddomii* and *Tomopterna rufescens*. *Amphibia-Reptilia*, *21*(2), 242–246.

Kaefer, I. L., Erdtmann, L. K., & Lima, A. P. (2011). The advertisement call of Physalaemus ephippifer (Anura: Leiuperidae) from Brazilian Amazonia. *Zootaxa*, *2929*(1), 57-58.

Kaefer, I. L., Tsuji-Nishikido, B. M., & Lima, A. P. (2012). Beyond the river: underlying determinants of population acoustic signal variability in Amazonian direct-developing *Allobates* (Anura: Dendrobatoidea). *Acta Ethologica*, *15*(2), 187–194. https://doi.org/10.1007/s10211-012-0126-0

Kaiser, H., Coloma, L. A., & Gray, H. M. (1994). A new species of Colostethus (Anura: Dendrobatidae) from Martinique, French Antilles. *Herpetologica*, *50*(1), 23–32.

Kanamadi, R. D., Schneider, H., Hiremath, C. R., & Jirankali, C. S. (1993b). Vocalization of the tree frog *Polypedates maculatus* (Rhacophoridae). *Journal of Biosciences*, *18*(2), 239–245. https://doi.org/10.1007/BF02703121

Kanamadi, R. D., Hiremath, C. R., & Schneider, H. (1994). Advertisement calls of two anuran amphibians, *Rana tigrina* and *Tomopterna breviceps*. *Journal of Biosciences*, *19*, 75–80.

Kanamadi, R. D., Kadadevaru, G. G., & Schneider, H. (2001). Calling behavior, bioacoustics and distribution of a rhacophorid frog, *Philautus variabilis* (Gunther, 1858). *Amphibia-Reptilia*, *22*, 365–372.

Kasuya, E., Kumaki, T., & Saiio, T. (1992). Vocal repertoire of the japanese treefrog, rhacophorus arboreus(Anura: rhacophoridae)(Behavior biology). *Zoological Science*, *9*, 469–473.

[Khan, M. S.](http://research.amnh.org/vz/herpetology/5_6amphibia/?action=names&a_id=2443) ([1997](http://research.amnh.org/vz/herpetology/5_6amphibia/?action=names&year=1997)). A new subspecies of Common Skittering Frog, *Euphlyctis cyanophlyctis* (Schneider, 1799) from Balochistan, Pakistan. [Pakistan *Journal of Zoology*](http://research.amnh.org/vz/herpetology/5_6amphibia/?action=names&p_id=19), 29, 107-112.

Knowles, R. O. S. S., Mahony, M., Armstrong, J., & Donnellan, S. (2004). Systematics of sphagnum frogs of the genus Philoria (Anura: Myobatrachidae) in eastern Australia, with the description of two new species. *Records Australian Museum*, *56*(1), 57-74.

Krishna, S. N., & Krishna, S. B. (2005). Breeding population size of an endemic litter frog, *Clinotarsus curtipes*, in the Western Ghats, South India. *Herpetological Review*, *36*, 21.

Köhler, J. (2000). New species of eleutherodactylus (Anura: leptodactylidae) from cloud forest of bolivia. *Copeia*, *2000(2)*, 516–520. https://doi.org/10.1643/0045-8511(2000)000[0516:NSOEAL]2.0.CO;2

Köhler, J., Reichle, S. & Peters, G. (1997) Advertisement calls of three species of *Bufo* (Amphibia: Anura: Bufonidae) from lowland Bolivia. *Stuttgarter Beiträge zur Naturkunde Serie A (Biologie)*, 562, 1–8.

Köhler, J., Morales, V. R., Lötters, S., Reichle, S., & Aparicio, J. (1998). A new green species of frog, genus Eleutherodactylus, from Bolivia and Peru (Amphibia, Anura, Leptodactylidae). Studies on Neotropical Fauna and Environment. *Studies of Neotropical Fauna and Environment*, *33*, 93–99.

Köhler, J., & Lötter, S. (1999). Annotated list of amphibian records from the Departamento Pando, Bolivia, with description of some advertisement calls. *Bonner zoologische Beiträge : Herausgeber: Zoologisches Forschungsinstitut und Museum Alexander Koenig, Bonn.*, *48*, 259–273.

Köhler, J., Jungfer, K.-H., & Reichle, S. (2005a). Another new species of small hyla (Anura, hylidae) from amazonian sub-andean forest of western bolivia. *Journal of Herpetology*, *39*(1), 43–50. [https://doi.org/10.1670/0022-1511(2005)039[0043:ANSOSH]2.0.CO;2](about:blank)

Köhler, J., Scheelke, K., Schick, S., Veith, M., & Lotters, S. (2005b). Contribution to the taxonomy of hyperoliid frogs (Amphibia: Anura: Hyperoliidae): advertisement calls of twelve species from East and Central Africa. *African Zoology*, *40*(1), 127-142.

Köhler, J., Glaw, F. and Vences, M. (2008). Two additional treefrogs of the *Boophis ulftunni* species group (Anura: Mantellidae) discovered in rainforests of northern and south-eastern Madagascar. *Zootaxa* 1814: 37-48.

Köhler, J., Glaw, F., Pabijan, M., & Vences, M. (2015). Integrative taxonomic revision of mantellid frogs of the genus *Aglyptodactylus* (Anura: Mantellidae). *Zootaxa*, *4006*(3), 401-438.

Kok, P.J.R., MacCulloch, R.D., Gaucher, P., Poelman, E.H., Bourne, G.R., Lathrop, A., & Lenglet, G.L. (2006a) A new species of *Colostethus* (Anura, Dendrobatidae) from French Guiana with a redescription of *Colostethus beebei* (Noble, 1923) from its type locality. *Phyllomedusa*, *5*, 43–66.

Kok, P. J. R., Sambhu, H., Roopsind, I., Lenglet, G. L., & Bourne, G. R. (2006b). A new species of *Colostethus* (Anura: dendrobatidae) with maternal care from Kaieteur National Park, Guyana. *Zootaxa*, *1238*(1), 35–61. <https://doi.org/10.11646/zootaxa.1238.1.3>

Kok, P. J., Kokubum, M. N., MacCulloch, R. D., & Lathrop, A. (2007). Morphological variation in Leptodactylus lutzi (Anura, Leptodactylidae) with description of its advertisement call and notes on its courtship behavior. *Phyllomedusa*, *6*(1), 45-60.

Kok, P. J. R., Macculloch, R. D., Lathrop, A., Willaert, B., & Bossuyt, F. (2010). A new species of *Anomaloglossus* (Anura: aromobatidae) from the pakaraima mountains of guyana. *Zootaxa*, *2660*(1), 18–32.

Kolenc, F., C. Borteiro, D. Baldo, D. P. Ferraro, and C. Prigioni. (2009). The tadpoles and advertisement calls of *Pleurodema bibroni* Tschudi and *Pleurodema kriegi* (Müller), with notes on their geographic distribution and conservation status (Amphibia, Anura, Leiuperidae). *Zootaxa* 1969:1–35

Kraus, F. (2012). Identity of *Nyctimystes cheesmani* (Anura: Hylidae), with description of two new related species. Zootaxa 3493: 1–26.

Kraus, F., & Allison, A. (2002). A new species of Xenobatrachus (Anura: Microhylidae) from northern Papua New Guinea. *Herpetologica*, *58*(1), 56-66.

Kraus, F., & Allison, A. (2004). Two new treefrogs from normanby island, papua new guinea. *Journal of Herpetology*, *38*(2), 197–207. <https://doi.org/10.1670/100-03A>

Kraus, F., & Allison, A. (2006). Three new species of Cophixalus (Anura: Microhylidae) from southeastern New Guinea. *Herpetologica*, *62*(2), 202–220.

Kraus, F., and A. Allison. (2009). New microhylid frogs from the Muller Range, Papua New Guinea. *ZooKeys* 26: 53–76.

Krishna SN, Krishna SB (2006) Visual and acoustic communication in an endemic stream frog, *Micrixalus saxicolus* in the Western Ghats, India. *Amphibia-Reptilia* 27: 143–147.

Kubicki, B. (2007) *Ranas de Vidrio de Costa Rica / Glass Frogs of Costa Rica.* Editorial INBio, Santo Domingo de Heredia, 312 pp.

Kuramoto, M. (1980). Mating calls of treefrogs (Genus hyla) in the far east, with description of a new species from korea. *Copeia*, *1980*(1), 100–108. https://doi.org/10.2307/1444138

Kuramoto, M. (1986). Call structures of the rhacophorid frogs from taiwan. *Scientific Report of the Laboratory for Amphibian Biology* (Hiroshima University), *8*, 45–68. https://doi.org/10.15027/363

Kuramoto, M., & Wang, C.-S. (1987). A new rhacophorid treefrog from Taiwan, with comparisons to *Chirixalus eiffingeri* (Anura, Rhacophoridae). *Copeia*, *1987*(4), 931–942. https://doi.org/10.2307/1445556

Kuramoto, M., & Joshy, S. H. (2001). Advertisement call structures of frogs from southwestern India, with some ecological and taxonomic notes. *Current Herpetology*, *20*, 85–95.

Kuramoto, M., & Joshy, S. H. (2006). Morphological and acoustic comparisons of *Microhyla ornata*, *M*. *fissipes*, and *M*. *okinavensis* (Anura: Microhylidae). *Current Herpetology*, *25*(1), 15–27.

Kuramoto, M. and Joshy, S. H. (2009). Advertisement calls of Indian and Sri Lankan frogs. *Journal of the Bombay Natural History Society*, *105*, 14–18.

Kuramoto, M., & Dubois, A. (2009). Bioacoustic studies on three frog species from the Western Ghats, South India. *Current herpetology*, *28*(2), 65-70.

Kuramoto, M., Joshy, S. H., Kurabayashi, A., & Sumida, M. (2007). The genus Fejervarya (Anura: ranidae) in central western ghats, india, with descriptions of four new cryptic species. *Current Herpetology*, *26*(2), 81–105. https://doi.org/10.3105/1881-1019(2007)26[81:TGFARI]2.0.CO;2

Kuramoto, M., & Dubois, A. (2009). Bioacoustic studies on three frog species from the western ghats, south india. *Current Herpetology*, *28*(2), 65–70. https://doi.org/10.3105/018.028.0203

Kurniati, H., Sumadijaya, A., Boonman, A., & Laksono, W. T. (2010). Ecology, Distribution and Bio-acoustic of Amphibians in Degraded Habitat. Final report. Research Center for Biology. Indonesia. <https://www.researchgate.net/profile/Hellen_Kurniati/publication/279752784_Ecology_Distribution_and_Bio-acoustic_of_Amphibians_In_Degraded_Habitat/links/5599ea6a08ae99aa62cc74d6/Ecology-Distribution-and-Bio-acoustic-of-Amphibians-In-Degraded-Habitat.pdf>

Kurth, M., Hörnes, D., Esser, S., & Rödder, D. (2013). Notes on the acoustic repertoire of Melanophryniscus klappenbachi Prigioni & Langone, 2000. *Zootaxa*, *3626*(4), 597–600. https://doi.org/10.11646/zootaxa.3626.4.15

Kwet A. (2000). The genus *Pseudis* (Anura: Pseudidae) in Rio Grande do Sul, southern Brazil, with description of a new species. *Amphibia-Reptilia* 21: 39–55.

Kwet, A. (2007). Bioacoustic variation in the genus Adenomera in southern Brazil, with revalidation of *Leptodactylus nanus* Müller, 1922 (Anura, Leptodactylidae). *Zoosystematics and evolution*, *83*(S1), 56-68.

Kwet, A. & T. Miranda (2001). Zur Biologie und Taxonomie der Schwarzkröte *Melanophryniscus atroluteus* (Miranada-Ribeiro, 1925). *Herpetofauna*, *23*(134), 19–27.

Kwet, A., & Faivovich, J. (2001). Proceratophrys bigibbosa species group (Anura: Leptodactylidae), with description of a new species. *Copeia*, *2001*(1), 203-215.

Kwet, A., & Angulo, A. (2002). A new species of *Adenomera* (Anura, Leptodactylidae) from the Araucaria forest of Rio Grande do Sul (Brazil), with comments on the systematic status of southern populations of the genus. *Alytes*, *20*(1-2), 28-43.

Kwet, A., & Baldo, D. (2003). Advertisement call of the leptodactylid frog *Proceratophrys avelinoi*. *Amphibia Reptilia*, *24*(1), 104-108.

Kwet, A., & Solé, M. (2005). Validation of *Hylodes henselii* peters, 1870, from southern brazil and description of acoustic variation in eleutherodactylus guentheri(Anura: leptodactylidae). *Journal of Herpetology*, *39*(4), 521–532. <https://doi.org/10.1670/53-04A.1>

Kwet, A., and M. Solé. (2008). A new species of *Trachycephalus* (Anura: Hylidae) from the Atlantic Rain Forest in southern Brazil. *Zootaxa* 1947:53–67.

Kwet, A., R. Maneyro, D. Mebs & A. Zillikens (2005): Advertisement calls of *Melanophryniscus dorsalis* (Mertens, 1933) and *M. montevidensis* (Philippi, 1902), two parapatric species from southern Brazil and Uruguay, with comments on the *Melanophryniscus stelzneri* group (Anura: Bufonidae). *Salamandra*, *41*(1/2): 3–20.

Kwet, A., Di-Bernardo, M., & Maneyro, R. (2006). First record of *Chaunus achavali* (Anura, bufonidae) from Rio Grande do Sul, Brazil, with a key for the identification of the species in the *Chaunus marinus* group. *Iheringia. Série Zoologia*, *96*(4), 479–485. <https://doi.org/10.1590/S0073-47212006000400013>

Kwet, A., Steiner, J., & Zillikens, A. (2009). A new species of *Adenomera* (Amphibia: Anura: Leptodactylidae) from the Atlantic rain forest in Santa Catarina, southern Brazil. *Studies on Neotropical Fauna and Environment*, *44*(2), 93-107.

Lacerda, J. V. A., & Moura, M. R. (2013). Vocal repertoire of *Sphaenorhynchus palustris* (Anura, Hylidae), with notes on S. botocudo. *Salamandra*, *49*, 105-108.

La Marca, E. Coloma, L. A., Ron, S., Barrio-Amorós, C. L.. 2010. *Ctenophryne geayi*. The IUCN Red List of Threatened Species 2010: e.T57801A11683271. <http://dx.doi.org/10.2305/IUCN.UK.2010-2.RLTS.T57801A11683271.en>. Downloaded on 21 June 2018.

Langone, J. A. (1990). Revalidacion de *Hyla uruguaya* Schmidt, 1944 (amphibia, Anura, Hylidae). *Comunicaciones Zoológicas del Museo de Historia Natural de Montevideo* 12: 1–9.

Largen, M. J., M. Tandy & J. Tandy (1978) A new species of toad from the rift valley of ethiopia, with observations on the other species of *Bufo* (Amphibia, Anura, Bufonidae) recorded from this country, Monitore Zoologico Italiano. Supplemento, 10:1, 1-41, DOI: [10.1080/03749444.1978.10736856](https://doi.org/10.1080/03749444.1978.10736856)

Lascano, J.N. (2011). Description of the advertisement and distress call of *Chacophrys pierottii* and comments on the advertisement call of Lepidobatrachus llanensis(Anura: Ceratophryidae). *Journal of Natural History*, *45*, 2929–2938.

Leary, C. J. (2001). Evidence of convergent character displacement in release vocalizations of Bufo fowleri and Bufo terrestris (Anura; bufonidae). *Animal Behaviour*, *61*(2), 431–438. https://doi.org/10.1006/anbe.2000.1597

Leclair R., Jr, Leclair, M. H., Dubois, J., & Daoust, J.-L. (2000). Age and size of wood frogs, Rana sylvatica, from Kuujjuarapik, Northern Quebec. *Canadian Field-Naturalist*, *114*, 381–387.

Lehr, E., Lundberg, M., Aguilar, C., & May, R. von. (2006). New species of eleutherodactylus (Anura: leptodactylidae) from the eastern andes of central peru with comments on central peruvian eleutherodactylus. *Herpetological Monographs*, *20*, 105–128.

Leite, F. S. F., Pezzuti, T. L. & Garcia, P. C. A. (2012). A new species of the Bokermannohyla pseudopseudis group from the Espinhaço Range, Central Bahia, Brazil (Anura: Hylidae). *Herpetologica*, *68*, 401–409.

Leong, T. M., Matsui, M., Yong, H. S., & Hamid, A. A. (2003). Revalidation of Rana laterimaculata Barbour et Noble, 1916 from the synonymy of Rana baramica Boettger, 1901. *Current Herpetology*, *22*(1), 17–27. https://doi.org/10.5358/hsj.22.17

Lescure J., & Marty C. (2000). *Atlas des amphibiens de Guyane.* Muséum national d'Histoire naturelle, Paris, 392 p. (Patrimoines naturels ; 45).

Liang, Y.-s., & Wang, C.-s. (1978). A new tree frog Rhacophorus taipeianus (Anura: Rhacophoridae) from Taiwan (Formosa). *Quarterly Journal of the Taiwan Museum*, *31*, 185–202.

Liao, W. B., & Lu, X. (2011). Variation in body size, age and growth in the Omei treefrog (rhacophorus omeimontis) along an altitudinal gradient in western China. *Ethology Ecology & Evolution*, *23*(3), 248–261. <https://doi.org/10.1080/03949370.2011.567298>

Liem, D. S., & Ingram, G. J. (1977). Two new species of frogs (Anura: Myobatrachidae, Pelodryadidae) from Queensland and New South Wales. *Victorian Naturalist*, *94*(6), 255-62.

Lima, A. M. X., Garey, M. V., Noleto, R. B., & Verdade, V. K. (2010). Natural history of the lutz’s frog *Cycloramphus lutzorum* heyer, 1983 (Anura: cycloramphidae) in the brazilian atlantic forest: description of the advertisement call, tadpole, and karyotype. *Journal of Herpetology*, *44*(3), 360–371. <https://doi.org/10.1670/08-185.1>

Lima, A. P., Magnusson, W. E., Menin, M., Erdtmann, L. K., Rodrigues, D. J., Keller, C., & Hödl, W. (2005) Guia de Sapos da Reserva Adolpho Ducke: Amazônia Central. Manaus: Attema Design Editorial Ltda.

Lima, A. P., Sanchez, D. E. A., & Souza, J. R. D. (2007). A new amazonian species of the frog genus *Colostethus* (Dendrobatidae) that lays its eggs on undersides of leaves. *Copeia*, *2007*(1), 114–122. https://doi.org/10.1643/0045-8511(2007)7[114:ANASOT]2.0.CO;2

Lima, A. P., Erdtmann, L. K., & Amézquita, A. (2012). Advertisement call and colour in life of *Allobates crombiei* (Morales) “2000” [2002] (Anura: Aromobatidae) from the type Locality (Cachoeira do Espelho), Xingu River, Brazil. *Zootaxa*, *3475*(1), 86–88.

Lima, D. C., Borges-Nojosa, D. M., & Cechin, S. Z. (2014). The advertisement call of *Adelophryne maranguapensis* (Anura, eleutherodactylidae). *Zootaxa*, *3835*(2), 299–300. <https://doi.org/10.11646/zootaxa.3835.2.11>

Lima, L. P., R. P. Bastos, and A. A. Giaretta. (2004). A new *Scinax* Wagler, 1830 of the *S. rostratus* group from Central Brazil (Amphibia, Anura, Hylidae). Arquivos do Museu Nacional, Rio de Janeiro, 62 (4): 505- 512.

Lima, M.G., Lingnau, R. & Skuk, G.O. (2008) The advertisement call of Phyllodytes edelmoi (Anura, Hylidae). *South American Journal of Herpetology*, 3 (2), 118–12

Lima, T. G., Leite, J. R., Andrade, E., Araújo, K. C., & Weber, L. (2015). First record of Leptodactylus sertanejo (Anura: leptodactylidae: leptodactylinae) in the state of Maranhão, northeastern Brazil. *Check List*, *11(5)*, 1–4. https://doi.org/10.15560/11.5.1776

Lingnau, R. (2009). *Distribuição temporal, atividade reprodutiva e vocalizações em uma assembleia de anfíbios anuros de uma floresta ombrófila mista em Santa Catarina, sul do Brasil* (PhD Dissertation). Pontifícia Universidade Católica do Rio Grande do Sul (PUCRS), Porto Alegre. Recuperado de http://repositorio.pucrs.br:80/dspace/handle/10923/5413

Lingnau, R. And Bastos, R.P. (2003). Vocalizações de duas espécies de anuros do sul do Brasil (Amphibia: Hylidae). *Arquivos do Museu Nacional, Rio de Janeiro*, *61*(3), 203–207.

Lingnau, R., & Bastos, R. P. (2007). Vocalizations of the Brazilian torrent frog *Hylodes heyeri* (Anura: hylodidae): Repertoire and influence of air temperature on advertisement call variation. *Journal of Natural History*, *41*(17–20), 1227–1235. https://doi.org/10.1080/00222930701395626

Lingnau, R., Canedo, C. & Pombal, Jr., J.P. (2008a). A new species of *Hylodes* (Anura: Hylodidae) from the Brazilian Atlantic Forest. *Copeia* 2008(3): 595-602.

Lingnau, R., M. Solé, F. Dallacorte, and A. Kwet. (2008b). Description of the advertisement call of *Cycloramphus bolitoglossus* (Werner, 1897), with comments on other species in the genus from Santa Catarina, south Brazil (Amphibia, Cycloramphidae). *North-Western Journal of Zoology*, *4*, 224–235.

Lingnau, R., Zank, C., Colombo, P., & Kwet, A. (2013). Vocalization of Hylodes meridionalis (Mertens) (Anura, Hylodidae) in Rio Grande do Sul, Brazil, with comments on nocturnal calling in the family Hylodidae. *Studies on neotropical fauna and environment*, *48*(1), 76-80.

Littlejohn, M. J. (1958). A new species of frog of the genus *Crinia* Tschudi from South Eastern Australia. *Proceedings of the Linnean Society of New South Wales* 83: 222–226.

Littlejohn, M. J., & Main, A. R. (1959). Call structure in two genera of Australian burrowing frogs. *Copeia*, *1959*(3), 266-270.

Littlejohn, M. J., and A. A. Martin. ([1965](http://research.amnh.org/vz/herpetology/5_6amphibia/?action=names&year=1965)). A new species of *Crinia* (Anura: Leptodactylidae) from South Australia. [*Copeia*](http://research.amnh.org/vz/herpetology/5_6amphibia/?action=names&p_id=9) 1965: 319-324.

Littlejohn, M. J., & Harrison, P. A. (1985). The functional significance of the diphasic advertisement call of Geocrinia victoriana (Anura: Leptodactylidae). *Behavioral Ecology and Sociobiology*, *16*(4), 363-373.

Littlejohn, M. J., Watson, G. F., & Wright, J. R. (1993). Structure of advertisement call of *Litoria ewingi* (Anura: hylidae) introduced into new zealand from tasmania. *Copeia*, *1993*(1), 60–67. <https://doi.org/10.2307/1446295>

Littlejohn, M. J., & Wright, J. R. (1997). Structure of the acoustic signals of *Crinia glauerti* (Anura: Myobatrachidae) from south-western Australia, and comparison with those of C. signifera from South Australia. *Transactions of the Royal Society of South Australia*, *121*, 103-117.

Lizana, M., Márquez, R., & Martín-Sánchez, R. (1994). Reproductive biology of Pelobates cultripes (Anura: Pelobatidae) in central Spain. *Journal of Herpetology*, 19-27.

Lobo, F. (1993). Descripción de una nueva especie del género *Physalaemus* (Anura: Leptodactylidae) del Noroeste Argentino. Revista Española de Herpetología 7, 13-20.

Lobo, F. (1994). Descripción de una nueva especie de *Pseudopaludicola* (Anura: Leptodactylidae), redescripción de *P. falcipes* (Hensel, 1867) y P. saltica (Cope, 1887) y osteologia de las tres especies. *Cuadernos de Herpetología* 8(2):177-199.

Lötters, S., Glaw, F., Reichle, S., Köhler, J., & Meyer, E. (1999). Notes on vocalizations in three species of Atelopus from Central and South America. *Herpetozoa*, *12*, 79–83.

Lötters, S & J. Köhler (2000): Notes on *Cochranella nola* from Bolivia: advertisement call and distribution (Amphibia: Anura: Centrolenidae). *Herpetological Natural History*, *7*, 79–81.

Lötters, S., Haas, W., Schick, S., & Böhme, W. (2002a). On the systematics of the harlequin frogs (Amphibia: Bufonidae: Atelopus) from Amazonia. I: Description of a new species from the Cordillera Azul, Peru. *Salamandra*, *38*(2), 95–104.

Lotters, S., Haas, W., Schick, S., & Bohme, W. (2002b). On the systematics of the harlequin frogs (Amphibia: Bufonidae: Atelopus) from Amazonia. II: Redescription of Atelopus pulcher (Boulenger, 1882) from the eastern Andean versant in Peru. *Salamandra*, *38*(3), 165–184.

Lötters, S., A. Schmitz & S. Reichle (2005) A new cryptic species of poison frog from the Bolivian Yungas (Anura: Dendrobatidae: *Epipedobates*). *Herpetozoa*, *18*, 115–124.

Lourenço-de-Moraes, R., Solé, M. & Toledo, L.F. (2012) A new species of *Adelophryne* Hoogmoed & Lescure 1984 (Amphibia: Anura: Eleutherodactylidae) from the Atlantic rainforest of southern Bahia, Brazil. *Zootaxa*, *3441*, 59–68.

Lourenço-de-Moraes, R., Lantyer-Silva, A. S. F., Toledo, L. F., & Solé, M. (2013). Tadpole, oophagy, advertisement call, and geographic distribution of *Aparasphenodon arapapa* Pimenta, napoli and haddad 2009 (Anura, hylidae). *Journal of Herpetology*, *47*(4), 575–579. <https://doi.org/10.1670/11-326>

Lue, K.-Y., Lai, J.-S., & Chen, S.-L. (1994). A new species of rhacophorus (Anura: rhacophoridae) from taiwan. *Herpetologica*, *50*(3), 303–308.

Lugli, L., & Haddad, C. F. B. (2006). A new species of the Bokermannohyla pseudopseudis group from central bahia, brazil(Amphibia, hylidae). *Herpetologica*, *62*(4), 453–465. [https://doi.org/10.1655/0018-0831(2006)62[453:ANSOTB]2.0.CO;2](about:blank)

Lugli, L., & Haddad, C. F. B. (2006). New species of Bokermannohyla (Anura, hylidae) from central bahia, brazil. *Journal of Herpetology*, *40*(1), 7–15. https://doi.org/10.1670/67-05A.1

Lutz, B. (1954). Anfíbios anuros do distrito federal. *Memórias do Instituto Oswaldo Cruz*, *52*(1), 155–238. <https://doi.org/10.1590/S0074-02761954000100009>

Lutz, B. (1966). *Pithecopus ayeaye*, a new Brazilian hylid with vertical pupils and grasping feet. *Copeia* 1966: 236–240

Lutz, B. (1974). *Eleutherodactylus gualteri*, a new species from the organ mountains of brazil. *Journal of Herpetology*, *8*(4), 293–295. <https://doi.org/10.2307/1562897>

Lynch, J. D., & Hoogmoed, M. S. (1977). Two new species of *Eleutherocdactylus* (Amphibia : Leptodactylidae) from northeastern South America. *Proceedings of the Biological Society of Washington*, *90*, 424–439.

Lynch, J. D., & Myers, C. W. (1983). Frogs of the *fitzingeri* group of *Eleutherodactylus* in eastern Panama and chocoan South America (Leptodactylidae). *Bulletin of the American Museum of Natural History*, *175*(5). Recuperado de <http://digitallibrary.amnh.org/handle/2246/980>

Maeda, N., & Matsui, M. (1990). *Frogs and Toads of Japan*. Second Edition. Tokyo, Japan: Bun-ichi Sogo Shuppan Col., Ltd.

MacCulloch, R.D., Lathrop, A., Kok, P.J.R., Minter, L.R., Khan, S.Z., Barrio-Amorós, C.L. (2008a) A new species of *Adelophryne* (Anura: Eleutherodactylidae) from Guyana, with additional data on *A. gutturosa*. *Zootaxa,* 1884: 36–50.

MacCulloch, R. D., A. Lathrop, L. R. Minter, and S. Z. Khan. (2008b). *Otophryne* (Anura: Microhylidae) from the highlands of Guyana: redescriptions, vocalizations, tadpoles and new distributions. *Papéis Avulsos de Zoologia*, 48: 247–261

Magalhães, F. de M., Juncá, F. A., & Garda, A. A. (2015). Tadpole and vocalisations of *Phyllodytes wuchereri* (Anura: Hylidae) from Bahia, Brazil. *Salamandra*, *51*(2), 83-90

Magrini, L., Facure, K. G., Giaretta, A. A., da Silva, W.R., & Costa, R. C. (2010). Geographic call variation and further notes on habitat of *Ameerega flavopicta* (Anura, dendrobatidae). *Studies on Neotropical Fauna and Environment*, *45*(2), 89–94. <https://doi.org/10.1080/01650521.2010.494025>

Magrini, L., Carvalho-e-Silva, S. D., Beda, A. F., & Giaretta, A. A. (2011). Calls of five species of the *Scinax ruber* (Anura: Hylidae) clade from Brazil with comments on their taxonomy. *Zootaxa*, *3066*, 37-51.

Malkmus, R. (1996). Leptobrachium gunungensis sp. n. (Anura: Pelobatidae) vom Mount Kinabalu, Nord‐Borneo. *Zoosystematics and Evolution*, *72*(2), 297-301.

Malkmus, R., and M. Matsui. (1997). *Megophrys kobayashii*, ein neuer pelobatider Frosch vom Mount Kinabalu. *Sauria* 19: 31–37.

Malkmus, R., Manthey, J. U., Vogel, G., Hoffmann, P., & Kosuch, J. (2002). *The amphibians and reptiles of Mount Kinabalu*. Ruggell: Gantner, A R.

Manamendra-Arachchi, K., & Pethiyagoda, R. (2001a). Polypedates fastigo, a new tree frog (Ranidae: Rhacophorinae) from Sri Lanka. *Journal of South Asian Natural History*. Colombo *5*, 191–199.

Manamendra-Arachchi, K., & Pethiyagoda, R. (2001b). Ramanella nagaoi, a new tree-hole frog (Microhylidae) from southern Sri Lanka. *Journal of South Asian Natural History*, *5*(2), 121-133.

Manamendra-Arachchi, K., & Pethiyagoda, R. (2005). The Sri Lankan shrub-frogs of the genus Philautus Gistel, 1848 (Ranidae: Rhacophorinae), with description of 27 new species. Contributions to Biodiversity Exploration and Research in Sri Lanka. *Raffles Bulletin of Zoology. Supplement*, *12*, 163–303.

Maneyro, R., Núñez, D., Borteiro, C., Tedros, M., & Kolenc, F. (2008). Advertisement call and female sexual cycle in Uruguayan populations of *Physalaemus henselii* (Anura, Leiuperidae). *Iheringia. Série Zoologia*, *98*(2), 210-214.

Mângia, S., Santana, D. J., & Feio, R. N. (2010). Advertisement call of the cycloramphid toad *Proceratophrys melanopogon* (Miranda-Ribeiro, 1926). *South American Journal of Herpetology*, *5*(2), 127-131.

Márquez, R. (1995). Female choice in the midwife toads (*Alytes obstetricans* and *A. cisternasii*). *Behaviour*, *132*(1), 151–161. <https://doi.org/10.1163/156853995X00342>

Marquez, R., De la Riva, I., & Bosch, J. (1993). Advertisement calls of bolivian species of Hyla (Amphibia, anura, hylidae). *Biotropica*, *25*(4), 426–443. <https://doi.org/10.2307/2388866>

Márquez, R., Riva, I., & Bosch, J. (1995). Advertisement calls of Bolivian Leptodactylidae (Amphibia, Anura). *Journal of Zoology*, *237*(2), 313-336.

Márquez, R., De la Riva, I., Bosch, J., & Matheu, E. (2002). Guía Sonora De Las Ranas Y Sapos De Bolivia/Sounds Of Frogs And Toads Of Bolivia. Alosa, sons de la natura y Fonoteca Zoologica: Barcelona.

Márquez, R., Moreira, C., Amaral, J. P. S. do, Pargana, J. M., & Crespo, E. G. (2005). Sound pressure level of advertisement calls of *Hyla meridionalis* and *Hyla arborea*. *Amphibia-Reptilia*, *26*(3), 391–395. https://doi.org/10.1163/156853805774408603

Márquez, R., & Bosch, J. (2009). Advertisement calls of the midwife toads Alytes (Amphibia, anura, discoglossidae) in continental Spain. *Journal of Zoological Systematics and Evolutionary Research*, *33*(3–4), 185–192. <https://doi.org/10.1111/j.1439-0469.1995.tb00971.x>

Martin, A. A. (1972). Studies in Australian amphibia III. The *Limnodynastes dorsalis* complex (Anura: Leptodactylidae). *Australian Journal of Zoology*, *20*(2), 165-211.

Martin, A. A., Martin, A. A., Watson, G. F., Gartside, D. F., Littlejohn, M. J., & Loftus-Hills, J. J. (1979). A new species of the Litoria peroni complex (Anura: hylidae) from eastern Australia. *Proceedings of the Linnean Society of New South Wales.*, *103*, 23–35.

Martin, A. A., M. J. Tyler, and M. M. Davies. (1980). A new species of *Ranidella* (Anura: Leptodactylidae) from northwestern Australia. *Copeia* 1980: 93–99.

Martin, A. A., & Littlejohn, M. J. (1982). *Tasmanian amphibians*. Hobart: Fauna of Tasmania Committee, University of Tasmania.

Martins, M. and A. J. Cardoso. (1987). Novas espécies de hilídeos do Estado do Acre (Amphibia: Anura). *Revista Brasileira de Biologia*, *47*, 549–558

Martins, I. A., & Jim, J. (2004). Advertisement call of *Hyla jimi* and *Hyla elianeae* (Anura, hylidae) in the botucatu region, são paulo, brazil. *Brazilian Journal of Biology*, *64*(3b), 645–654. <https://doi.org/10.1590/S1519-69842004000400012>

Martins, L. B., Silva, W. R., & Giaretta, A. A. (2009). Distribution and calls of two South American frogs (Anura). *Salamandra*, *45*, 106–109.

Martins, L. B., & Giaretta, A. A. (2011). A new species of *Proceratophrys* Miranda-Ribeiro (Amphibia: Anura: Cycloramphidae) from central Brazil. *Zootaxa*, *2880*(1), 41-50.

Martins, L. B., & Giaretta, A. A. (2012). Advertisement calls of two species of *Proceratophrys* (Anura: Odontophrynidae) from Minas Gerais, Brazil, with comments on their distribution, taxonomy and conservation status. *South American Journal of herpetology*, *7*(3), 203-212.

Martins, L. B., & Giaretta, A. A. (2013). Morphological and acoustic characterization of *Proceratophrys goyana* (Lissamphibia: Anura: Odontophrynidae), with the description of a sympatric and related new species. *Zootaxa*, *3750*(4), 301-320.

Martins, I. A., & Haddad, C. F. B. (2010). A new species of ischnocnema from highlands of the atlantic forest, southeastern Brazil (Terrarana, brachycephalidae). *Zootaxa*, *2617*, 55–65. <https://doi.org/10.5281/zenodo.197955>

[Marty C., Lebailly M., Gaucher P., Tostain O., Dewynter M., Blanc M. & Fouquet A. 2013. Report on the occurrence of *Dendropsophus leali* (Bokermann, 1964) (Anura; Hylidae) in French Guiana. *Bull. Soc. Herp. Fr.*, *148*, 419–424.](https://antoinefouquet.files.wordpress.com/2012/09/marty-et-al-2013.pdf)

Matsui, M. (1982). Call Characteristics of Several Anuran Species from East Kalimantani. *Contributions from the Biological Laboratory, Kyoto University*, *26*, 131–139.

Matsui, M. (1995). Calls produced by a “voiceless” frog, *Rana blythi* Boulenger 1920, from Peninsular Malaysia (Amphibia: Anura). *Tropical Zoology*, *8*(2), 325–331. <https://doi.org/10.1080/03946975.1995.10539289>

Matsui, M. (1994). A taxonomic study of the *Rana narina* complex, with description of three new species (Amphibia: Ranidae). *Zoological Journal of the Linnean Society*, *111*, 385–415.

Matsui, M. (2009) A new species of *Kalophrynus* with a unique male humeral spine from Peninsular Malaysia (Amphibia, Anura, Microhylidae). *Zoological Science*, 26, 579–585.

Matsui, M. (2009b). A new species of *Philautus* (Amphibia, Anura, Rhacophoridae) from the lowland of Sarawak, western Borneo. *Zoological Science*, *26*(6), 437–442. https://doi.org/10.2108/zsj.26.437

Matsui, M., Nishikawa, K., Belabut, D. M., Norhayati, A., & Yong, H. S. (2012). A new species of *Kalophrynus* (Amphibia, Anura, Microhylidae) from Southern Peninsular Malaysia. *Zootaxa*, *3155*, 38–46.

Matsui, M., Wu, G.-F., & Yong, H. S. (1993). Acoustic characteristics of three species of the genus *Amolops* (Amphibia, Anura, Ranidae). *Zoological Science*, 10, 691–695.

Matsui, M., & Wu, G.-F. (1994). Acoustic characteristics of treefrogs from Sichuan, China, with comments on systematic relationship of *Polypedates* and *Rhacophorus* (Anura, Rhacophoridae). *Zoological Science*, *11*, 495–490.

Matsui, M., Chen, S.-L., & Lue, K.-Y. (1997). Advertisement call characteristics of a taiwanese green treefrog *Rhacophorus prasinatus*. *Bonner zoologische Beiträge*, *47*(1-2), 165–174.

Matsui, M., & Nabhitabhata, J. (2006). A new species of *Amolops* from thailand (Amphibia, Anura, Ranidae). *Zoological Science*, *23*(8), 727–732. https://doi.org/10.2108/zsj.23.727

Matsui, M., Toda, M., & Ota, H. (2007). A new species of frog allied to *Fejervarya limnocharis* from the Southern Ryukyus, Japan (Amphibia: ranidae). *Current Herpetology*, *26*(2), 65–79. [https://doi.org/10.3105/1881-1019(2007)26[65:ANSOFA]2.0.CO;2](about:blank)

McLeod, D. S., and N. Ahmad. 2007. A new species of Theloderma (Anura: Rhacophoridae) from southern Thailand and peninsular Malaysia. *Russian Journal of Herpetology*, *14*, 65–72.

McLister, J. D., Lougheed, S. C., & Bogart, J. P. (1991). Electrophoretic and vocalization comparisons among three leptodactylid frogs (Pleurodema spp.) from northwestern Argentina. *Canadian Journal of Zoology*, *69*(9), 2397-2403.

Meegaskumbura, M., & Manamendra-Arachchi, K. (2005). Description of eight new species of shrubfrogs (Ranidae: Rhacophorinae: Philautus) from Sri Lanka. Contributions to Biodiversity Exploration and Research in Sri Lanka. *Raffles Bulletin of Zoology, Supplement*, *12*, 305–338.

Melo-Sampaio P. R., De Souza, M. B., & Peloso, P. L. V. (2013). A new, riparian species of *Allobates* Zimmermann and Zimmermann, 1988 (Anura: Aromobatidae) from southwestern Amazonia. *Zootaxa*, *3716*, 336-348.

Mendelson, J. R. I., Williams, B. L., Sheil, C. A., & Mulcahy, D. G. (2005). Systematics of the *Bufo coccifer* complex (Anura: bufonidae) of mesoamerica. *Scientific Papers*, *38*, 1–27. <https://doi.org/10.5962/bhl.title.8482>

Mendes, C. V. D. M., JUNIOR, E. M., Ruas, D. S., Oliveira, R. M., & Solé, M. (2013). Advertisement call of Scinax strigilatus (Spix, 1824) (Anura: Hylidae) from southern Bahia, Brazil. *Zootaxa*, *3647*(3), 499-500.

Menegon, M., Salvidio, S., & Loader, S. P. (2004). Five new species of *Nectophrynoides* Noble 1926 (Amphibia, Anura, Bufonidae) from the Eastern Arc Mountains, Tanzania. *Tropical Zoology*, *17*(1), 97–121. https://doi.org/10.1080/03946975.2004.10531201

Menegon, M., Salvidio, S., Ngalason, W., & Loader, S. P. (2007). A new dwarf forest toad (Amphibia: Bufonidae: Nectophrynoides) from the Ukaguru Mountains, Tanzania. *Zootaxa*, *1541*(1), 31–40. <https://doi.org/10.11646/zootaxa.1541.1.3>

Menin, M., Silva, R. A., & Giaretta, A. A. (2004). Reproductive biology of *Hyla goiana* (Anura, hylidae). *Iheringia. Série Zoologia*, *94*(1), 49–52. <https://doi.org/10.1590/S0073-47212004000100008>

Menzies, J. I. (1993) Systematics of *Litoria iris* (Anura: Hylidae) and its allies in New Guinea and a note on sexual dimorphism in the group. *Australian Journal of Zoology* 41:225–255.

Menzies, J. I. (2014). Notes on *Nyctimystes* (Anura: Hylidae), tree frogs of New Guinea, with descriptions of four new species. *Alytes* 30: 42–68.

Menzies, J. I. and R. G. Zweifel. (1974). Systematics of *Litoria arfakiana* of New Guinea and sibling species (Salientia, Hylidae). *American Museum Novitates* 2558:1–16

Menzies, J. I. and M. J. Tyler. (2004). *Litoria gracilenta* (Anura: Hylidae) and related species in New Guinea. *Australian Journal of Zoology* 52:191–214

Mercurio, V. (2009). Advertisement calls of three species of *Arthroleptis* (Anura: Arthroleptidae) from Malawi. *Journal of Herpetology*, *43*(2), 345–350. <https://doi.org/10.1670/08-119R1.1>

Milstead, W. W. (1960). Frogs of the genus *Physalaemus* in southern Brazil with the description of a new species. *Copeia*, *1960*(2), 83-89.

Minton, S. A. (1966). A contribution to the herpetology of West Pakistan. Bulletin of the AMNH ; v. 134, article 2. Recuperado de <http://digitallibrary.amnh.org/handle/2246/1129>

Monnet, J.-M., & Cherry, M. I. (2002). Sexual size dimorphism in anurans. *Proceedings of the Royal Society B: Biological Sciences*, *269*(1507), 2301–2307. https://doi.org/10.1098/rspb.2002.2170

Morais, A. R., & Kwet, A. (2012). Description of the advertisement call of *Physalaemus lisei* (Anura: Leiuperidae). *Salamandra*, *48*(4), 227-229.

Morais, A. R., Batista, V. G., Gambale, P. G., Signorelli, L., & Bastos, R. P. (2012). Acoustic communication in a Neotropical frog (Dendropsophus minutus): vocal repertoire, variability and individual discrimination. *Herpetological Journal*, *22*(4), 249–257.

Morales, V. R. (2017). Una especie nueva de Eleutherodactylus (Amphibia; anura; brachycephalidae) de la Amazonía central del Perú. *Biotempo*, *7*, 5–11.

Moravec, J., Aparicio, J., & Köhler, J. (2006). A new species of tree frog, genus *Dendropsophus* (Anura: hylidae), from the Amazon of northern Bolivia. *Zootaxa*, *1327*(1), 23–40. https://doi.org/10.11646/zootaxa.1327.1.2

Mou, Y. P., Risch, J. P., & Lue, K. Y. (1983). Rhacophorus prasinatus, a new tree frog from Taiwan, China (Amphibia, Anura, Rhacophoridae). *Alytes*, 154–162.

Moura, M.R., Lacerda, J.V.A. & Feio, R.N. (2012) Advertisement call and distribution of *Dendropsophus berthalutzae* (Anura: Hylidae). *Salamandra*, *48*, 177–180.

Myers, C. W., & Daly, J. W. (1979). A name for the poison frog of Cordillera Azul, eastern Peru, with notes on its biology and skin toxins (Dendrobatidae). American Museum novitates ; no. 2674. Recuperado de <http://digitallibrary.amnh.org/handle/2246/5326>

Nali, R. C., & Prado, C. P. A. (2014). Complex call with different messages in *Bokermannohyla ibitiguara* (Anura, hylidae), a gladiator frog of the brazilian cerrado. *Journal of Herpetology*, *48*(3), 407–414. <https://doi.org/10.1670/13-090>

Nali, R. C., Borges, M. M., & Prado, C. (2015). Advertisement and release calls of *Phyllomedusa ayeaye* (Anura: Hylidae) with comments on the social context of emission. *Zoologia*, *32*(4), 263-269.

Napoli, M.F. & Pimenta, B.V.S. (2003) Nova espécie do grupo de *Hyla circumdata* (Cope, 1870) do sul da Bahia, Brasil (Amphibia, Anura, Hylidae). *Arquivos do Museu Nacional*, *61*, 189–194.

Napoli, M. F., & Caramaschi, U. (2004). Two new species of the *Hyla circumdata* group from serra do mar and serra da mantiqueira, southeastern brazil, with description of the advertisement call of *Hyla ibitipoca* (Anura, hylidae). *Copeia*, *2004*(3), 534–545. https://doi.org/10.1643/CH-02-079R1

Napoli, M. F., & Juncá, F. A. (2006). A new species of the Bokermannohyla circumdata group (Amphibia: anura: hylidae) from chapada diamantina, state of bahia, brazil. *Zootaxa*, *1244*(1), 57–68. <https://doi.org/10.11646/zootaxa.1244.1.4>

Napoli, M. F., & Pimenta, B. V. S. (2009). A new species of the *Bokermannohyla circumdata* group (Anura: hylidae) from the coastal forests of bahia, northeastern brazil. *Copeia*, *2009*(4), 674–683. <https://doi.org/10.1643/CH-08-224>

Napoli, M. F., Abreu, R. O. D., Cruz, D., Herrera, J. B., Petersen, E., & Klein, W. (2014). Advertisement call of *Dendropsophus studerae* (Carvalho-e-silva, carvalho-e-silva and izecksohn, 2003) (Anura: hylidae), with new record and geographic distribution extension. *Zootaxa*, *3878*(6), 593–596. https://doi.org/10.11646/zootaxa.3878.6.6

Narins, P. M., & Smith, S. L. (1986). Clinal variation in anuran advertisement calls: basis for acoustic isolation? *Behavioral Ecology and Sociobiology*, *19*(2), 135–141. https://doi.org/10.1007/BF00299948

Narvaes, P., & Rodrigues, M. T. (2009). Taxonomic revision of *Rhinella granulosa* species group (Amphibia, anura, bufonidae), with a description of a new species. *Arquivos de Zoologia*, *40*(1), 1–73. <https://doi.org/10.11606/issn.2176-7793.v40i1p1-73>

Nascimento, L. B., Pombal Jr, J. P., & Haddad, C. F. (2001). A new frog of the genus *Hylodes* (Amphibia: Leptodactylidae) from Minas Gerais, Brazil. *Journal of Zoology*, *254*(4), 421-428.

Nascimento, L. B., R. N. Feio, and C. A. G. Cruz. (2005). A new species of diurnal frog in the genus *Crossodactylus* Duméril and Bibron, 1841 (Anura, Leptodactylidae) from southeastern Brazil. *Amphibia-Reptilia* 26:497–505

Neckel-Oliveira, S., Galatti, U., Faveri, S. B., Albareli, L. P., & Nascimento, H. E. M. (2013). Ecological correlates in Brazilian Amazonian anurans: implications for conservation. *Amphibia-Reptilia*, *34*(2), 217–232. <https://doi.org/10.1163/15685381-00002890>

Nelson, C. E. (1973). Mating calls of the Microhylinae: descriptions and phylogenetic and ecological considerations. *Herpetologica*, 163-176.

Ngo, B. V., & Ngo, C. D. (2013). Reproductive activity and advertisement calls of the asian common toad *Duttaphrynus melanostictus* (Amphibia, Anura, Bufonidae) from bach ma national park, vietnam. *Zoological Studies*, *52*(1), 12. <https://doi.org/10.1186/1810-522X-52-12>

Nunes, I., & Juncá, F. A. (2006). Advertisement calls of three leptodactylid frogs in the state of Bahia, northeastern Brazil (Amphibia, Anura, Leptodactylidae), with considerations on their taxonomic status. *Arquivos do Museu Nacional*, *64*(2), 151-157.

Nunes, I., Santiago, R. S., & Juncá, F. A. (2007). Advertisement calls of four hylid frogs from the state of bahia, northeastern Brazil (Amphibia, anura, hylidae). *South American Journal of Herpetology*, *2*(2), 89–96. [https://doi.org/10.2994/1808-9798(2007)2[89:ACOFHF]2.0.CO;2](about:blank)

Nunes, I., & Pombal Jr, J. P. (2010). A new *Scinax* Wagler (Amphibia, Anura, Hylidae) from the Atlantic rain forest remains of southern State of Bahia, north-eastern Brazil. *Amphibia-Reptilia*, *31*(3), 347-353.

Nunes, I., Carvalho Jr, R. D., & Pereira, E. G. (2010). A new species of *Scinax* Wagler (Anura: Hylidae) from Cerrado of Brazil. *Zootaxa*, *2514*(1), 24-34.

Nunes, I., Kwet, A., & Pombal, J. P. (2012). Taxonomic revision of the *Scinax alter* species complex (Anura: Hylidae). *Copeia*, *2012*(3), 554-569.

Nunes, I., Loebmann, D., Cruz, C. A. G., & Haddad, C. F. (2015). Advertisement call, colour variation, natural history, and geographic distribution of Proceratophrys caramaschii (Anura: Odontophrynidae). *Salamandra*, *51*(2), 103-110.

Nunes-de-Almeida, C. H. L., Assis, C. L., Feio, R. N., & Toledo, L. F. (2016). Redescription of the advertisement call of five species of *Thoropa* (Anura, cycloramphidae), including recordings of rare and endangered species. *PLOS ONE*, *11*(9), e0162617. https://doi.org/10.1371/journal.pone.0162617

Nunez, J. J., Rabanal, F. E., & Formas, J. R. (2012). Description of a new species of *Eupsophus* (Amphibia: neobatrachia) from the Valdivian Coastal range, Southern Chile: an integrative taxonomic approach. *Zootaxa*, *3305*(1), 53–68. <https://doi.org/10.11646/zootaxa.3305.1.3>

Ohler, A., & Delorme, M. (2006). Well known does not mean well studied: morphological and molecular support for existence of sibling species in the Javanese gliding frog *Rhacophorus reinwardtii* (Amphibia, anura). *Comptes Rendus Biologies*, *329*(2), 86–97. <https://doi.org/10.1016/j.crvi.2005.11.001>

Ohler, A., Wollenberg, K. C., Grosjean, S., Hendrix, R., Vences, M., Ziegler, T., & Dubois, A. (2011). Sorting out Lalos: description of new species and additional taxonomic data on megophryid frogs from northern Indochina (genus Leptolalax, Megophryidae, Anura). *Zootaxa*, *3147*, 1-83.

Ohler, A., & Frétey, T. (2014). Going back to rovuma: the frog fauna of a coastal dry forest, and a checklist of the amphibians of Mozambique. *Journal of East African Natural History*, *103*(2), 73–124. <https://doi.org/10.2982/028.103.0203>

Ohmer, M. E., Robertson, J. M., & Zamudio, K. R. (2009). Discordance in body size, colour pattern, and advertisement call across genetically distinct populations in a Neotropical anuran (*Dendropsophus ebraccatus*). *Biological Journal of the Linnean Society*, *97*(2), 298–313. <https://doi.org/10.1111/j.1095-8312.2009.01210.x>

Oliver, P., Richards, S. J., Tjaturadi, B., & Iskandar, D. (2007). A new large green species of Litoria (Anura: Hylidae) from western New Guinea. *Zootaxa*, *1519*(1), 17-26.

Oliveira, M. E., Paillette, M., Rosa, H. D., & Crespo, E. G. (1991). A natural hybrid between Hyla arborea and Hyla meridionalis detected by mating calls. *Amphibia-Reptilia*, *12*(1), 15–20. <https://doi.org/10.1163/156853891X00284>

Oliveira, F. F. R., Nascimento, L. B., Eterovick, P. C., & Sazima, I. (2013). Description of the Tadpole and Redescription of the Advertisement Call of Physalaemus evangelistai (Anura, Leiuperidae), with Notes on Its Natural History. *Journal of Herpetology*, *47*(4), 539-543.

Oliveira Filho, J. C. D., & Giaretta, A. A. (2006). Tadpole and advertisement call of *Chiasmocleis albopunctata* (Anura, Microhylidae) from Brazil. *Zootaxa*, *1353*, 63-68.

Oliveira Filho, J. C. D., & Giaretta, A. A. (2008). Reproductive behavior of Leptodactylus mystacinus (Anura, Leptodactylidae) with notes on courtship call of other Leptodactylus species. *Iheringia. Série Zoologia*, *98*(4), 508-515.

Opazo, D., Velásquez, N., Veloso, A., & Penna, M. (2009). Frequency-modulated vocalizations of eupsophus queulensis(Anura: cycloramphidae). *Journal of Herpetology*, *43*(4), 657–664. <https://doi.org/10.1670/08-189.1>

Orrico, V. G. D., Carvalho-e-Silva, A. M. P. T., & Carvalho-e-Silva, S. P. (2006). Redescription of the advertisement call of Aplastodiscus arildae (Cruz & peixoto) and description of the call of Aplastodiscus weygoldti (Cruz & peixoto) with general notes about the genus in Southeastern Brazil (Anura, hylidae). *Revista Brasileira de Zoologia*, *23*(4), 994–1001. https://doi.org/10.1590/S0101-81752006000400003

Ortega-Andrade, H. M., Rojas-Soto, O., & Paucar, C. (2013). Novel data on the ecology of *Cochranella mache* (Anura: centrolenidae) and the importance of protected areas for this critically endangered glassfrog in the neotropics. *PLoS ONE*, *8*(12), e81837. <https://doi.org/10.1371/journal.pone.0081837>

Ortega-Andrade, H. M., & Ron, S. R. (2013). A new species of small tree frog, genus Dendropsophus (Anura: hylidae) from the eastern Amazon lowlands of Ecuador. *Zootaxa*, *3652*(1), 163–178. https://doi.org/10.11646/zootaxa.3652.1.6

Ortiz, J. C., Ortiz, J. C., Ibarra-Vidal, H., & Formas, J. R. (1989). A new species of *Eupsophus* (Anura: leptodactylidae) from Contulmo, Nahuelbuta range, southern Chile. *Proceedings of The Biological Society of Washington*, *102*, 1031–1035.

Ospina, O. E., Villanueva-Rivera, L. J., Corrada-Bravo, C. J., & Aide, T. M. (2013). Variable response of anuran calling activity to daily precipitation and temperature: implications for climate change. *Ecosphere*, *4*(4), art47. https://doi.org/10.1890/ES12-00258.1

Ovaska, K. E., & Caldbeck, J. (1997). Courtship behavior and vocalizations of the frogs *Eleutherodactylus antillensis* and E. cochranae on the british virgin islands. *Journal of Herpetology*, *31*(1), 149–155. <https://doi.org/10.2307/1565348>

Pabijan, M., Gehring, P. S., Koehler, J., Glaw, F., & Vences, M. (2011). A new microendemic frog species of the genus Blommersia (Anura: Mantellidae) from the east coast of Madagascar. *Zootaxa*, *2978*(1), 34-50.

Padial, J. M., Gonzáles, L., & De la Riva, I. (2005). A new species of the eleutherodactylus discoidalis group (Anura: leptodactylidae) from andean humid montane forests of bolivia. *Herpetologica*, *61*(3), 318–325.

Padial, J. M., & De la Riva, I. (2005). Rediscovery, redescription, and advertisement call of eleutherodactylus heterodactylus (Miranda Ribeiro, 1937) (Anura: leptodactylidae), and notes on other eleutherodactylus. *Journal of Herpetology*, *39*(3), 372–379. https://doi.org/10.1670/191-04A.1

Padial, J. M., Chaparro, J. C., & Riva, I. D. L. (2008). Systematics of Oreobates and the Eleutherodactylus discoidalis species group (Amphibia, anura), based on two mitochondrial DNA genes and external morphology. *Zoological Journal of the Linnean Society*, *152*(4), 737–773. https://doi.org/10.1111/j.1096-3642.2007.00372.x

Padial, J. M., & De La Riva, I. (2009). Integrative taxonomy reveals cryptic Amazonian species of Pristimantis (Anura: strabomantidae). *Zoological Journal of the Linnean Society*, *155(1)*, 97–122. https://doi.org/10.1111/j.1096-3642.2008.00424.x

Pansonato, A., Ávila, R.W., Kawashita-­Ribeiro, R.A., Morais,D.H. (2011). Advertisement call and new distribution records of *Hypsiboas leucocheilus* (Anura: Hylidae). *Salamandra* 47: 55-­58.

Pansonato, A., D.H. Morais, R.W. Ávila, R.A. Kawashita-Ribeiro, C. Strüssmann, and I.A. Martins. 2012. A new species of *Pseudopaludicola* Miranda-Ribeiro, 1926 (Anura: Leiuperidae) from the state of Mato Grosso, Brazil, with comments on the geographic distribution of *Pseudopaludicola canga* Giaretta & Kokubum, 2003. Zootaxa 3523:49–58.

Pansonato, A., Strüssmann, C., Mudrek, J. R., & Martins, I. A. (2013). Morphometric and bioacoustic data on three species of *Pseudopaludicola* Miranda-Ribeiro, 1926 (Anura: Leptodactylidae: Leiuperinae) described from Chapada dos Guimarães, Mato Grosso, Brazil, with the revalidation of Pseudopaludicola ameghini (Cope, 1887). *Zootaxa*, *3620*(1), 147-162.

Parker, H.W. (1934):A monograph of the frogs of the family Microhylidae. Trustees of the British Museum, 1-208.

Parker, H. W. (1936). The amphibians of the mamfe division, cameroons. –i. Zoogeography and systematics. *Proceedings of the Zoological Society of London*, *106*(1), 135–163. <https://doi.org/10.1111/j.1096-3642.1936.tb02284.x>

Passmore, N. I., & Carruthers, V. C. (1975). A new species of *Tomopterna* (Anura: Ranidae) from the Kruger National Park, with notes on related species. *Koedoe. Pretoria*, *18*, 31–50.

Pavan, D., Narvaes, P., & Rodrigues, M. T. (2001). A new species of leptodactlid frog from the Atlantic forests of southeastern Brazil with notes on the status and on the speciation of *Hylodes* species groups. *Papéis Avulsos de Zoologia* 41: 407–425.

Peloso, P. L. V., & Sturaro, M. J. (2008). A new species of narrow-mouthed frog of the genus *Chiasmocleis* Méhelÿ 1904 (Anura, Microhylidae) from the Amazonian rainforest of Brazil. *Zootaxa*, *1947*, 39-52.

Peloso, P. L., Sturaro, M. J., Forlani, M. C., Gaucher, P., Motta, A. P., & Wheeler, W. C. (2014). Phylogeny, taxonomic revision, and character evolution of the genera Chiasmocleis and Syncope (Anura, Microhylidae) in Amazonia, with descriptions of three new species. *Bulletin of the American Museum of Natural History*, *386*(1), 1-112.

Pengilley, R. K. (1971). Calling and associated behaviour of some species of Pseudophryne (Anura: Leptodactylidae). *Journal of Zoology*, *163*(1), 73-92.

Penna, M., & Meier, A. (2011). Vocal strategies in confronting interfering sounds by a frog from the southern temperate forest, batrachyla antartandica: frog vocal strategies under interference. *Ethology*, *117*(12), 1147–1157. https://doi.org/10.1111/j.1439-0310.2011.01973.x

Penna, M., & Solís, R. (1998). Frog call intensities and sound propagation in the South American temperate forest region. *Behavioral Ecology and Sociobiology*, *42*(6), 371–381. https://doi.org/10.1007/s002650050452

Penna, M., & Veloso, A. (1990). Vocal diversity in frogs of the south american temperate forest. *Journal of Herpetology*, *24*(1), 23–33. <https://doi.org/10.2307/1564285>

Pereira, E. G., & Nascimento, L. B. (2004). Descrição da vocalização e do girino de Pseudopaludicola mineira Lobo, 1994, com notas sobre a morfologia de adultos (Amphibia, Anura, Leptodactylidae). *Arquivos do Museu Nacional*, *62*(3), 233-240.

Pereyra, M. O., Borteiro, C., Baldo, D., Kolenc, F., & Conte, C. E. (2012). Advertisement call of the closely related species *Scinax aromothyella* Faivovich 2005 and S. berthae (Barrio 1962), with comments on the complex calls in the S. catharinae groups. *Herpetological Journal*, *22*(2), 133-137.

Peres, J., & Simon, J. E. (2012). Physalaemus maximus Feio, Pombal Jr. and Caramaschi, 1999 (Anura: Leiuperidae): distribution extension and advertisement call. *Check List*, *8*(3), 507-509.

Perez-Peña, P. E., Chavez, G., Twomey, E., & Brown, J. L. (2010). Two new species of Ranitomeya (Anura: dendrobatidae) from eastern Amazonian Peru. *Zootaxa*, *2439*(1), 1–23.

Pickersgill, M. (2005). The taxonomy and ethology of the Afrixalus stuhlmanni complex (Anura: Hyperoliidae). *Steenstrupia*, *29*(1), 1-38.

Pickersgill, M. (2007). *Frog search results of expeditions to southern and eastern africa*. Frankfurt am Main; Lanesboro, Minn.: Serpent’s Tale NHBD / Edition Chimaira.

Pimenta, B. V., & Cruz, C. A. G. (2004). The tadpole and advertisement call of Physalaemus aguirrei Bokermann, 1966 (Amphibia, Anura, Leptodactylidae). *Amphibia-Reptilia*, *25*(2), 197-204.

Pimenta, B. V., Cruz, C. A. G., & Silvano, D. L. (2005). A new species of the genus Physalaemus Fitzinger, 1826 (Anura, Leptodactylidae) from the Atlantic Rain Forest of southern Bahia, Brazil. *Amphibia-Reptilia*, *26*(2), 201-210.

Pimenta, B. V. S., Nunes, I., & Cruz, C. A. G. (2007). Notes on the poorly known phyllomedusine frog *Hylomantis aspera* Peters, 1872 (Anura, hylidae). *South American Journal of Herpetology*, *2*(3), 206–214. [https://doi.org/10.2994/1808-9798(2007)2[206:NOTPKP]2.0.CO;2](about:blank)

Pimenta, B. V., Wachlevski, M., & Cruz, C. A. G. (2008). Morphological and Acoustical Variation, Geographic Distribution, and Conservation Status of the Spinythumb Frog Crossodactylus bokermanni (Anura, Hylodidae). *Journal of Herpetology*, *42*(3), 481-492.

Pimenta, B. V. S., Napoli, M. F., & Haddad, C. F. B. (2009). A new species of casque-headed tree frog, genus *Aparasphenodon* Miranda-Ribeiro (Amphibia: Anura: Hylidae), from the Atlantic Rainforest of southern Bahia, Brazil. *Zootaxa*, *2123*, 46–54.

Pinheiro, P. D. P., Pezzuti, T. L., & Garcia, P. C. de A. (2012). The Tadpole and Vocalizations of *Hypsiboas polytaenius*(Cope, 1870) (Anura, hylidae, hylinae). *South American Journal of Herpetology*, *7*(2), 123–133. https://doi.org/10.2994/057.007.0202

Pinheiro, P. D. P., Taucce, P. P. G., Leite, F. S. F., & Garcia, P. C. D. A. (2014). The advertisement call of the endemic *Bokermannohyla martinsi* (Bokermann, 1964) (Anura: hylidae) from southern Espinhaço range, southeastern Brazil. *Zootaxa*, *3815*(1), 147–150. <https://doi.org/10.11646/zootaxa.3815.1.11>

Pirani, R. M., Mângia, S., Santana, D. J., Assis, B. D., & Feio, R. N. (2010). Rediscovery, distribution extension and natural history notes of Hylodes babax (Anura, Hylodidae) with comments on southeastern Brazil biogeography. *south american Journal of Herpetology*, *5*(2), 83-88.

Pombal, J. P., Sazima, I., & Haddad, C. F. B. (1994). Breeding behavior of the pumpkin toadlet, Brachycephalus ephippium (Brachycephalidae). *Journal of Herpetology*, *28*(4), 516–519. <https://doi.org/10.2307/1564972>

Pombal Jr., J.P.; R.P. Bastos & C.F.B. Haddad. (1995). Vocalizações de algumas espécies do gênero *Scinax* (Anura, Hylidae) do sudeste do Brasil e comentários taxonômicos. Naturalia, 20: 213-225. <https://repositorio.bc.ufg.br/xmlui/handle/ri/12149>

Pombal Jr., J. P., & Bastos, R. P. (1998). Nova espécie de *Hyla* Laurenti, 1768 do centro-oeste brasileiro e a posição taxonômica de *Hyla microcephala werneri* Cochran, 1952 e *H. microcephala meridiana* B. Lutz, 1952 (Anura, Hylidae). *Boletim do Museu Nacional (N.S.) Zoologia*, 1998, 3901–14.

Pombal Jr, J. P., & Haddad, C. F. (1999). Frogs of the genus *Paratelmatobius* (Anura: Leptodactylidae) with descriptions of two new species. *Copeia*, 1014-1026.

Pombal Jr, J. P., Feio, R. N., & Haddad, C. F. (2002). A new species of torrent frog genus *Hylodes* (Anura: Leptodactylidae) from southeastern Brazil. *Herpetologica*, *58*(4), 462-471.

Pombal Jr, J. P., & Bastos, R. P. (2003). Vocalizations of Scinax perpusillus (A. Lutz & B. Lutz) and S. arduous Peixoto (Anura, Hylidae), with taxonomic comments. *Revista Brasileira de Zoologia*, *20*(4), 607-610.

Pombal Jr, J. P., Carvalho Jr, R. R., Canelas, M. A. S., & Bastos, R. P. (2010). A new Scinax of the S. catharinae species group from Central Brazil (Amphibia: Anura: Hylidae). *Zoologia*, *27*(5), 795-802.

Pombal Jr, J. P., Bilate, M., Gambale, P. G., Signorelli, L., & Bastos, R. P. (2011). A new miniature treefrog of the Scinax ruber clade from the Cerrado of central Brazil (Anura: Hylidae). *Herpetologica*, *67*(3), 288-299

Porter, K. R. (1962). Mating calls and noteworthy collections of some mexican amphibians. *Herpetologica*, *18*(3), 165–171.

Porter, K. R. (1966). Mating calls of six mexican and central american toads (Genus Bufo). *Herpetologica*, *22*(1), 60–67.

Poyarkov, N. A., Jr., Orlov, N. L., Moiseeva, A. V., Pawangkhanant, P., Ruangsuwan, T., Vassilieva, A. B., Galoyan, E. A., Nguyen, T. T., & Gogoleva, S. I. (2015). Sorting out Moss Frogs: mtDNA data on taxonomic diversity and phylogenetic relationships of the Indochinese species of the genus Theloderma (Anura, Rhacophoridae). *Russian Journal of Herpetology*, *22*, 241–280.

Poyarkov Jr, N. A., Van Duong, T., Orlov, N. L., Gogoleva, S. S., Vassilieva, A. B., Nguyen, L. T., & Mahony, S. (2017). Molecular, morphological and acoustic assessment of the genus Ophryophryne (Anura, Megophryidae) from Langbian Plateau, southern Vietnam, with description of a new species. *ZooKeys*, (672), 49.

Prado, G., Borgo, J. H., Abrunhosa, P. A., & Wogel, H. (2003). Comportamento reprodutivo, vocalização e redescrição do girino de *Phrynohyas mesophaea* (Hensel, 1867) do Sudeste do Brasil (Amphibia, Anura, Hylidae). *Boletim do Museu Nacional, Nova Série, Zoologia*, *510*, 1-11.

Prado, G. M. and J. P. Pombal-Jr. (2008). Espécies de *Proceratophrys* Miranda-Ribeiro, 1920 com apêndices palpebrais (Anura; Cycloramphidae). *Arquivos de Zoologia*, 39(1): 1–85

Preininger, D., Böckle, M., & Hödl, W. (2007). Comparison between anuran acoustic communities of two habitat types in the Danum Valley Conservation Area, Sabah, Malaysia. *Salamandra*, *43*, 129–138.

Preininger, D., Boeckle, M., Freudmann, A., Starnberger, I., Sztatecsny, M., & Hödl, W. (2013). Multimodal signaling in the small torrent frog (Micrixalus saxicola) in a complex acoustic environment. *Behavioral Ecology and Sociobiology*, *67*(9), 1449-1456.

Price, D. S. (1994). Observations on the ecology and vocalization of Xenorhina oxycephala (Schlegel),(Anura: Microhylidae) of New Guinea. *Science in New Guinea*, *20*(2), 3.

Prigioni, C. M., and J. A. Langone. (2000). Una nueva especie de *Melanophryniscus* Gallardo, 1961, de Argentina y Paraguay (Amphibia, Anura, Bufonidae). *Comunicaciones Zoológicas del Museo de Historia Natural de Montevideo*, *12*, 1–11.

Priti, H., Roshmi, R. S., Ramya, B., Sudhira, H. S., Ravikanth, G., Aravind, N. A., & Gururaja, K. V. (2016). Integrative taxonomic approach for describing a new cryptic species of bush frog (Raorchestes: anura: rhacophoridae) from the western ghats, india. *PLOS ONE*, *11*(3), e0149382. https://doi.org/10.1371/journal.pone.0149382

Provete, D. B., Garey, M. V., Toledo, L. F., Nascimento, J., Lourenço, L. B., Rossa-Feres, D. D. C., & Haddad, C. F. (2012). Redescription of Physalaemus barrioi (Anura: Leiuperidae). *Copeia*, *2012*(3), 507-518.

Pugliese, A., Pombal Jr, J. P., & Sazima, I. (2004). A new species of *Scinax* (Anura: Hylidae) from rocky montane fields of the Serra do Cipó, Southeastern Brazil. *Zootaxa*, *688*(1), 1-15.

Pugliese, A., Baeta, D., & Pombal Jr, J. P. (2009). A new species of Scinax (Anura: Hylidae) from rocky montane fields in southeastern and central Brazil. *Zootaxa*, *2269*, 53-64.

Rao, D. Q., Wilkinson, J. A., & Zhang, M. W. (2006). A new species of the genus Vibrissaphora (Anura: Megophryidae) from Yunnan Province, China. *Herpetologica*, *62*(1), 90-95.

Reynolds, R. P., & Foster, M. S. (1992). Four new species of frogs and one new species of snake from the chapare region of bolivia, with notes on other species. *Herpetological Monographs*, *6*, 83–104. <https://doi.org/10.2307/1466963>

Richards, S. J. (2001). A new species of torrent-dwelling frog (Anura: Hylidae: *Litoria*) from the mountains of Indonesian New Guinea (West Papua). Memoirs of the Queensland Museum 46: 733–740.

Richards, S. J., G. R. Johnston, and T. C. Burton. (1994). A remarkable new *Asterophryine* microhylid frog from the mountains of New Guinea. *Memoirs of the Queensland Museum* 37: 281–286.

Richards, S. J., & Iskandar, D. T. (2001). A new species of tree frog (Anura, Hylidae, Litoria) from the mountains of Irian Jaya, Indonesia. *Alytes*, *18*(3-4), 141-152.

Richards, S.J., Oliver, P., Dahl, C., and Tjaturadi, B. (2006). A new species of large green treefrog (Anura: Hylidae: Litoria) from northern New Guinea. *Zootaxa* 1208: 57-68.

Richards, S. J., and P. M. Oliver. (2006). A new species of torrent-dwelling *Litoria* (Anura: Hylidae) from the Kikori Integrated Conservation and Development Project area, Papua New Guinea. Salamandra 42: 231–238.

Richards, S. J., & Oliver, P. M. (2007). a new species of Hylophorbus (anura, Microhylidae) from the Huon Peninsula, Papua new Guinea. *Zoosystematics and Evolution*, *83*(S1), 83-89.

Richards, S. J., & Oliver, P. M. (2010). A new scansorial species of Cophixalus (Anura: Microhylidae) from the Kikori River basin, Papua New Guinea. *Journal of Herpetology*, *44*(4), 555-562.

Richards, S. J., and D. T. Iskandar. (2000). A new minute *Oreophryne* (Anura: Microhylidae) from the mountains of Irian Jaya, Indonesia. *Raffles Bulletin of Zoology* 48: 257–262.

Richards, S. J., Hoskin, C. J., Cunningham, M. J., McDonald, K., & Donnellan, S. C. (2010) [Taxonomic re-assessment of the Australian and New Guinean green-eyed treefrogs Litoria eucnemis, *L. genimaculata* and *L. serrata* (Anura: Hylidae).](https://researchonline.jcu.edu.au/29001/) *Zootaxa*, 2391. pp. 33-46.

Ríos-López, N., & Thomas, R. (2007). A new palustrine *Eleutherodactylus* (Anura: Leptodactylidae) from Puerto Rico. *Zootaxa*, *1512*, 51–64.

Ríos-López, N., & Villanueva, L. J. (2013). Acoustic characteristics of a native anuran (Amphibia) assemblage in a palustrine herbaceous wetland from Puerto Rico. *Life: The Excitement of Biology*, *1*(2):118–135

Ríos-López, N., Flores-Rodríguez, Y. M., Agosto-Torres, E., Vicéns-López, C., & Hernández-Muñíz, R. M. (2015). Life history observations on the Melodious Coqui, *Eleutherodactylus wightmanae* (Anura: Eleutherodactylidae), from Puerto Rico: double clutches and adult predation by the Yellow-Chinned Anole, *Anolis gundlachi* (Squamata: Dactyloidae). *Life: The Excitement of Biology*, *3*(2), 137–148.

Rivero, J. A. (1969). On the identity and relationships of *Hyla luteocellata* Roux (Amphibia, Salientia). *Herpetologica*, *25*, 126–134.

Roberto, I.J. & Ávila, R.W. (2013) The advertisement call of Phyllodytes gyrinaethes Peixoto, Caramaschi & Freire, 2003 (Anura, Hylidae). *Zootaxa*, 3669 (2), 193–196. <http://dx.doi.org/10.11646/zootaxa.3669.2.13>

Roberto, I. J., Cardozo, D., & Avila, R. W. (2013). A new species of *Pseudopaludicola* (Anura, Leiuperidae) from western Piauí State, northeast Brazil. *Zootaxa*, *3636*(2), 348-360.

Roberts JD. (1981) Terrestrial breeding in the Australian leptodactylid frog *Myobatrachus gouldii* (Gray). *Wildlife Research*, 8:451–462

Roberts, J. D. (1984). Terrestrial Egg Deposition and Direct Development in Arenophyrne rotunda Tyler, a Myobatrachid Frog from Coastal Sand Dunes at Shark Bay, W. A. *Wildlife Research*, *11*(1), 191-200.

Roberts, J. D. (1997). Call evolution in Neobatrachus (Anura: Myobatrachidae): speculations on tetraploid origins. *Copeia*, 791-801.

Roberts, J. D., G. Wardell-Johnson, and W. Barendse. (1990). Extended descriptions of *Geocrinia vitellina* and *Geocrinia alba* (Anura: Myobarachidae) from south-western Australia, with comments on the status of *G. lutea*. *Records of the Western Australian Museum* 14: 427–437.

Roberts, J. D., Mahony, M., Kendrick, P., & Majors, C. M. (1991). A new species of burrowing frog, *Neobatrachus* (Anura: Myobatrachidae), from the eastern wheatbelt of Western Australia. *Records of the Western Australian Museum*, *15*, 23-32.

Roberts, J. D.**,** & C.M. Majors (1993). Range extensions, range definitions and call structures for frogs from Western Australia. *Records of the Western Australian Museum* 16:315-322.

Roberts, J. D., & Wardell-Johnson, G. (1995). Call differences between peripheral isolates of the Geocrinia rosea complex (Anura: Myobatrachidae) in southwestern Australia. *Copeia*, 899-906.

Roberts, J. D., Horwitz, P., Wardell-Johnson, G., Maxson, L. R., & Mahony, M. J. (1997). Taxonomy, relationships and conservation of a new genus and species of myobatrachid frog from the high rainfall region of southwestern Australia. *Copeia*, 373-381.

Rocha, P. C., Thompson, J. R., Leite, F. S. F., & Garcia, P. C. D. A. (2016). The advertisement call of Bokermannohyla flavopicta Leite, Pezzuti & Garcia, 2012 (Anura: hylidae) from the mountains of chapada diamantina, bahia, brazil. *Zootaxa*, *4061*(3), 277–280. https://doi.org/10.11646/zootaxa.4061.3.6

Rodrigues, D. de J., Lopes, F. S., & Uetanabaro, M. (2003). Reproductive pattern of Elachistocleis bicolor (Anura, microhylidae) at serra da bodoquena, mato grosso do sul, brazil. *Iheringia. Série Zoologia*, *93*(4), 365–371. <https://doi.org/10.1590/S0073-47212003000400003>

Rodrigues, D. J., Menin, M., Lima, A. P., & Mokross, K. S. (2008). Tadpole and vocalizations of *Chiasmocleis hudsoni* (Anura, Microhylidae) in Central Amazonia, Brazil. *Zootaxa*, *1680*, 55-58.

Rodríguez, L. O., & Myers, C. W. (1993). A new poison frog from Manu National Park, southeastern Peru (Dendrobatidae, epipedobates). American Museum novitates ; no. 3068. Recuperado de <http://digitallibrary.amnh.org/handle/2246/4965>

Rodríguez, L. O., & Duellman, W. E. (1994). Guide to the frogs of the Iquitos region, Amazonian Peru. *The University of Kansas Natural History Museum Special Publication*, *22*(i–ii), 1–80.

Rodríguez-Tejeda, R. E., Méndez-Cárdenas, M. G., Islas-Villanueva, V., & Garcia, C. M. (2014). Geographic variation in the advertisement calls of *Hyla eximia* and its possible explanations. *PeerJ*, *2*, e420.

Rödel, M.-O. (2000). *Herpetofauna of west africa, vol. I: amphibians of the west african savanna*. Frankfurt am Main: Chimaira.

Rödel, M. O., Grafe, T. U., Rudolf, V. H., & Ernst, R. (2002). A review of West African spotted Kassina, including a description of *Kassina schioetzi* sp. nov. (Amphibia: Anura: Hyperoliidae). *Copeia*, *2002*(3), 800-814.

Rödel, M. O., Gil, M. A. R. L. O. N., Agyei, A. C., Leaché, A. D., Diaz, R. E., Fujita, M. K., & Ernst, R. (2005). The amphibians of the forested parts of south-western Ghana. *Salamandra*, *41*(3), 107-127.

Rödel, M. O., Kosuch, J., Grafe, T. U., Boistel, R., Assemian, N. E., Kouamé, N. G., & Tafforeau, P. (2009). A new tree-frog genus and species from Ivory Coast, West Africa (Amphibia: Anura: Hyperoliidae). *Zootaxa*, *2044*(1), 23-45.

Rojas-Runjaic F.J.M., Infante-Rivero E., Barrio-Amoros C.L. (2016). New records, range extension and call description for the stream-breeding frog *Hyloscirtus lascinius* (Rivero, 1970) in Venezuela. *Amphibian & Reptile Conservation*, *10*, 34–38.

Ron, S. R., Cannatella, D. C., & Coloma, L. A. (2004). Two new species of Physalaemus (Anura: Leptodactylidae) from western Ecuador. *Herpetologica*, *60*(2), 261-275.

Ron, S. R., Toral, E., Rivera, M., & Terán-Valdez, A. (2010). A new species of *Engystomops* (Anura: Leiuperidae) from southwestern Ecuador. *Zootaxa*, *2606*(1), 25-49.

Ron, S., Venegas, P. J., Toral, E., Read, V. M., Ortiz, D., & Manzano, A. (2012). Systematics of the Osteocephalus buckleyi species complex (Anura, hylidae) from Ecuador and Peru. *ZooKeys*, *229*, *229*, 1–52. <https://doi.org/10.3897/zookeys.229.3580>

Rosset, S.D. (2008) New species of Odontophrynus Reinhardt and Lütken 1862 (Anura: Neobatrachia) from Brazil and Uruguay. Journal of Herpetology, 42, 134–144. http://dx.doi.org/10.1670/07-088r1.1

Rosset, S. D., Ferraro, D. P., Alcalde, L., & Basso, N. G. (2007). A revision of Odontophrynus barrioi (Anura: Neobatrachia): morphology, osteology, vocalizations, and geographic distribution. *South American Journal of Herpetology*, *2*(2), 97-106.

Rosset, S., & Baldo, D. (2014). The advertisement call and geographic distribution of Odontophrynus lavillai Cei, 1985 (Anura: Odontophrynidae). *Zootaxa*, *3784*(1), 079-083.

Rosso, A., Castellano, S., & Giacoma, C. (2004). The advertisement call *of Hyla intermedia* and *H. sarda*. *Italian Journal of Zoology*, *71*(sup2), 169–173. <https://doi.org/10.1080/11250000409356629>

Rounsevell, D. E., D. Ziegeler, P. B. Brown, M. M. Davies, and M. J. Littlejohn. (1994). A new genus and species of frog (Anura: Leptodactylidae: Myobatrachinae) from southern Tasmania. *Transactions of the Royal Society of South Australia* 118: 171–185.

Roy, D., & Elepfandt, A. (1993). Bioacoustic analysis of frog calls from northeast India. *Journal of Biosciences*, *18*(3), 381–393. <https://doi.org/10.1007/BF02702996>

Rowley, J. J. L., Nguyen, S. N., Dau, V. Q., Nguyen, T. T., & Cao, T. T. (2011). A new species of *Gracixalus* (Anura: Rhacophoridae) with a hyperextended vocal repertoire from Vietnam. *Zootaxa*, *3125*, 22–38.

Ruas, D. S., Mendes, C. V. D. M., Dias, I. R., & Solé, M. (2012). Description of the advertisement call of *Dendropsophus haddadi* (Bastos and Pombal 1996) (Anura: Hylidae) from southern Bahia, Brazil. *Zootaxa*, *3250*, 63–65.

Ruiz-Carranza, P. M., and J. D. Lynch. (1991). Ranas Centrolenidae de Colombia III. Nuevas especies de *Cochranella* del grupo *granulosa*. Lozania 59: 1–18.

Ryan, M. J., & Drewes, R. C. (1990). Vocal morphology of the Physalaemus pustulosus species group (Leptodactylidae): morphological response to sexual selection for complex calls. *Biological Journal of the Linnean Society*, *40*(1), 37–52. <https://doi.org/10.1111/j.1095-8312.1990.tb00533.x>

Sabino-Pinto, J., Mayerl, C. J., Meilink, W. R., Grasso, D., Raaijmakers, C. C., Russo, V. G., & Glaw, F. (2014). Descriptions of the advertisement calls of three sympatric frog species in the subgenus Vatomantis (genus Gephyromantis) from Madagascar. *Herpetology Notes*, *7*, 67-73.

Salas, N. E., Zavattieri, M. V., Tada, I. E. di, Martino, A. L., & Bridarolli, M. E. (1998). Bioacustical and etho-ecological features in amphibian communities of Southern Cordoba province (Argentina). *Cuadernos de Herpetología*, *12. no. 1*. Recuperado de <http://hdl.handle.net/10915/6300>

Salas, N. E.; E. di Tada. (1988). Análisis bioacústico del canto nupcial de poblaciones de *Odontophrynus achalensis* y *O. occidentalis* (Anura: Leptodactylidae) en la provincia de Córdoba. *Boletín de la Asociación Herpetológica Argentina* 4(2-3): 1.

Salgado-Maldonado, A. L. (2012). *Cuidado parental y selección sexual en Centrolene peristictum (Anura: centrolenidae)*(Licenciatura en Ciencias Biológicas). Pontificia Universidad Católica del Ecuador, Quito. Recuperado de http://repositorio.puce.edu.ec:80/xmlui/handle/22000/4933

Samarasinghe, D. J. S. (2011). Description of the complex advertisement call of Pseudophilautus popularis (Manamendra-arachchi & pethiyagoda, 2005) (Amphibia: rhacophoridae). *Zootaxa*, *3002*(1), 62–64.

Samarasinghe, D. (2012). The advertisement call of Kandyan shrub frog (pseudophilautus rus). *TAPROBANICA: The Journal of Asian Biodiversity*, *4*(1). https://doi.org/10.4038/tapro.v4i1.4388

Sandberger, L., Hillers, A., Doumbia, J., Loua, N., Brede, C., and M. Rödel (2010). 'Rediscovery of the Liberian Nimba toad, *Nimbaphrynoides liberiensis* (Xavier, 1978) (Amphibia: Anura: Bufonidae), and reassessment of its taxonomic status. *Zootaxa*, 2355, 56–68.

Santana, D.J., Sant'Anna, A.C., São-Pedro, V. A., & Feio, R. N. (2009). The adverTisemenT call of ChiasmoCleis bassleri (anura, microhylidae) from souThern amazon, maTo Grosso, Brazil. *South american Journal of herpetology*, *4*(3), 225-228.

Santana, D. J., São-Pedro, V. D. A., Bernarde, P. S., & Feio, R. N. (2010). Descrição do canto de anúncio e dimorfismo sexual em *Proceratophrys concavitympanum* Giaretta, Bernarde & Kokubum, 2000. *Papéis Avulsos de Zoologia (São Paulo)*, *50*(11), 167-174.

Santana, D. J., Mesquita, D. O., & Garda, A. A. (2011a). Advertisement call of *Dendropsophus oliveirai* (Anura, hylidae). *Zootaxa*, *2997*(1), 67–68.

Santana, D. J., Rodrigues, R., Albuquerque, R. L., Laranjeiras, D. O., Protázio, A. S., França, F. G. R., & Mesquita, D. O. (2011b). The advertisement call of Proceratophrys renalis (Miranda-Ribeiro, 1920)(Amphibia: Anura: Cycloramphidae). *Zootaxa*, *2809*, 67-68.

Santana, D. J., Motta, A. P., Pirani, R. M., da Silva, E. T., & Feio, R. N. (2012). Advertisement call and tadpole of chiasmocleis mantiqueira (anura, microhylidae). *Journal of Herpetology*, *46*(1), 14-18.

Santana, D. J., Queiroz, S.S., Wanderley, P. S., São-Pedro, V. de A. , Leite, F. S. F., & Garda, A. A. (2013). Calls and tadpoles of the species of *Lysapsus* (Anura, Hylidae, Pseudae). *Amphibia-Reptilia*, *34*(2), 201-215.

Santos, J.C., Baquero, M., Barrio-Amorós, C.L., Coloma, L.A., Erdtmann, L.K., Lima, A.P., Cannatella, D.C. 2014. Aposematism increases acoustic diversification and speciation in poison frogs. *Proceedings of the Royal Society B-Biological Sciences*, *281*, 9. DOI: 10.1098/rspb.2014.1761

São-Pedro, V. A., Medeiros, P. H., & Garda, A. A. (2011). The advertisement call of *Rhinella granulosa* (Anura, bufonidae). *Zootaxa*, *3092*(1), 60–62.

Savage, J. M. (2002). *The amphibians and reptiles of costa rica: a herpetofauna between two continents, between two seas*. Chicago: University of Chicago Press.

Sazima, I., & Bokermann, W. C. A. (1977). Anfíbios da Serra do Cipó, Minas Gerais, Brasil. 3: Observações sobre a biologia de *Hyla alvarengai* Bok. (Anura, Hylidae). *Revista Brasileira de Biologia*, *37(2)*, 413–417.

Sazima, I., & Bokermann, W. C. A. (1978). Five new species of Leptodactylus from central and southeastern Brazil (Amphibia, Anura, Leptodactylidae). *Revista Brasileira de Biologia*, *38*, 899-912.

Schick, S., Zimkus, B. M., Channing, A., Köhler, J., & Lötters, S. (2010). Systematics of ‘Little Brown Frogs’ from East Africa: recognition of Phrynobatrachus scheffleri and description of a new species from the Kakamega Forest, Kenya (Anura: Phrynobatrachidae). *Salamandra*, *46*(1), 24–36.

Schiotz, A. (1999). *Treefrogs of africa* (1 edition). Frankfurt am Main: Hollywood Import & Export Inc.

Schleich, H. H., & Kästle, W. (2002). Amphibians and Reptiles of Nepal: Biology, Systematics, Field Guide.

Señaris, J.C. & Ayarzagüena, J. (2002). A new species of *Hyla* (Anura; Hylidae) from the highlands of Venezuelan Guayana. *Journal of Herpetology,* *36*, 634–640.

Señaris, J. C., & Ayarzagüena, J. (2005). Revisión taxonómica de la Familia Centrolenidae (Amphibia; Anura) de Venezuela. Sevilla: Publicaciones del Comité Español del Programa Hombre y Biosfera – Red IberoMaB de la UNESCO. No. 7.

Serra-Cobo, J. (1993). Descripción de una nueva especie europea de rana parda (Amphibia, Anura, Ranidae). *Alytes. Paris*, *11*, 1–15.

Seshadri, K. S., Gururaja, K. V., & Aravind, N. A. (2012). A new species of Raorchestes (Amphibia: Anura: Rhacophoridae) from mid-elevation evergreen forests of the southern Western Ghats, India. *Zootaxa*, *3410*, 19–34.

Silva, R.A., Alves Martins, I., & Cerqueira Rossa-Feres, D. D. (2008). Bioacústica e sítio de vocalização em taxocenoses de anuros de área aberta no noroeste paulista. *Biota Neotropica*, *8*(3) : http://www. biotaneotropica.org.br/v8n3/en/abstract?article+bn01608032008.

Silva-Filho, I. S. N., & Juncá, F. A. (2006). Evidence of full species status of the neotropical leaf-frog Phyllomedusa burmeisteri bahiana (A. Lutz, 1925) (Amphibia, Anura, Hylidae). *Zootaxa*, *1113*(1), 51-64.

Silverstone, P. A. (1976). A revision of the poison-arrow frogs of the genus *Phyllobates* Bibron *in* Sagra (family Dendrobatidae). *Science Bulletin. Natural History Museum of Los Angeles County*, 27, 1–53.

Simões, P. I. (2010). Diversificação do complexo *Allobates femoralis* (Anura, Dendrobatidae) em florestas da Amazônia brasileira: desvendando padrões atuais e históricos (Doctoral dissertation, PhD thesis, Instituto Nacional de Pesquisas da Amazônia).

Simões, P. I., & Lima, A. P. (2011). The complex advertisement calls of *Allobates myersi* (Pyburn, 1981) (Anura: Aromobatidae) from São Gabriel da Cachoeira, Brazil. *Zootaxa*, *2988*, 66–68.

Simon, J.E. & Gasparini, J.L. (2003) Descrição da vocalização de Phyllodytes kautskyi Peixoto and Cruz, 1988 (Amphibia, Anura, Hylidae). Boletim do Museu de Biologia Mello Leitão (N. Ser.), 16, 47–54.

Sinsch U, & Juraske N. (2006a). The advertisement calls of hemiphractine marsupial frogs: II. *Gastrotheca plumbea* group. In M. Vences, J. Kohler, T. Ziegler, and W. Böhme (Eds.), *Herpetologia Bonnensis II*, pp 149-52. *Proceedings of the13th Congress of the Societas Europea Herpetologica*, Bonn, Germany.

Sinsch, U., & Juraske, N. (2006b). Advertisement calls of hemiphractine marsupial frogs: I. *Gastrotheca* *marsupiata* group. In M. Vences, J. Köhler, T. Ziegler, and W. Böhme (eds.), *Herpetologia Bonnensis II*, pp. 145–148. *Proceedings of the 13th Congress of the Societas Europaea Herpetologica*, Bonn, Germany.

Sinsch, U., Lümkemann, K., Rosar, K., & C. S., & Dehling, J. M. (2012). Acoustic niche partitioning in an anuran community inhabiting an afromontane wetland (Butare, Rwanda). *African Zoology*, *47*(1), 60–73. <https://doi.org/10.3377/004.047.0122>

Smith, M. J. & Roberts, J. D. (2003). Call repertoire of an Australian treefrog, *Litoria adelaidensis* (Anura, Hylidae). Journal of the Royal Society of Western Australia 86: 91-95.

Solé, M., Dias, I. R., Rodrigues, E. A., Marciano-Jr, E., Branco, S. M., Cavalcante, K. P., & Rödder, D. (2009). Diet of *Leptodactylus ocellatus* (Anura: Leptodactylidae) from a cacao plantation in southern Bahia, Brazil. *Herpetology Notes*, *2*(2009), 9-15.

Solı́s, R., & Penna, M. (1997). Testosterone levels and evoked vocal responses in a natural population of the frogbatrachyla taeniata. *Hormones and Behavior*, *31*(2), 101–109. https://doi.org/10.1006/hbeh.1997.1366

Stejneger, L. (1926). Two new tailless amphibians from western China. *Proceedings of the Biological Society of Washington* 39: 53–54. https://www.biodiversitylibrary.org/part/43578#/summary

Stöck, M., Sicilia, A., Belfiore, N. M., Buckley, D., Lo Brutto, S., Lo Valvo, M., & Arculeo, M. (2008). Post-Messinian evolutionary relationships across the Sicilian channel: Mitochondrial and nuclear markers link a new green toad from Sicily to African relatives. *BMC Evolutionary Biology*, *8*, 56. <https://doi.org/10.1186/1471-2148-8-56>

Stuart, L. C. (1954). Descriptions of some new amphibians and reptiles from Guatemala. *Proceedings of the Biological Society of Washington*, *67*, 159–178.

Stuart, B. L., Rowley, J. J., Tran, D. T. A., Le, D. T. T., & Hoang, H. D. (2011). The Leptobrachium (Anura: Megophryidae) of the Langbian Plateau, southern Vietnam, with description of a new species. *Zootaxa*, *2804*(1), 25-40.

Stuart, B. L., Phimmachak, S., Seateun, S., & Sheridan, J. A. (2013). A new *Philautus* (Anura: rhacophoridae) from northern Laos allied to *P*. *abditus* Inger, Orlov & Darevsky, 1999. *Zootaxa*, *3745*(1), 73–83. <https://doi.org/10.11646/zootaxa.3745.1.6>

Stewart, D. (1998). *Australian frog calls: subtropical east*. Nature Sound. http://www.naturesound.com.au/cd_frogsTNE.htm

Suazo-Ortuño, I., Alvarado-Díaz, J., Raya-Lemus, E., & Martinez-Ramos, M. (2007). Diet of the mexican marbled toad (*Bufo marmoreus*) in conserved and disturbed tropical dry forest. *The Southwestern Naturalist*, *52*(2), 305–309. https://doi.org/10.1894/0038-4909(2007)52[305:DOTMMT]2.0.CO;2

Sullivan, B. K., Malmos, K. B., Gergus, E. W. A., & Bowker, R. W. (2000). Evolutionary implications of advertisement call variation in *Bufo debilis*, *B. punctatus*, and *B. retiformis*. *Journal of Herpetology*, *34*(3), 368–374. https://doi.org/10.2307/1565358

Tandy, M., J. P. Bogart, M. J. Largen & D. J. Feener (2013) Variation and Evolution In *Bufo kerinyagae* Keith, *B. regularis* Reuss and *B. asmarae* Tandy et al. (Anura Bufonidae), Monitore Zoologico Italiano. Supplemento, 20:1, 211-267, DOI: [10.1080/03749444.1985.10736699](https://doi.org/10.1080/03749444.1985.10736699)

Tárano, Z. (2010). Advertisement calls and calling habits of frogs from a flooded savanna of venezuela. *South American Journal of Herpetology*, *5*(3), 221–240. <https://doi.org/10.2994/057.005.0308>

Tarrant, J. (2012). *Conservation assessment of threatened frogs in KwaZulu–Natal and a national assessment of chytrid infection in threatened South African species* (Doctoral dissertation, North-West University). Available at: http://dspace.nwu.ac.za/bitstream/handle/10394/8490/Tarrant_J.pdf?sequence=1&isAllowed=y

Taucce, P. P. G., Leite, F. S. F., Santos, P. S., Feio, R. N., & Garcia, P. C. A. (2012). The advertisement call, color patterns and distribution of *Ischnocnema izecksohni* (Caramaschi and Kisteumacher, 1989) (Anura, brachycephalidae). *Papéis Avulsos de Zoologia*, *52*(9), 112–120. <https://doi.org/10.1590/S0031-10492012000900001>

Taucce, P. P. G., Pinheiro, P. D. P., Leite, F. S. F., & Garcia, P. C. A. (2015). Advertisement call and morphological variation of the poorly known and endemic Bokermannohyla juiju Faivovich, Lugli, Lourenço and Haddad, 2009 (Anura: hylidae) from Central Bahia, Brazil. *Zootaxa*, *3915*(1), 99–110. https://doi.org/10.11646/zootaxa.3915.1.4

Taylor, E. H., & Taylor, E. H. (1962). The amphibian fauna of thailand. *The University of Kansas Science Bulletin*, *43*, 265–599. https://doi.org/10.5962/bhl.part.13347

Teixeira, M., Vechio, F. D., Recoder, R. S., Carnaval, A. C., Strangas, M., Damasceno, R. P., Rodrigues, M. T. (2012). Two new species of marsupial tree-frogs genus *Gastrotheca* Fitzinger, 1843 (Anura, hemiphractidae) from the Brazilian Atlantic Forest. *Zootaxa*, *3437*(1), 1–23.

Teixeira, B. F. D. V., Giaretta, A. A., & Pansonato, A. (2013). The advertisement call of Dendropsophus tritaeniatus (Bokermann, 1965) (Anura: hylidae). *Zootaxa*, *3669*(2), 189–192. https://doi.org/10.11646/Zootaxa.3669.2.12

Teixeira, B. F. V. & Giaretta, A. A. (2015). Setting a fundament for taxonomy: advertisement calls from the type localities of three species of the *Dendropsophus rubicundulus* group (Anura: Hylidae). Salamandra, *51(2)*, 137–146.

Thomé, M.T., Oyamaguchi, H.M. & Brasileiro, C.A. (2007) Amphibia, Anura, Leiuperidae, *Physalaemus bokermanni*: Distribution extension. *Check List* 3(1), 1–3

Thomé, M. T. C., & Brasileiro, C. A. (2007). Sexual Dimorphism, habitat use and seasonal abundance of Elachistocleis cf. ovalis (Anura: Microhylidae) in a Cerrado remnant of São Paulo state, southeastern Brazil. *Biota Neotropica*, *7*(1). http://www.biotaneotropica.org.br/v7n1/pt/abstract?article+bn00307012007
ISSN 1676-0603.

Thomson, S. A., Littlejohn, M. J., Robinson, W. A., & Osborne, W. S. (1996). Taxonomy of the *Litoria aurea* complex: a re-evaluation of the Southern Tableland populations of the Australian Capital Territory and New South Wales. *Australian Zoologist*, *30*, 158–169.

Tobias, M., Evans, B. J., & Kelley, D. B. (2011). Evolution of advertisement calls in African clawed frogs. *Behaviour*, *148*(4), 519–549.

Toledo, L. F., & Haddad, C. F. (2005). Acoustic repertoire and calling behavior of *Scinax fuscomarginatus* (Anura, Hylidae). *Journal of Herpetology*, *39*(3), 455–464.

Toledo, L. F., Garcia, P. C., Lingnau, R., & Haddad, C. F. (2007a). A new species of *Sphaenorhynchus* (Anura; Hylidae) from Brazil. *Zootaxa*, *1658*(1), 57–68.

Toledo, L. F., Giovanelli, J. G. R., Giasson, L. O. M., Prado, C. P. A., Guimarães, L. D., Bastos, R. P., & Haddad, C. F. B. (2007b). *Guia interativo dos anfíbios anuros do Cerrado, Campos Rupestres e Pantanal*. Editora *Neotrópica, São Paulo*, Brasil. DVD-ROM. [http://www.editoraneotropica.com.br/catalogo.html#](http://www.editoraneotropica.com.br/catalogo.html)

Toledo, L. F., Siqueira, S., Duarte, T. C., Veiga-Menoncello, A. C. P., Recco-Pimentel, S. M., & Haddad, C. F. (2010). Description of a new species of *Pseudopaludicola* Miranda-Ribeiro, 1926 from the state of São Paulo, southeastern Brazil (Anura, Leiuperidae). *Zootaxa*, *2496*(1), 38–48.

Toro-Sánchez, T., & Bernal-Bautista, M. H. (2015). The advertisement call of *Diasporus gularis* and *D. tinker* from the pacific region of colombia. *South American Journal of Herpetology*, *10*(2), 116–120. <https://doi.org/10.2994/SAJH-D-14-00041.1>

Trueb, L., & Cannatella, D. C. (1986). Systematics, morphology, and phylogeny of genus *Pipa* (Anura: Pipidae). *Herpetologica*, 412–449.

Tsiora, A., & Kyriakopoulou-Sklavounou, P. (2002). Female reproductive cycle of the water frog *Rana epeirotica* in northwestern Greece. *Amphibia-Reptilia*, *23*(3), 269–280. https://doi.org/10.1163/15685380260449153

Tsuji-Nishikido, B. M., Kaefer, I. L., de Freitas, F. C., Menin, M., & Lima, A. P. (2012). Significant but not diagnostic: Differentiation through morphology and calls in the Amazonian frogs Allobates nidicola and A. masniger. *Herpetological Journal*, *22(2)*, 105–114.

Turner, A. A., de Villiers, A. L., Dawood, A., & Channing, A. (2004). A new species of *Arthroleptella* Hewitt, 1926 (Anura: Ranidae) from the Groot Winterhoek mountains of the Western Cape province, South Africa. *African Journal of Herpetology*, *53*(1), 1–12. https://doi.org/10.1080/21564574.2004.9635493

Turner, A. A., & Channing, A. (2008). A new species of *Arthroleptella* Hewitt, 1926 (Anura: Pyxicephalidae) from the Klein Swartberg mountain, Caledon, South Africa. *African Journal of Herpetology*, *57*(1), 1–12. https://doi.org/10.1080/21564574.2008.9635564

Tyler, M. J. (1998). *Australian frogs: a natural history*. Cornell University Press.

Tyler, M.J. and Martin, A.A. (1975). ''Australian leptodactylid frogs of the *Cyclorana australis* complex.'' *Transactions of the Royal Society of South Australia*, *99(2)*, 93-99.

Tyler, M.J. & Martin, A.A. (1977) Taxonomic studies of some Australian leptodactylid frogs of the genus *Cyclorana* Steindachner. *Records of the South Australian Museum*, *17*, 261–276.

Tyler, M. J., Martin, A. A., & Davies, M. (1979). Biology and Systematics of a New Limnodynastine Genus (Anura: Leptodactylidae) From North-Western Australia. *Australian Journal of Zoology*, *27*(1), 135-150.

Tyler, M. J., Davies, M., & Martin, A. A. (1981). New and rediscovered species of frogs from the Derby-Broome area of Western Australia. *Records of the Western Australian Museum*, *9*(2), 147-172.

Tyler, M. J., M. M. Davies, and A. A. Martin. (1981). Australian frogs of the leptodactylid genus *Uperoleia* Gray. *Australian Journal of Zoology, Supplemental Series* 29 (79): 1–64.

Tyler, M. J., Davies, M., & Watson, G. F. (1986). The frog fauna of groote eylandt, northern territory, australia. *Zoological Journal of the Linnean Society*, *88*(1), 91–101. https://doi.org/10.1111/j.1096-3642.1986.tb00878.x

Tyler, M. J., and P. Doughty. (2009). *Field Guide to Frogs of Western Australia*. Fourth Edition. Perth: Western Australian Museum.

Tyler, M. J., & Knight, F. (2011). *Field guide to the frogs of australia: revised edition*. Csiro Publishing.

Twomey, E., & Brown, J. L. (2008). A partial revision of the *Ameerega hahneli* complex (Anura: dendrobatidae) and a new cryptic species from the East-Andean versant of Central Peru. *Zootaxa*, *1757*(1), 49–65.

Ueda, H. (1994). Mating calls of the pond frog species distributed in the Far East and their artificial hybrids. *Scientific report of the Laboratory for Amphibian Biology, Hiroshima University*, *13*, 197–232.

Vaira, M. (2001). Breeding biology of the leaf frog, *Phyllomedusa boliviana* (Anura, Hylidae). *Amphibia-Reptilia*, *22*(4), 421-429.

Vaira, M., & Ferrari, L. (2008). A new species of *Oreobates* (Anura: strabomantidae) from the Andes of northern Argentina. *Zootaxa*, *1908*(1), 41–50.

Vaira, M., Ferrari, L., & Akmentins, M. S. (2011). Vocal repertoire of an endangered marsupial frog of Argentina, *Gastrotheca christiani* (Anura: Hemiphractidae). *Herpetology Notes*, *4*, 279–284.

Valetti, J. A., Salas, N. E., & Martino, A. L. (2013). Bioacoustic of the advertisement call of *Ceratophrys cranwelli* (Anura: ceratophryidae). *Revista de Biología Tropical*, *61*(1), 273–280.

Vallan, D., Vences, M., & Glaw, F. (2003). Two new species of the *Boophis mandraka* complex (Anura, Mantellidae) from the Andasibe region in eastern Madagascar. *Amphibia-Reptilia*, *24*(3), 305-319.

Vallan, D., Glaw, F., & Vences, M. (2005). The calls of *Plethodontohyla inguinalis* from eastern Madagascar. *Spixiana*, 28 191-93.

Van Kampen, P. N. (1923). The Amphibia of the Indo-Australian Archipelago. Leiden: E. J. Brill Ltd.

Vargas-Salinas, F., & Amézquita, A. (2014). Abiotic noise, call frequency and stream-breeding anuran assemblages. *Evolutionary Ecology*, *28*(2), 341–359. <https://doi.org/10.1007/s10682-013-9675-6>

Vaz-Silva W., Di-Bernardo M., Guimarães L.D., Bastos R.P. (2007). Territoriality, agonistic behaviour, and vocalization in *Pseudis bolbodactylus* A. Lutz, 1925 (Anura: Hylidae) from Central Brazil. *Salamandra* 43: 35–42.

Vaz-Silva, W., & Maciel, N. M. (2011). A new cryptic species of *Ameerega* (Anura: dendrobatidae) from Brazilian Cerrado. *Zootaxa*, *2826*(1), 57–68.

Vejarano, S., Thomas, M., Glaw, F., & Vences, M. (2006). Advertisement call and tadpole morphology of the clutch-guarding frog Mantidactylus argenteus from eastern Madagascar. *African Zoology*, *41*(2), 164-169.

Veloso, A., Celis-Diez, J. L., Guerrero, P. C., Méndez, M. A., Iturra, P., & Simonetti, J. A. (2005). Description of a new *Eupsophus species* (Amphibia, leptodactylidae) from the remnants of maulino forest, central chile. *Herpetological Journal*, *15*(3), 159–165.

Vences, M., Glaw, F., Jesu, R., & Schimmenti, G. (2000). A new species of Heterixalus (Amphibia: Hyperoliidae) from western Madagascar. *African Zoology*, *35*(2), 269-276.

Vences, M., & Glaw, F. (2002). Two new treefrogs of the Boophis rappiodes group from eastern Madagascar (Amphibia Mantellidae). *Tropical Zoology*, *15*(1), 141-163.

Vences, M., Andreone, F., Glaw, F., Raminosoa, N., Randrianirina, J. E., & Vieites, D. R. (2002). Amphibians and reptiles of the Ankaratra Massif: reproductive diversity, biogeography and conservation of a montane fauna in Madagascar. *Italian Journal of Zoology*, *69*(3), 263-284.

Vences, M., C. J. Raxworthy, R. A. Nussbaum & F. Glaw (2003). A revision of the *Scaphiophryne* *marmorata* complex of marbled toads from Madagascar, including the description of a new species. *Herpetological Journal* **13**: 69-79.

Vences, M., & Glaw, F. (2004). Revision of the subgenus Chonomantis (Anura: Mantellidae: Mantidactylus) from Madagascar, with description of two new species. *Journal of Natural History*, *38*(1), 77-118.

Vences, M., Andreone, F., Glaw, F., & Randrianirina, J. E. (2003). Molecular and bioacoustic divergence in Mantidactylus granulatus and M. zavona n. sp.(Anura: Mantellidae): bearings for the biogeography of northern Madagascar. *African Zoology*, *38*(1), 68-78.

Vences, M., F. Andreone, and D. R. Vieites. (2005a). New treefrog of the genus *Boophis* Tschudi 1838 from the northwestern rainforests of Madagascar. *Tropical Zoology* 18: 237–249.

Vences, M., Andreone, F., & Glaw, F. (2005b). A new microhylid frog of the genus Cophyla from a transitional forest in northwestern Madagascar. *African Zoology*, *40*(1), 143-149.

Vences, M., Glaw, F., & Marquez, R. (2006). The calls of the frogs of Madagascar. *Fonoteca Zoológica and Alosa, Barcelona*.

Vences, M., Pabijan, M., Kohler, J., & Glaw, F. (2010a). Two syntopic and microendemic new frogs of the genus Blommersia from the east coast of Madagascar. *African Journal of Herpetology*, *59*(2), 133-156.

Vences, M., Glaw, F., Köhler, J., & Wollenberg, K. C. (2010). Molecular phylogeny, morphology and bioacoustics reveal five additional species of arboreal microhylid frogs of the genus Anodonthyla from Madagascar. *Contributions to Zoology*, *79*(1).

Venegas, P. J., & Ron, S. R. (2014). First records of *Rhinella poeppigii* (Tschudi 1845) from Ecuador, with a distribution map (Anura: bufonidae). *Herpetology Notes*, *7*(0), 713–716.

Verdade, V. K., Rodrigues, M. T., Cassimiro, J., Pavan, D., Liou, N., & Lange, M. C. (2008). Advertisement call, vocal activity, and geographic distribution of Brachycephalus hermogenesi (Giaretta and Sawaya, 1998)(Anura, brachycephalidae). *Journal of Herpetology*, *42*(3), 542–549. <https://doi.org/10.1670/07-287.1>

Vertucci, S., Pepper, M., Edwards, D. L., Roberts, J. D., Mitchell, N., & Keogh, J. S. (2017). Evolutionary and natural history of the turtle frog, Myobatrachus gouldii, a bizarre myobatrachid frog in the southwestern Australian biodiversity hotspot. *PloS one*, *12*(3), e0173348.

Vigny, C. (2009). The mating calls of 12 species and sub-species of the genus *Xenopus* (Amphibia : Anura). *Journal of Zoology*, *188*(1), 103–122. https://doi.org/10.1111/j.1469-7998.1979.tb03394.x

Vilaça, T. R. A., Silva, J. R. D. S., & Solé, M. (2011). Vocalization and territorial behaviour of *Phyllomedusa nordestina* Caramaschi, 2006 (Anura: Hylidae) from southern Bahia, Brazil. *Journal of Natural History*, *45*(29-30), 1823-1834.

Xiong, R., Matsui, M., Nishikawa, K., & Jiang, J. (2015). Advertisement Calls of Two Horned Frogs, *Megophrys kuatunensis*and *M. huangshanensis*, from China (Anura, megophryidae). *Current Herpetology*, *34*(1), 51–59. <https://doi.org/10.5358/hsj.34.51>

Young, J. E., Tyler, M. J., & Kent, S. A. (2005). Diminutive new species of Uperoleia Grey (Anura: Myobatrachidae) from the vicinity of Darwin, Northern Territory, Australia. *Journal of Herpetology*, *39*(4), 603-609.

Yu, B., & Zheng, R.-q. (2009). The advertisement call of the giant spiny frog *Paa spinosa*. *Current Zoology*, *55*, 411–415.

Wang, J., Cui, J., Shi, H., Brauth, S. E., & Tang, Y. (2012). Effects of body size and environmental factors on the acoustic structure and temporal rhythm of calls in rhacophorus dennysi: effects of body size and environmental factors on the acoustic structure and temporal rhythm of calls in *Rhacophorus dennysi*. *Asian Herpetological Research*, 3(3), 205–212. https://doi.org/10.3724/SP.J.1245.2012.00205

Watson, G. F., & Gerhardt, H. C. (1997). The breeding biology and advertisement call of Litoria splendida Tyler, Davies and Martin. *Trans. R. Soc. S. Aust*, *121*, 119–124.

Wasonga, D. V., & Channing, A. (2013). Identification of sand frogs (Anura: Pyxicephalidae: *Tomopterna*) from Kenya with the description of two new species. *Zootaxa*, *3734*(2), 221–240. https://doi.org/10.11646/zootaxa.3734.2.7

Weber, L. N., Gonzaga, L. P., & Carvalho-e-Silva, S. P. (2005). A new species of Physalaemus Fitzinger, 1826 from the lowland Atlantic forest of Rio de Janeiro state, Brazil (Amphibia, Anura, Leptodactylidae). *Arquivos do Museu Nacional*, *63*(4), 677–684.

Wheeler, C. A., & Welsh, H. H. W. (2008). Mating strategy and breeding patterns of the foothill yellow-legged frog Rana boylii. *Herpetological Conservation and Biology*, *3(2)*, 128–142.

Wen, A., Vasquez, N., & Castroviejo-Fisher, S. (2012). Description of the previously unknown advertisement calls of *Hyalinobatrachium fragile*, *H. pellucidum* , and *Vitreorana antisthenesi* (Amphibia: centrolenidae). *Zootaxa*, *3480*(1), 80–87.

Werner, F. (1901). Beschreibung neuer Frösche aus Bolivia, Ostindien und Neu-Guinea. *Zoologischer Anzeiger*, *24*, 97–103.

Weygoldt, P. (1976). Beobachtungen zur Biologie und Ethologie von Pipa (Hemipipa) carvalhoi Mir. Rib. 1937.(Anura, Pipidae). *Ethology*, *40*(1), 80-99.

Weygoldt, P., & Peixoto, O. L. (1987). *Hyla ruschii*n. Sp. , a new frog from the atlantic forest domain in the state of espirito santo, Brazil (Amphibia, hylidae). *Studies on Neotropical Fauna and Environment*, *22*(4), 237–247. <https://doi.org/10.1080/01650528709360736>

Wickramasinghe, L. J. M., Munindradasa, D. A. I., & Fernando, P. (2012). A new species of Polypedates Tschudi (Amphibia, Anura, Rhacophoridae) from Sri Lanka. *Zootaxa*, *3498*, 63–80.

Wijayathilaka, N., & Meegaskumbura, M. (2016). An acoustic analysis of the genus Microhyla (Anura: Microhylidae) of Sri Lanka. *PloS one*, *11*(7), e0159003.

Wildenhues, M. J., Bagaturov, M. F., Schmitz, A., T. A. , Tran, D. A. T., Hendrix, R., & Ziegler, T. (2011). Captive management and reproductive biology of Orlov's Treefrog, Rhacophorus orlovi Ziegler & Köhler, 2001 (Amphibia: Anura: Rhacophoridae), including larval description, colour pattern variation and advertisement call. *Der Zoologische Garten*, 80. 10.1016/j.zoolgart.2011.10.002.

Willaert, B., Suyesh, R., Garg, S., Giri, V. B., Bee, M. A., & Biju, S. D. (2016). A unique mating strategy without physical contact during fertilization in Bombay Night Frogs (Nyctibatrachus humayuni) with the description of a new form of amplexus and female call. *PeerJ*, *4*, e2117.

Weygoldt, P. and O. L. Peixoto. (1985). A new species of horned toad (*Proceratophrys*) from Espírito Santo, Brazil (Amphibia: Salientia: Leptodactylidae). *Senckenbergiana Biologica*, 66(1/3): 1–8.

Wogel, H., Abrunhosa, P. A., & Pombal Jr, J. P. (2002). Atividade reprodutiva de Physalaemus signifer (Anura, Leptodactylidae) em ambiente temporário. *Iheringia, Série Zoologia*, *92*(1), 57-70.

Wogel, H., Abrunhosa, P. A., & Pombal Jr, J. P. (2004a). Vocalizations and aggressive behavior of *Phyllomedusa rohdei* (Anura: Hylidae). *Herpetological Review*, *35*(3), 239-243.

Wogel, H., Abrunhosa, P. A., & Prado, G. M. (2004b). The tadpole of *Chiasmocleis carvalhoi* and the advertisement calls of three species of Chiasmocleis (Anura, Microhylidae) from the Atlantic rainforest of southeastern Brazil. *Phyllomedusa*, *3*(2), 133-140.

Wollenberg, K. C., Andreone, F., Glaw, F., & Vences, M. (2008). Pretty in pink: a new treefrog species of the genus Boophis from north-eastern Madagascar. *Zootaxa*, *1684*, 58-68.

Wood, P. L., Grismer, L. L., Ahmad, N., & Senawi, J. (2008). Two new species of torrent-dwelling toads *Ansonia stoliczka*, 1870 (Anura: bufonidae) from peninsular malaysia. *Herpetologica*, *64*(3), 321–340. <https://doi.org/10.1655/07-065.1>

Zainudin, R., Rahman, M. A., Zain, B. M. M., Shukor, M. N., Inger, R. F., & Norhayati, A. (2010). Mating calls description of five species of frogs from the genus *Hylarana* Tschudi 1838 (Amphibia, Anura, Ranidae) from Sarawak, Malaysia. *Sains Malaysiana*, *39*, 363–369.

Zank, C., Di-Bernardo, M., Lingnau, R., Colombo, P., Fusinatto, L. A., & da Fonte, L. F. (2008). Calling activity and agonistic behavior of *Pseudis minuta* Günther, 1858 (Anura, Hylidae, Hylinae) in the Reserva Biológica do Lami, Porto Alegre, Brazil. *South American Journal of Herpetology*, *3*(1), 51-57.

Zheng, Y., Rao, D., Murphy, R. W., & Zeng, X. (2011). Reproductive behaviour and underwater calls in the Emei Moustache Toad, Leptobrachium boringii. *Asian Herp Res*, *2*, 199-215.

Ziegler, T., & Köhler, J. (2001). *Rhacophorus orlovi* sp. n., ein neuer Ruderfrosch aus Vietnam (Amphibia: Anura: Rhacophoridae). Sauria. Berlin 23: 37–46.

Zina, J., & Haddad, C. F. (2005). Reproductive activity and vocalizations of Leptodactylus labyrinthicus (Anura: Leptodactylidae) in southeastern Brazil. *Biota Neotropica*, *5*(2), 119-129.

Zimmerman, B. L. (1983). A comparison of structural features of calls of open and forest habitat frog species in the central amazon. *Herpetologica*, *39*(3), 235–246.

Zimmermann, B., and W. Hödl. (1983). Distinction of Phrynohyas resinifictrix (Goeldi, 1907) from Phrynohyas venulosa (Laurenti, 1768) based on acoustical and behavioural parameters (Amphibia, Anura, Hylidae). *Zoologischer Anzeiger* 211:341–352.

Zimmerman, B.L., and J.P. Bogart. (1988). Ecology and calls of four species of Amazonian forest frogs. *Journal of Herpetology* 22: 97–108.

Zweifel, R. G. (1972). Results of the Archbold Expeditions. No. 97. A revision of the frogs of the subfamily Asterophryinae, Family Microhylidae. *Bulletin of the American Museum of Natural History* 148: 411–546.

Zweifel, R. G. (1985). Australian frogs of the family Microhylidae. *Bulletin of the American Museum of Natural History* 182: 265–388.

Zweifel, R. G. (2000). Partition of the Australopapuan microhylid frog genus *Sphenophryne* with descriptions of new species. *Bulletin of the American Museum of Natural History* 253: 1–130. Available for anonymous download from http://digitallibrary.amnh.org/handle/2246/1600.

Zweifel, R. G., & Allison, A. (1982). A new montane microhylid frog from Papua New Guinea, and comments on the status of the genus Aphantophryne. *American Museum novitates*; no. 2723.

Zweifel, R. G., & Parker, F. (1989). New species of microhylid frogs from the Owen Stanley Mountains of Papua New Guinea and resurrection of the genus Aphantophryne. *American Museum novitates*; no. 2954.

Zweifel, Richard G., Menzies, J. I., and Price, D. (2003). Systematics of microhylid frogs, genus *Oreophryne*, from the North Coast Region of New Guinea. *American Museum Novitates* 3415:1–31.

Zweifel, R. G., Cogger, H. G., & Richards, S. J. (2005). Systematics of microhylid frogs, genus Oreophryne, living at high elevations in New Guinea. *American Museum Novitates*, no. 3495, 1-25.
