# Supplementary material for: Allometric escape from acoustic constraints is rare for frog calls
Source: Ecol Evol. 2020 Mar 7;10(8):3686–95. doi: 10.1002/ece3.6155 (PMC7160179; doi:10.1002/ece3.6155)
Supplement: Supplementary file 3 — Appendix S3 [file ECE3-10-3686-s003.docx]

**Appendix 1: Additional results of the PASTIS and results using data-only phylogeny.**

**Diversity of sound frequency and body size across frogs.** Body size explains a large proportion of the variation in advertisement call frequency (r^2^ = 0.51 for the PASTIS, Fig. 1; r^2^ = 0.54 for the data-only, Fig. S1). The mean Dominant frequency ranges from 200 Hz (*Chiromantis kelleri* and *Heleioporus barycragus*) to 16,590 Hz (*Huia cavitympanum*) and body size ranges from 8 mm (*Brachycephalus hermogenesi*) to 165 mm (*Pyxicephalus adspersus*). *Brachycephalus hermogenesi* have the greatest ratio of frequency to body size (850 Hz:1 mm), while *Pyxicephalus adspersus* the smallest one (1.35 Hz:1 mm).

**Model comparison and parameter estimates.** In preliminary analyses we tested whether the coding strategy for calling site would affect the overall results. We ran on test coding calling site as 1) ground, 2) perched, and 3) water, and another test coding calling sites as three separate factors and as binary. The shifts identified in both analyses were the same (results not shown for the binary coding). Furthermore, in the runs including just the priors no shifts were identified when running prior-only analyses. Across all models tested, the two chains converging (Fig. S2) were combined to increase ESS values and improve parameter estimates of analyses using the PASTIS phylogeny (Table S1-S3) and data-only phylogeny (Table S4-S6).

The model including as predictors body size, calling site, and interaction between these two predictors (θ_DF_ ~ β_body size_ + β_sit_ + β_body size*sit_) had stronger support over the competing models (BF > 10), both in the analyses using the PASTIS and data-only phylogenies (Fig. S3). This result shows that calling site contributes to explain the variation in sound frequency and body size across the frog phylogeny (Fig. S4). Overall, species calling perched or from the ground have smaller body size and call at higher frequency relative to species calling from the in the water (Fig. S1 and S5).

The results of the best fit model using the PASTIS and data-only phylogenies are equivalent. However, given to the reduced taxon sampling in the data-only phylogenies, we recovered a smaller number of shifts. Using the PASTIS, the best fit model identified 82 shifts (see shiftsums.NNN.0.1$descendents in the Appendix 3) from the background regime with posterior probability higher than 0.1. After increasing the posterior probability threshold to 0.7 we recover 26 shifts (see shiftsums.NNN.0.7$descendents in the Appendix 3). After excluding shifts with less than three species, we used the pANCOVA to further test the shifts identified by the best fit bayou model. At posterior probability 0.1, out of those 82 shifts four had significant support in the pANCOVA for the Full Model considering distinct intercept and slope relative to the remaining frog species. In contrast, using the data-only the same model at posterior probability 0.7 we identified 10 shifts, out of those only two had support for the Full Model in the pANCOVA – poison frogs and southeast Asian ranids, as well as identified in the PASTIS. In the prior-only analyses we did not recover any shift, not even at 0.1 posterior probability, which show that the results are driven by the signal in the data.

Below are the species included in the major shifts highlighted in the main text. The taxonomy follows Jetz and Pyron (2018).

- Red: *Meristogenys orphnocnemis*, *Meristogenys amoropalamus*, *Meristogenys jerboa*, *Meristogenys kinabaluensis*, *Huia masonii*, and *Huia cavitympanum*.
- Green: *Rana temporaria*, *Rana pyrenaica*, *Rana iberica*, *Rana dalmatina*, *Rana sierrae*, *Rana muscosa*, *Rana aurora*, *Rana cascadae*, *Rana draytonii*, *Rana boylii*, *Rana luteiventris*, *Rana pretiosa*, *Rana taylori*, *Rana forreri*, *Rana yavapaiensis*, *Rana onca*, *Rana berlandieri*, *Rana blairi*, *Rana sphenocephala*, *Rana palustris*, *Rana areolata*, *Rana capito*, *Rana chiricahuensis*, *Rana pipiens*, *Rana vaillanti*, *Rana palmipes, Rana warszewitschii*, *Rana vibicaria*, *Rana tarahumarae*, *Rana sylvatica*, *Rana okaloosae*, *Rana clamitans*, *Rana catesbeiana*, *Rana heckscheri*, *Rana grylio*, *Rana septentrionalis*, *Rana virgatipes*, *Odorrana morafkai*, *Odorrana graminea*, *Odorrana livida*, *Odorrana hosii*, *Odorrana tormota*, *Odorrana amamiensis*, *Odorrana supranarina*, *Odorrana utsunomiyaorum*, *Odorrana schmackeri*, *Odorrana grahami*, *Odorrana yizhangensis*, *Babina chapaensis*, *Babina daunchina*, *Babina adenopleura*, and *Babina pleuraden*.
- Orange: *Dendropsophus haraldschultzi*, *Dendropsophus sanborni*, *Dendropsophus rubicundulus*, *Dendropsophus tritaeniatus*, *Dendropsophus minusculus*, *Dendropsophus joannae*, *Dendropsophus juliani*, *Dendropsophus meridianus*, *Dendropsophus acreanus*, *Dendropsophus bipunctatus*, *Dendropsophus berthalutzae*, *Dendropsophus soaresi*, *Dendropsophus nanus, Dendropsophus riveroi*, *Dendropsophus branneri*, *Dendropsophus phlebodes*, *Dendropsophus microcephalus*, *Dendropsophus rhodopeplus*, *Dendropsophus robertmertensi*, *Dendropsophus sartori*, *Dendropsophus leali*, *Dendropsophus anceps*, *Dendropsophus luteoocellatus*, *Dendropsophus nahdereri*, *Dendropsophus minutus*, *Dendropsophus bifurcus*, *Dendropsophus sarayacuensis*, *Dendropsophus ebraccatus*, *Dendropsophus ruschii*, *Dendropsophus leucophyllatus, Dendropsophus triangulum*, *Dendropsophus elegans*, *Dendropsophus timbeba*, *Dendropsophus elianeae*, *Dendropsophus werneri*, *Dendropsophus koechlini*, *Dendropsophus coffeus*, *Dendropsophus parviceps*, *Dendropsophus brevifrons*, *Dendropsophus delarivai*, *Dendropsophus rossalleni*, *Dendropsophus decipiens, Dendropsophus giesleri*, *Dendropsophus shiwiarum*, *Dendropsophus studerae*, *Dendropsophus microps*, *Dendropsophus carnifex*, *Dendropsophus xapuriensis*, *Dendropsophus haddadi*, *Dendropsophus luddeckei*, *Dendropsophus labialis*, *Dendropsophus gryllatus*, *Dendropsophus oliveirai*, *Dendropsophus jimi*, *Dendropsophus bokermanni*, *Dendropsophus seniculus*, *Dendropsophus marmoratus*, *Dendropsophus melanargyreus*, and *Dendropsophus cruzi*.
- Blue: *Hyloxalus infraguttatus*, *Hyloxalus awa*, *Hyloxalus toachi*, *Hyloxalus elachyhistus*, *Hyloxalus insulatus*, *Hyloxalus azureiventris*, *Hyloxalus craspedoceps*, *Hyloxalus nexipus*, *Hyloxalus anthracinus*, *Hyloxalus vertebralis*, *Hyloxalus delatorreae*, *Hyloxalus pulchellus*, *Hyloxalus sauli*, *Hyloxalus bocagei*, *Hyloxalus italoi*, *Hyloxalus maculosus*, *Hyloxalus yasuni*, *Hyloxalus chocoensis*, *Hyloxalus subpunctatus*, *Hyloxalus sordidatus*, *Ranitomeya fantastica*, *Ranitomeya summersi*, *Ranitomeya benedicta*, *Ranitomeya reticulata*, *Ranitomeya uakarii*, *Ranitomeya ventrimaculata*, *Ranitomeya yavaricola*, *Ranitomeya variabilis*, *Ranitomeya defleri*, *Ranitomeya imitator*, *Ranitomeya sirensis*, *Andinobates virolinensis*, *Andinobates bombetes*, *Andinobates claudiae*, *Andinobates minutus*, *Excidobates mysteriosus*, *Excidobates captivus*, *Oophaga pumilio*, *Oophaga lehmanni*, *Oophaga sylvatica*, *Oophaga histrionica*, *Oophaga granulifera*, *Dendrobates tinctorius*, *Dendrobates leucomelas*, *Dendrobates truncatus*, *Dendrobates auratus*, *Adelphobates* *galactonotus*, *Adelphobates castaneoticus*, *Phyllobates terribilis*, *Phyllobates aurotaenia*, *Phyllobates bicolor*, *Phyllobates vittatus*, *Phyllobates lugubris*, *Ameerega cainarachi*, *Ameerega smaragdina*, *Ameerega petersi*, *Ameerega macero*, *Ameerega altamazonica*, *Ameerega pulchripecta*, *Ameerega trivittata*, *Ameerega simulans*, *Ameerega berohoka*, *Ameerega hahneli*, *Ameerega erythromos*, *Ameerega picta*, *Ameerega flavopicta*, *Ameerega braccata*, *Ameerega yungicola*, *Ameerega ignipedis*, *Ameerega pepperi*, *Ameerega yoshina*, *Ameerega bassleri*, *Ameerega ingeri*, *Ameerega pongoensis*, *Ameerega boliviana*, *Ameerega silverstonei*, *Colostethus fraterdanieli*, *Colostethus fugax*, *Colostethus argyrogaster*, *Colostethus jacobuspetersi*, *Colostethus panamansis*, *Colostethus inguinalis*, *Colostethus pratti*, *Silverstoneia flotator*, *Silverstoneia nubicola*, *Epipedobates tricolor*, *Epipedobates anthonyi*, *Epipedobates machalilla*, *Epipedobates* *espinosai*, *Epipedobates boulengeri*, *Mannophryne vulcano*, *Mannophryne orellana*, *Mannophryne venezuelensis*, *Mannophryne trinitatis*, *Mannophryne leonardoi*, *Mannophryne olmonae*, *Mannophryne riveroi*, *Mannophryne urticans*, *Mannophryne* *herminae*, *Mannophryne yustizi*, *Mannophryne collaris*, *Mannophryne cordilleriana*, *Aromobates ericksonae*, *Aromobates cannatellai*, *Aromobates saltuensis*, *Aromobates* *meridensis*, *Aromobates ornatissimus*, *Anomaloglossus praderioi*, *Anomaloglossus kaiei*, *Anomaloglossus degranvillei*, *Anomaloglossus beebei*, *Anomaloglossus rufulus*, *Anomaloglossus stepheni*, Anomaloglossus baeobatrachus, *Anomaloglossus verbeeksnyderorum*, *Rheobates palmatus*, *Allobates humilis*, *Allobates* *chalcopis*, *Allobates trilineatus*, *Allobates subfolionidificans*, *Allobates melanolaemus*, *Allobates insperatus*, *Allobates algorei*, *Allobates granti*, *Allobates olfersioides*, *Allobates* *marchesianus*, *Allobates caeruleodactylus*, *Allobates juanii*, *Allobates femoralis*, *Allobates zaparo*, *Allobates hodli*, *Allobates crombiei*, *Allobates flaviventris*, *Allobates* *brunneus*, *Allobates alessandroi*, *Allobates myersi*, *Allobates goianus*, *Allobates* *masniger*, *Allobates nidicola*, *Allobates kingsburyi*, and *Allobates talamancae*.

In the context of linear models, we estimated the marginal contribution of each predictor (body size, calling site, and the interaction) by calculating the difference between R^2^ across models. In the model θ_DF_ ~ β_body size_, body size explains 0.5105 of the variance in DF. In the model θ_DF_ ~ β_body size_ + β_sit_, body size and calling site explain 0.5116 of the variance in DF; thus, calling site represents an improvement of 0.0011 in explaining the variance in the data. In the model θ_DF_ ~ β_body size_ + β_sit_ + β_body size*sit_, body size, calling site, and the interaction explain 0.5184 of the variance in DF, so the interaction between body size and sound frequency represents an improvement of 0.0068.


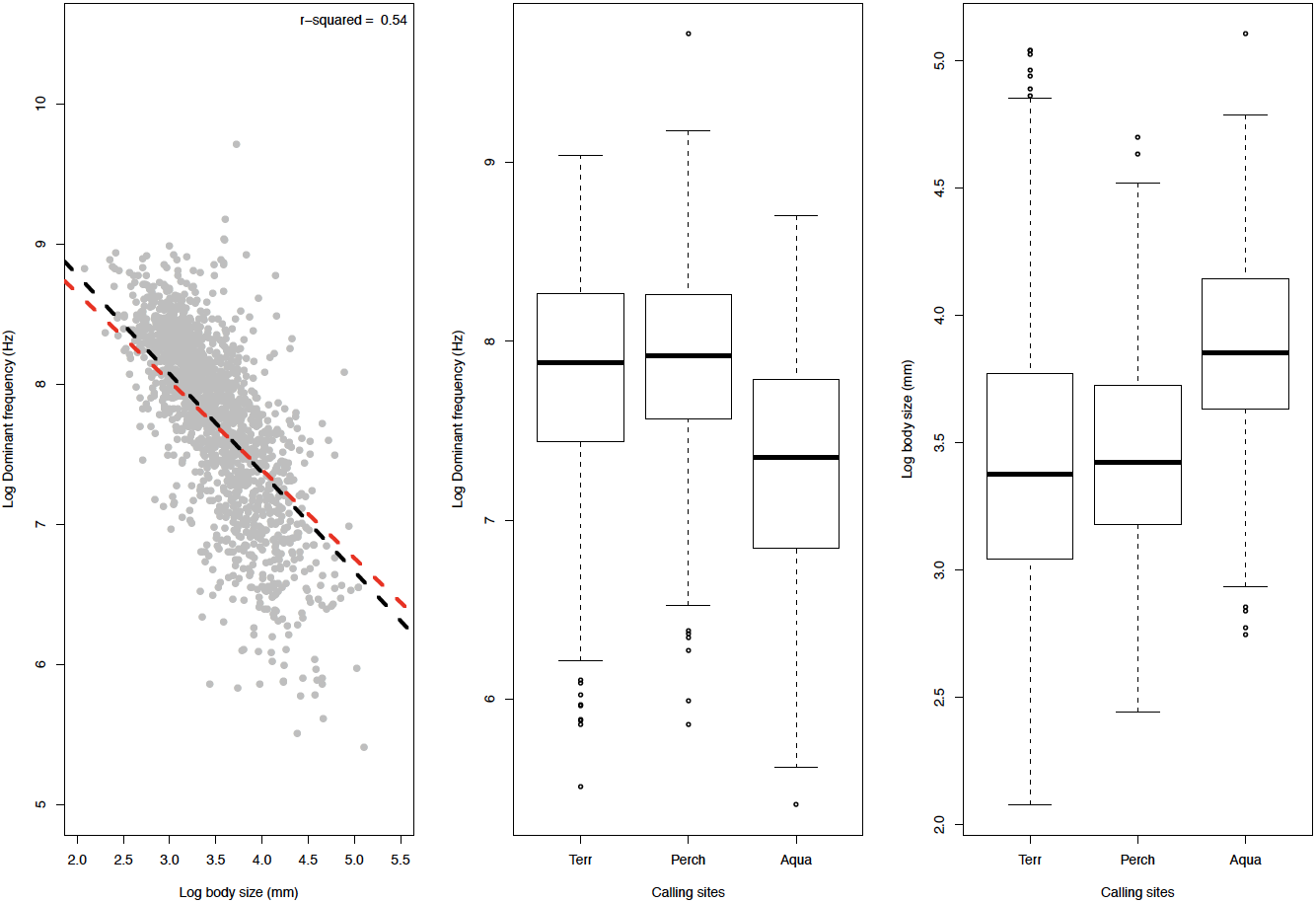


**Fig. S1.** Allometric relationship between log dominant frequency (Hz) and log body size (mm) across 1,610 frog species included in the data-only phylogeny. The right panel shows the phylogenetic generalized least squares in red and linear regression in black. Note that body size alone explains 54% of the variation in dominant frequency (inset: value of r2). The central panel shows the relationship of log dominant frequency and calling sites. The right panel shows the relationship of log body size and calling sites. Note that species calling from terrestrial sites or perches have smaller body size and higher frequency calls than species calling from water.

Fig. S2. Convergence plots comparing posterior branch lengths of two chains combined using R Gelman. Top row show results using the PASTIS phylogeny (2,176 species) and in the bottom row are the results using the data-only phylogeny (1,610 species). First column shows results of the model θ_DF_ ~ β_svl_, second column shows the results of model θ_DF_ ~ β_svl_ + β_sit_, and third column shows the results of model θ_DF_ ~ β_svl_ + β_sit_ + β_svl*sit_. Note that in data-only phylogeny for more complex models, convergence is not as good as in the PASTIS due to reduced species sampling.

Fig. S3. Model comparison using Bayes Factor. Horizontal line indicates threshold to strongly reject competing models (BF > 10). Models in the x-axis from 1 to 3 represent: 1) θ_DF_ ~ β_svl_ + β_sit_ + β_svl*sit_ *vs* θ_DF_ ~ β_svl_; 2) θ_DF_ ~ β_svl_ + β_sit_ + β_svl*sit_ *vs* θ_DF_ ~ β_svl_ + β_sit_; 3) θ_DF_ ~ β_svl_ *vs* θ_DF_ ~ β_svl_ + β_sit_; using the PASTIS phylogeny. In models 4 to 6 we compare the results of models as in 1 to 3, but using the data-only phylogeny.


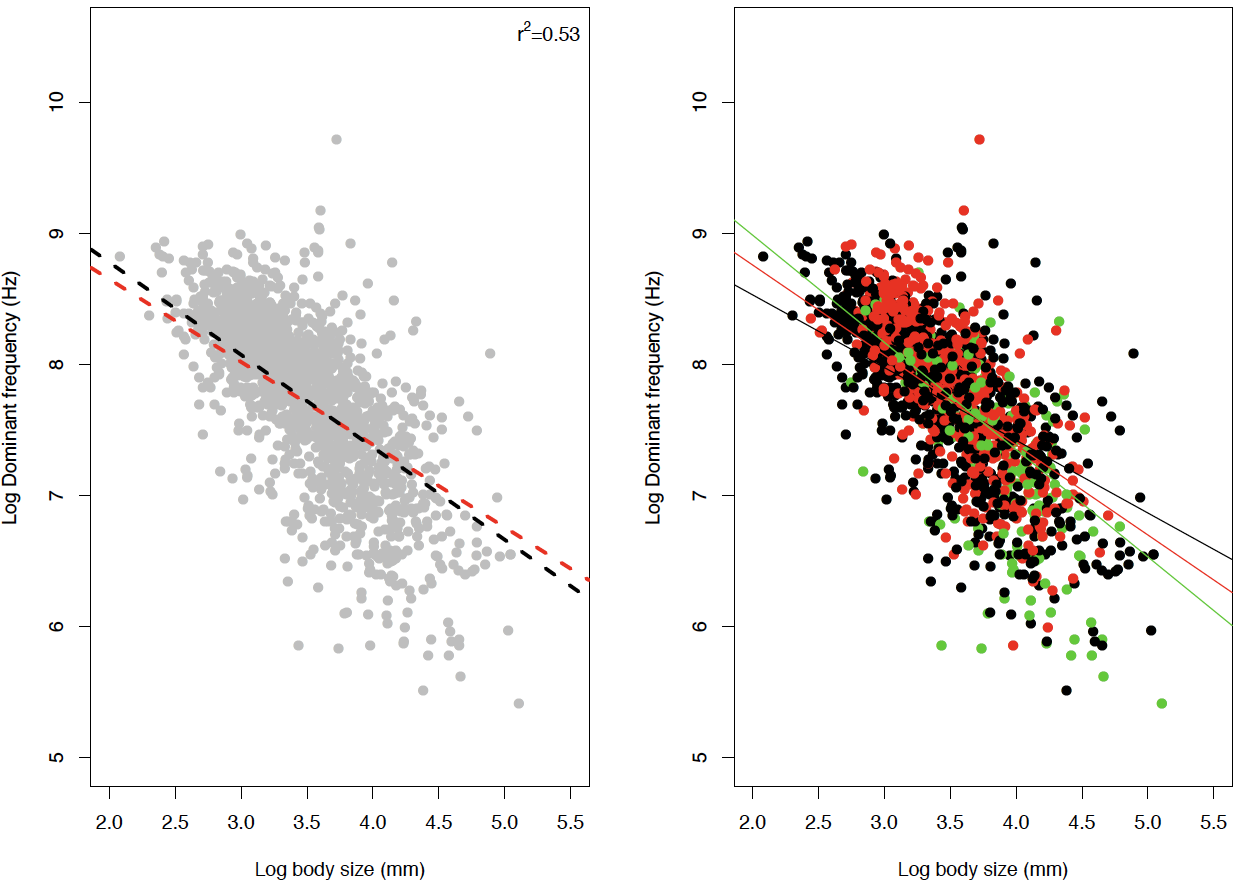


Fig. S4. Allometric relationship between log dominant frequency (Hz) and log body size (mm) across 1,610 frog species included in the data-only phylogeny. The panel shows the different allometric scaling for each calling site estimated by the best fit model in bayou. In black, species calling from the ground; in red, species that perch while emitting advertisement calls; and in green, species that call while sitting, swimming, or submersed in water. We recovered very similar results between the PASTIS and data-only phylogeny.


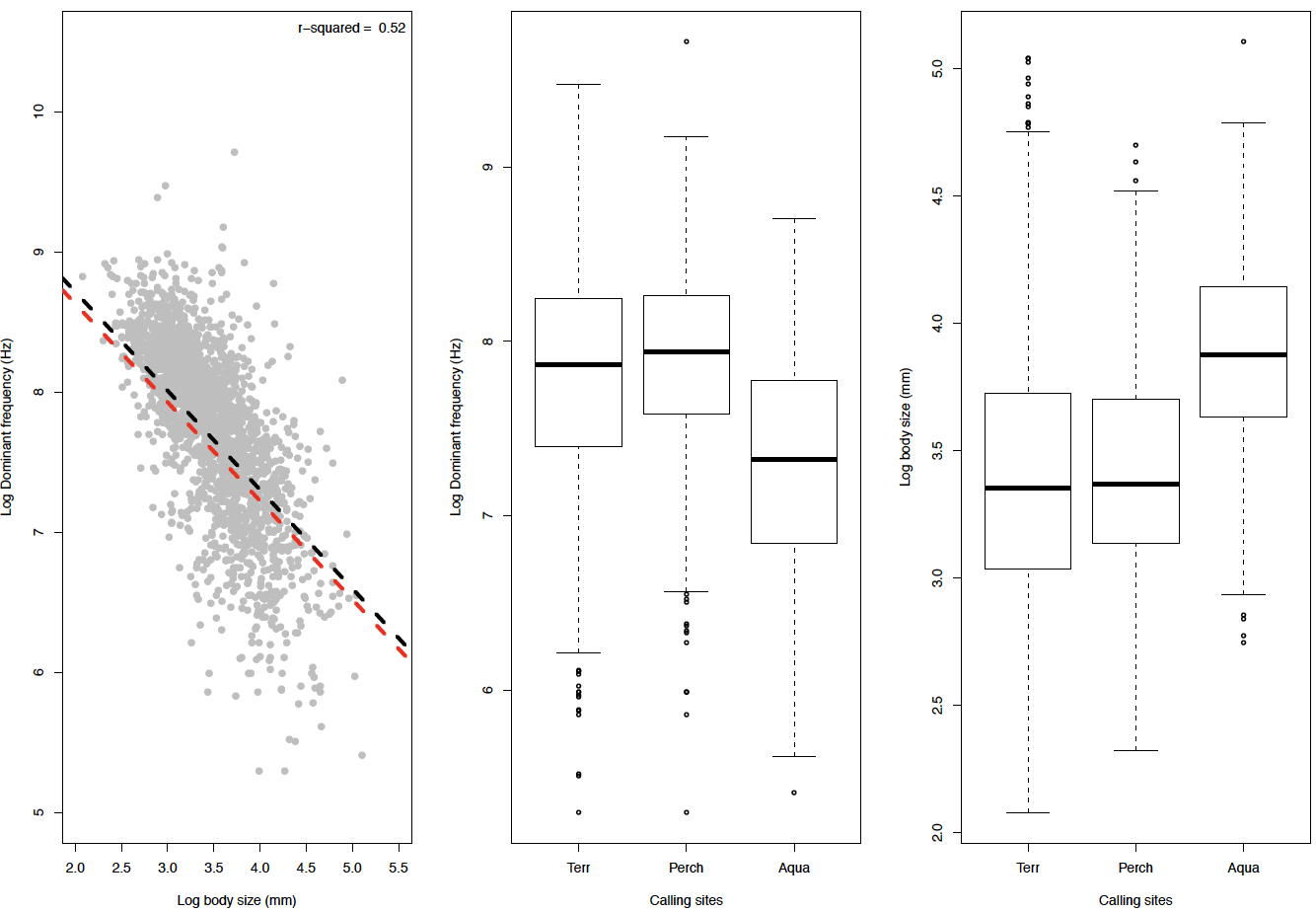


**Fig. S5.** Allometric relationship between log Dominant frequency (Hz) and log body size (mm) across 2,176 frog species included in the PASTIS phylogeny. The right panel shows the phylogenetic generalized least squares in red and linear regression in black. Note that body size alone explains 52% of the variation in Dominant frequency (inset: value of r2). The central panel shows the relationship of log Dominant frequency and calling sites. The right panel shows the relationship of log body size and calling sites. Note that species calling from terrestrial sites or perches have smaller body size and higher frequency calls than species calling from water.

Table S1. Parameter estimates of the model θ_DF_ ~ β_svl_ using the PASTIS phylogeny.

| Parameters | Mean | SD | HPD95Lower | HPD95Upper | ESS |
| --- | --- | --- | --- | --- | --- |
| lnL | -712.06 | 96.81 | -804.334 | -619.277 | 239.52 |
| prior | -421.51 | 34.13 | -489.176 | -356.867 | 184.12 |
| α | 78.76 | 139.26 | 3.563 | 214.689 | 47.73 |
| σ^2^ | 17.91 | 32.15 | 0.804 | 48.227 | 47.58 |
| K | 54.55 | 4.98 | 45.000 | 64.000 | 189.89 |
| nθ | 55.55 | 4.98 | 46.000 | 65.000 | 189.89 |
| θ_root_ | 9.11 | 1.03 | 6.767 | 10.072 | 7.66 |
| β_root (body size)_ | -0.44 | 0.23 | -0.675 | 0.095 | 9.08 |

HPD=Highest Posterior Density.

Table S2. Parameter estimates of the model θ_DF_ ~ β_svl_ + β_sit_ using the PASTIS phylogeny.

| Parameters | Mean | SD | HPD95Lower | HPD95Upper | ESS |
| --- | --- | --- | --- | --- | --- |
| lnL | -713.51 | 120.86 | -869.860 | -590.419 | 98.04 |
| prior | -434.45 | 37.77 | -502.589 | -358.015 | 116.46 |
| α | 76.33 | 99.90 | 8.053 | 196.045 | 38.17 |
| σ^2^ | 18.01 | 26.61 | 1.726 | 43.253 | 39.32 |
| K | 59.02 | 5.52 | 48.000 | 69.000 | 113.54 |
| nθ | 60.02 | 5.52 | 49.000 | 70.000 | 113.54 |
| θ_root_ | 8.19 | 0.67 | 7.069 | 9.353 | 22.88 |
| β_root (body size)_ | -0.44 | 0.23 | -0.506 | 0.104 | 9.08 |
| β_root (sit)_ | 0.04 | 0.11 | -0.205 | 0.236 | 54.57 |

HPD=Highest Posterior Density.

**Table S3. Parameter estimates of the model θ_DF_ ~ β_svl_ + β_sit_ + β_svl*sit_ using the PASTIS phylogeny.**

| Parameters | Mean | SD | HPD95Lower | HPD95Upper | ESS |
| --- | --- | --- | --- | --- | --- |
| lnL | -677.26 | 1121.31 | -753.09 | -588.49 | 24052.08 |
| prior | -352.34 | 28.79 | -408.43 | -300.66 | 236.29 |
| α | 83.22 | 76.64 | 3.67 | 244.62 | 57.35 |
| σ^2^ | 17.88 | 16.28 | 0.77 | 52.13 | 57.51 |
| K | 49.48 | 4.31 | 41.00 | 57.00 | 214.40 |
| nθ | 50.48 | 4.31 | 42.00 | 58.00 | 214.40 |
| θ_root_ | 9.24 | 0.35 | 8.91 | 9.69 | 15.99 |
| β_root (body size)_ | -0.45 | 0.10 | -0.58 | -0.37 | 12.62 |
| β_root (sit)_ | 0.49 | 0.14 | 0.23 | 0.67 | 21.11 |
| β_root (body size*sit)_ | -0.13 | 0.04 | -0.18 | -0.05 | 34.87 |

HPD=Highest Posterior Density.

**Table S4. Parameter estimates of the model θ_DF_ ~ β_svl_ using the data-only phylogeny.**

| Parameters | Mean | SD | HPD95Lower | HPD95Upper | ESS |
| --- | --- | --- | --- | --- | --- |
| lnL | -581.43 | 39.22 | -634.20 | -529.20 | 516.98 |
| prior | -293.25 | 73.34 | -379.80 | -105.08 | 47.31 |
| α | 75.20 | 105.78 | 0.02 | 231.70 | 192.55 |
| σ^2^ | 18.35 | 27.23 | 0.01 | 56.35 | 221.96 |
| K | 38.43 | 11.30 | 10.00 | 51.00 | 47.37 |
| nθ | 39.43 | 11.30 | 11.00 | 52.00 | 47.37 |
| θ_root_ | 9.72 | 0.33 | 9.33 | 10.57 | 27.03 |
| β_root (body size)_ | -0.58 | 0.09 | -0.82 | -0.46 | 27.76 |

HPD=Highest Posterior Density.

Table S5. Parameter estimates of the model θ_DF_ ~ β_svl_ + β_sit_ using the data-only phylogeny.

| Parameters | Mean | SD | HPD95Lower | HPD95Upper | ESS |
| --- | --- | --- | --- | --- | --- |
| lnL | -589.59 | 251.33 | -656.18 | -514.20 | 5570.71 |
| prior | -332.63 | 39.70 | -411.65 | -260.61 | 109.49 |
| α | 88.99 | 123.70 | 1.30 | 313.48 | 77.32 |
| σ^2^ | 21.58 | 29.70 | 0.31 | 75.82 | 73.63 |
| K | 47.07 | 6.47 | 35.00 | 59.00 | 99.49 |
| nθ | 48.07 | 6.47 | 36.00 | 60.00 | 99.49 |
| θ_root_ | 9.15 | 0.61 | 7.67 | 9.81 | 11.57 |
| β_root (body size)_ | -0.43 | 0.14 | -0.63 | -0.10 | 11.12 |
| β_root (sit)_ | 0.03 | 0.08 | -0.13 | 0.21 | 56.66 |

HPD=Highest Posterior Density.

**Table S6. Parameter estimates of the model θ_DF_ ~ β_svl_ + β_sit_ + β_svl*sit_ using the data-only phylogeny.**

| Parameters | Mean | SD | HPD95Lower | HPD95Upper | ESS |
| --- | --- | --- | --- | --- | --- |
| lnL | -554.36 | 54.48 | -607.58 | -496.03 | 551.25 |
| prior | -263.93 | 27.89 | -317.97 | -211.47 | 350.12 |
| α | 100.88 | 139.64 | 3.04 | 326.86 | 91.94 |
| σ^2^ | 23.42 | 32.08 | 0.68 | 76.00 | 92.10 |
| K | 37.95 | 4.47 | 29.00 | 46.00 | 408.90 |
| nθ | 38.95 | 4.47 | 30.00 | 47.00 | 408.90 |
| θ_root_ | 8.72 | 0.72 | 7.34 | 9.48 | 16.75 |
| β_root (body size)_ | -0.37 | 0.13 | -0.52 | -0.08 | 28.76 |
| β_root (sit)_ | 0.40 | 0.23 | -0.06 | 0.71 | 23.05 |
| β_root (body size*sit)_ | -0.10 | 0.07 | -0.18 | 0.05 | 12.48 |

HPD=Highest Posterior Density.
